# Improving early childhood development in the context of the nurturing care framework in Kenya: A policy review and qualitative exploration of emerging issues with policy makers

**DOI:** 10.3389/fpubh.2022.1016156

**Published:** 2022-09-27

**Authors:** Mary Abboah-Offei, Patrick Amboka, Margaret Nampijja, George Evans Owino, Kenneth Okelo, Patricia Kitsao-Wekulo, Ivy Chumo, Ruth Muendo, Linda Oloo, Maryann Wanjau, Elizabeth Mwaniki, Maurice Mutisya, Emma Haycraft, Robert Hughes, Paula Griffiths, Helen Elsey

**Affiliations:** ^1^School of Health and Life Sciences, University of the West of Scotland, London, United Kingdom; ^2^African Population and Health Research Center, APHRC Campus, Nairobi, Kenya; ^3^Africa Early Childhood Network, Nairobi, Kenya; ^4^Community Engagement Associate, UNICEF, Greater Houston, TX, United States; ^5^School of Sport, Exercise and Health Sciences, Loughborough University, Loughborough, United Kingdom; ^6^Department of Population Health, Faculty of Epidemiology and Population Health, London School of Hygiene and Tropical Medicine, London, United Kingdom; ^7^Department of Health Sciences, University of York, York, United Kingdom

**Keywords:** early childhood development, nurturing care framework, nurturing care, children under five, policy, Kenya

## Abstract

**Introduction:**

The Nurturing Care Framework (NCF) describes “nurturing care” as the ability of nations and communities to support caregivers and provide an environment that ensures children's good health and nutrition, protects them from threats, and provides opportunities for early learning through responsive and emotionally supportive interaction. We assessed the extent to which Kenyan government policies address the components of the NCF and explored policy/decision makers' views on policy gaps and emerging issues.

**Methods:**

A search strategy was formulated to identify policy documents focusing on early childhood development (ECD), health and nutrition, responsive caregiving, opportunities for early learning and security and safety, which are key components of the NCF. We limited the search to policy documents published since 2010 when the Kenya constitution was promulgated and ECD functions devolved to county governments. Policy/decision-maker interviews were also conducted to clarify emerging gaps from policy data. Data was extracted, coded and analyzed based on the components of the NCF. Framework analysis was used for interview data with NCF being the main framework of analysis. The Jaccard's similarity coefficient was used to assess similarities between the themes being compared to further understand the challenges, successes and future plans of policy and implementation under each of the NCF domains.

**Results:**

127 policy documents were retrieved from government e-repository and county websites. Of these, *n* = 91 were assessed against the inclusion criteria, and *n* = 66 were included in final analysis. The 66 documents included 47 County Integrated Development Plans (CIDPs) and 19 national policy documents. Twenty policy/decision-maker interviews were conducted. Analysis of both policy and interview data reveal that, while areas of health and nutrition have been considered in policies and county level plans (coefficients >0.5), the domains of early learning, responsive caregiving and safety and security face significant policy and implementation gaps (coefficients ≤ 0.5), particularly for the 0–3 year age group. Inconsistencies were noted between county level implementation plans and national policies in areas such as support for children with disabilities and allocation of budget to early learning and nutrition domains.

**Conclusion:**

Findings indicate a strong focus on nutrition and health with limited coverage of responsive caregiving and opportunities for early learning domains. Therefore, if nurturing care goals are to be achieved in Kenya, policies are needed to support current gaps identified with urgent need for policies of minimum standards that provide support for improvements across all Nurturing Care Framework domains.

## Introduction

More than 250 million children (43%) younger than 5 years in low- and middle-income countries (LMICs) do not reach their developmental potential due to adversities in the early years including poverty, undernutrition and a lack of nurturing care and stimulation (1–3). The Lancet Early Childhood Development (ECD) Series defines “nurturing care” as a central organizing principle for policy and practice devoted to addressing the core problem that the children in LMICs are at risk of (4). The Nurturing Care Framework (NCF) describes “nurturing care” as the ability of nations and communities to support caregivers and provide an environment that ensures children's good health and nutrition, protects them from threats, and gives them opportunities for early learning through responsive and emotionally supportive interaction, see Table 1 (1).

**Table 1:** Detailed components of the nurturing care framework (1).

**Good health:**	**Adequate nutrition:**	**Responsive caregiving:**	**Opportunities for early learning:**	**Security and safety:**
a. Family planning b. Immunization for mothers and children c. Prevention and cessation of smoking, alcohol and substance use d. Prevention of mother-to-child transmission of HIV e. Support for caregivers' mental health f. Antenatal and childbirth care g. Prevention of preterm births h. Essential care for new-born babies, with extra care for small and sick babies i. Kangaroo care for low-birthweight babies j. Support for timely and appropriate care seeking for sick children k. Integrated management of childhood illness l. Early detection of disabling conditions (such as problems with sight and hearing) m. Care for children with developmental difficulties and disabilities	a. Maternal nutrition b. Support for early initiation, exclusive breastfeeding and continued breastfeeding after 6 months c. Support for appropriate complementary feeding and for transition to a healthy family diet d. Micronutrient supplementation for mother and child, as needed e. Fortification of staple foods f. Growth monitoring and promotion, including intervention and referral when indicated g. Deworming h. Support for appropriate child feeding during illness i. Management of moderate and severe malnutrition as well as being overweight or obese	a. Skin-to-skin contact immediately after birth b. Kangaroo care for low-birthweight babies c. Rooming-in for mothers and young infants and feeding on demand d. Responsive feeding e. Interventions that encourage play and communication activities of caregiver with the child f. Interventions to promote caregiver sensitivity and responsiveness to children's cues g. Support for caregivers' mental health h. Involving fathers, extended family and other partners i. Social support from families, community groups and faith communities	a. Information, support and counseling about opportunities for early learning, including the use of common household objects and home-made toys b. Play, reading and story-telling groups for caregivers and children c. Book sharing d. Mobile toy and book libraries e. Good-quality day care for children, and pre-primary education f. Storytelling of elders with children g. Using local language in children's daily care	a. Birth registration b. Provision of safe water and sanitation c. Good hygiene practices at home, at work and in the community d. Prevention and reduction of indoor and outdoor air pollution e. Clean environments free of hazardous chemicals f. Safe family and play spaces in urban and rural areas g. Prevention of violence by intimate partners and in families, as well as services for addressing it h. Social care services i. Cash or in-kind transfers and social insurance j. Supporting family care and foster care over institutional care

The NCF aims to inspire multiple sectors including health, nutrition, education, labor, finance, water and sanitation, and social and child protection to work together in new ways to address the needs of the youngest children (1). It articulates the importance of responsive caregiving and early learning as integral components of good quality care for young children (see Table 1). It illustrates how existing programmes can be enhanced to be more comprehensive in addressing young children's needs. The Framework promotes the use of local assets, adaptation to the local context, and ownership at community level. It describes the foundations, actions and government leadership that must be in place for all children to reach their potential (1). Translating the NCF into policy and practice in LMICs is a prerequisite for achieving progress toward child health and development and is fundamental to several Sustainable Development Goals. While progress has been made in child survival with a 53% reduction in under five child mortality from 1990 to 2015, under five mortality remains unacceptably high with more than 5 million children dying before their 5th birthday in 2020 (5).

The Early Childhood Development (ECD) Index has been designed to measure progress and generate action toward the SDGs, particularly SDG 4.2 of ensuring that all girls and boys have access to quality ECD, care and pre-primary education by 2030 (6). This has revealed the extent of the global ECD challenge with recent analysis of national survey data from 63 LMICs finding 38.7% of children in South and East Africa to have suspected developmental delay and 32.6% in South Asia (7). The causes of developmental delay are closely linked to poverty, including poor nutrition, recurrent infectious disease episodes, lack of stimulation and inadequate care (8, 9). National surveys in LMICs shed further light on the challenges families are facing in providing the nurturing caring required for healthy ECD. These challenges are particularly stark and have a clear social gradient, with between 5–17% and 7–29% of under-5 children from low-income families left alone for at least 1 day a week in East and Southern Africa and in South Asia, respectively (10). Furthermore, the World Bank estimates that 72% of all children below primary-school-entry age need some form of childcare globally, however 59% of these children do not currently have access (11).

Changing working patterns driven by migration to urban areas is exacerbating these challenges as low-income families, particularly women, must work long hours outside the home and this has created demand for non-parental childcare in many LMICs (12, 13). There are limited options for childcare as some women either take their children to work or leave them at home alone (14, 15). This undermines child health, already compounded because of their living conditions and inadequate access to quality health care faced by the urban poor (16–19), with greater exposures to injuries (10, 20), poor nutrition (13, 21, 22), poor hygiene (23, 24) and low uptake of child health programs (25–27). This is demonstrated through statistics that only 58% of children in Kenya's slums are immunized (28), with particularly low uptake among rural to urban migrants (29). The Kenyan government use a community health volunteers (CHVs) model to provide care and support for families through health and nutrition screening within each county (30). These CHVs are supervised by community health assistants (CHAs), who have received a formal health and nutrition training. Given the increased global focus on nurturing care, and the urgent need to address healthy ECD, understanding how LMIC governments have established policy frameworks to steer the response to these challenges is important. Naumen et al. emphasize the 3-fold need for policies on ECD, to (i) present a vision for the country's children (ii) clarify the responsibility of different ministries and actors, with particular importance given to the multi-sectoral responses needed, and (iii) to define roles for public and private sectors, particularly in relation to funding and service provision (31).

In Kenya, child health and development face many challenges particularly in deprived urban neighborhoods and remote rural communities. Nationally, under five mortality remain stubbornly high with 41.9 deaths per 1,000 live births in 2020, and 19.4% of under 5s are stunted (5, 32). As the NCF is a relatively new framework, many countries including Kenya, are now in the process of using it to guide and strengthen their ECD policies and practices. Kenya was one of the first countries in sub-Saharan Africa to approve an integrated National ECD Policy Framework in 2006. The policy and subsequent Service Standard Guidelines aimed to serve as a coordination mechanism, defining roles of various stakeholders across government, agencies, communities, and parents (31). Promulgation of the Constitution in 2010 created a decentralized system of 47 counties. Counties were assigned the responsibility of early childhood education, and it has been argued that since then, the ECD sector in Kenya has not received adequate attention (33). The 2010 Constitution created a two-tier system of governance—national and devolved county governments that are distinct and interdependent. This governance landscape requires a paradigm shift in integrated development planning, which brings together the different development sectors who work together to produce 4-year County Integrated development Plans (CIDPs). The government of Kenya provides the greatest share of resources for the implementation of the CIDPs through the “equitable revenue share” based on weighted criteria for allocation across Counties based on poverty rate, population and area size. Further resources come from local levies (e.g., Road Maintenance Levy Fund) and loans and grants from development partners (34).

To establish the extent to which government policies and plans address the components of the NCF, particularly in relation to center-based childcare, and explore policy makers' views, we carried out a policy review as part of a study that explored feasible models to improve the quality of center-based childcare in informal settlements in Nairobi (35). The objectives for the review include: (i) To identify the extent to which the NCF is addressed in Kenyan national policies and county government plans; (ii) To identify the extent and characteristics of childcare center provision in Kenya as specified in county-level plans; and (iii) To explore, from the perceptions of decision-makers, the extent to which nurturing care, including quality center-based childcare is addressed within national and county level policies and plans.

## Methods

A search strategy was formulated to identify the extent to which the NCF is addressed in Kenyan national policies and county government plans. Following the analysis of the policies and plans, qualitative interviews were conducted with national policy makers and county-level decision-makers to explore the extent to which nurturing care, including quality center-based childcare has been addressed within national and county level policies and plans.

### Search strategy

As the components of the NCF focus on multiple sectors including health, nutrition, education, labor, finance, water and sanitation, and social and child protection (1), our search strategy also focused on identifying policies across corresponding sectors within the government of Kenya. We searched for CIDPs from all 47 of Kenya's counties.

#### Step 1—Searching

To retrieve policy documents, the first author (MA-O) conducted a primary search in the e-repository for Kenyan government documents, government ministries (health; education; gender, children, and social services; labor, social security and services; home affairs) websites, county websites and national councils' websites among others. Search terms used include policy (policies, guidelines, Acts, standards) and ECD (early childhood care, early childhood education, early childhood education center, early learning) and center-based care (daycare, daycare centers, childcare centers, pre-school) and terms related to under-fives. We limited the search to policy documents published since 2010 when the Kenya Constitution was promulgated and the ECD function devolved to county governments.

#### Inclusion and exclusion criteria

We included national policy documents and county integrated development plans (CIDPs) that addressed issues relating to ECD and components of the NCF (“good health”, “adequate nutrition”, “responsive caregiving”, “opportunities for early learning” and “safety and security”) for children under 5 years. These documents were published in English spanning from 2010 onwards (the Kenya constitution was promulgated and the ECD function devolved to county governments). All the most recent 4-year CIDPs from all counties in Kenya were included. Policy documents that focused on topical issues other than ECD and components of the NCF, those focusing on children above age 5 and published prior to, and beyond 2010, were excluded including those published in languages other than English.

#### Step 2—Sifting and sorting

The titles of all policy documents were first scanned and reviewed by two members of the project team (MA-O and HE), to remove duplicates and other unrelated documents that did not address the objective of the review. Policy documents meeting the inclusion criteria were labeled “yes” and those not meeting the inclusion criteria were labeled “no”. To avoid incorrectly excluding policy documents that met the inclusion criteria, we erred on the side of caution: when in doubt we reviewed the full text of policy documents and discussed it with some of the review team based in Kenya (MN, GEO, KO, PK-W, and MM). Any disputes were resolved after consultation with senior members of the team (PG, RH and HE) as well as a policy expert in Kenya (GEO).

#### Step 3—Data extraction and analysis

Eleven members of the review team (MA-O, MN, PK-W, KO, IC, MW, RM, LO, HE, MM, and EM) initially extracted policy details including title and year of publication, aims/goals, focus on center-based care, ECD and related terms, urban poor/ informal settlements, components of the NCF as detailed in Table 1. The eleven-member team met regularly to review the data extraction process, resolve challenges, and clarify any issues arising from the documents being extracted.

To comprehensively extract policy documents to reflect the NCF components addressed by each of the policy documents, a coding key was developed to match each detailed element listed under the five main NCF components (*good health, adequate nutrition, responsive caregiving, opportunities for early learning, and security and safety*) as detailed in Table 1, with each element listed under the NCF components labeled with the letters of the alphabets. Two tables were created to capture detailed data on each element listed under the NCF components (see Supplementary materials 1, 2), with a corresponding coding table for both national policies and CIDPs (see Tables 2, 3), that captured whether these elements were addressed in the respective policy documents. Coding keys were used to determine whether the elements listed under each NCF components have been fully addressed (++), partially addressed (+) or not addressed (x) in the policy documents included. This approach to coding was necessary as it helped to quickly identify all the gaps in the included policy documents with respect to the components of the NCF.

**Table 2:** National policy documents data coding table.

**Kenyan National Policies**	**1. Good Health:**	**2. Adequate nutrition:**	**3. Responsive caregiving:**	**4. Opportunities for early learning:**	**5. Security and safety:**
	A. Family planning B. Immunization for mothers and children C. Prevention and cessation of smoking, alcohol and substance use D. Prevention of mother-to-child transmission of HIV E. Support for caregivers' mental health F. Antenatal and childbirth care G. Prevention of preterm births H. Essential care for new-born babies, with extra care for small and sick babies I. Kangaroo care for low-birthweight babies J. Support for timely and appropriate care seeking for sick children K. Integrated management of childhood illness L. Early detection of disabling conditions (such as problems with sight and hearing) M. Care for children with developmental difficulties and disabilities	A. Maternal nutrition B. Support for early initiation, exclusive breastfeeding, and continued breastfeeding after 6 months C. Support for appropriate complementary feeding and for transition to a healthy family diet D. Micronutrient supplementation for mother and child, as needed E. Fortification of staple foods F. Growth monitoring and promotion, including intervention and referral when indicated G. Deworming H. Support for appropriate child feeding during illness I. Management of moderate and severe malnutrition as well as being overweight or obese	A. Skin-to-skin contact immediately after birth B. Kangaroo care for low-birthweight babies C. Rooming-in for mothers and young infants and feeding on demand D. Responsive feeding E. Interventions that encourage play and communication activities of caregiver with the child. F. Interventions to promote caregiver sensitivity and responsiveness to children's cues G. Support for caregivers' mental health. H. Involving fathers, extended family and other partners I. Social support from families, community groups and faith communities	A. Information, support and counseling about opportunities for early learning, including the use of common household objects and home-made toys B. Play, reading and story-telling groups for caregivers and children C. Book sharing D. Mobile toy and book libraries E. Good-quality day care for children, and pre-primary education F. Storytelling of elders with children G. Using local language in children's daily care	A. Birth registration B. Provision of safe water and sanitation C. Good hygiene practices at home, at work and in the community D. Prevention and reduction of indoor and outdoor air pollution E. Clean environments free of hazardous chemicals F. Safe family and play spaces in urban and rural areas G. Prevention of violence by intimate partners and in families, as well as services for addressing it H. Social care services I. Cash or in-kind transfers and social insurance J. Supporting family care and foster care over institutional care
The National Children's Policy (36)	A+ B+ C++ D+ Ex F+ Gx Hx Ix Jx K+ Lx M+	Ax B+ Cx D+ Ex Fx Gx Hx Ix	Ax Bx Cx Dx Ex Fx Gx Hx I++	N/A	A++ B+ C+ Dx Ex Fx Gx Hx Ix J++
The framework for the national child protection system for Kenya (37)	Ax B+ C+ Dx Ex Fx Gx Hx Ix J+ Kx Lx Mx	N/A	Ax Bx Cx Dx Ex Fx Gx Hx I++	Ax Bx Cx Dx E+ Fx Gx	Ax B+ Cx Dx Ex Fx G+ H+ Ix Jx
Kenya National Social Protection Policy (38)	N/A	N/A	N/A	N/A	Ax Bx Cx Dx Ex Fx Gx H+ I+ J+
National School Health Strategy Implementation Plan (39)	Ax Bx C+ Dx Ex Fx Gx Hx Ix J+ Kx Lx M+	N/A	N/A	N/A	Ax B+ C+ Dx Ex Fx Gx Hx Ix Jx
Laws of Kenya, Children Act, Chapter (40)	Ax B+ C+ Dx Ex Fx Gx Hx Ix Jx Kx Lx Mx	Ax Bx C+ Dx Ex Fx Gx Hx Ix	N/A	Ax B+ Cx Dx E+ Fx Gx	A++ Bx Cx Dx Ex Fx G+ Hx Ix Jx
Kenya Health Policy (41)	Ax Bx C+ Dx Ex Fx Gx Hx Ix Jx Kx Lx Mx	Ax B+ Cx D+ Ex Fx Gx Hx I+	N/A	N/A	Ax, Bx C+ Dx Ex Fx Gx, H+ Ix Jx
A national framework and plan of action for implementation of Integrated Community case Management in Kenya (42)	Ax B+ Cx Dx Ex Fx Gx Hx Ix Jx Kx Lx Mx	Ax B+ C+ Dx Ex Fx Gx Hx Ix	A+ Bx Cx Dx Ex Fx Gx Hx Ix	N/A	A++ B+ C+ Dx Ex Fx Gx Hx Ix Jx
National Standards for Best Practice in Charitable Children's Institutions (43)	Ax B+ Cx Dx Ex Fx Gx Hx Ix Jx Kx L+ M+	Ax Bx Cx Dx Ex F+ Gx Hx Ix	Ax Bx Cx Dx Ex Fx Gx H+ Ix	A+ B+ Cx Dx E+ Fx Gx	Ax B+ C+ Dx Ex Fx Gx Hx Ix Jx
National Maternal, Infant and Young Child Nutrition Policy Guidelines (44)	A+ B+ Cx D++ Ex F+ G+ H+ I++ J+ Kx L+ M+	A++ B+ C++ D++ Ex F+ Gx H+ I+	A++ B++ C++ D++ Ex Fx Gx H+ I++	N/A	Ax B+ Cx Dx Ex Fx Gx Hx Ix Jx
The National Plan of Kenya against sexual exploitation of children (45)	N/A	N/A	Ax Bx Cx Dx Ex Fx Gx H+ I+	N/A	Ax Bx Cx Dx Ex Fx G+ Hx Ix
Kenya National Nutrition Action Plan (46)	N/A	A+ B+ Cx D+ E+ F+ Gx Hx I+	N/A	N/A	Ax B+ Cx Dx Ex Fx Gx Hx Ix Jx
Policy guidelines for management of diahorrea in children below 5 years in Kenya (47)	N/A	Ax B+ Cx D+ Ex Fx Gx H++ I+	Ax Bx Cx Dx Ex Fx Gx Hx I+	N/A	Ax B+ C+ Dx Ex Fx Gx Hx Ix Jx
Guidelines for the Alternative Family Care of Children in Kenya (48)	Ax Bx Cx Dx Ex Fx Gx Hx Ix J+ Kx Lx M+	N/A	N/A	N/A	Ax Bx Cx Dx Ex Fx Gx Hx I+ J+
National Plan of Action for Children in Kenya (49)	N/A	A+ B+ C+ D+ E+ Fx Gx Hx I+	N/A	N/A	Ax B+ C+ Dx Ex Fx Gx H+ Ix Jx
Kenya Reproductive, Maternal, Newborn, Child and Adolescent Health Investment Framework (50)	A++ Bx Cx Dx Ex Fx Gx Hx Ix Jx K+ Lx Mx	Ax B++ C+ D++ Ex Fx G+ Hx Ix	N/A	N/A	A+ Bx Cx Dx Ex Fx Gx Hx Ix Jx
Footprints Children's Home Child Protection Policy (51)	Ax Bx C+ Dx Ex F+ Gx Hx Ix Jx K+ Lx M+	N/A	N/A	N/A	A++ B+ Cx Dx Ex Fx G+ Hx Ix J+
Sector Policy for Learners and Trainees with Disabilities (52)	Ax, Bx Cx Dx Ex Fx Gx Hx Ix Jx Kx Jx L+ M+	N/A	N/A	N/A	Ax, B+ Cx Dx Ex Fx Gx Hx Ix Jx
National Pre-Primary Education Policy Standard Guidelines (53)	Ax B+ Cx Dx Ex Fx Gx Hx Ix Jx K+ L+ M++	Ax Bx Cx D+ Ex F+ G+ Hx Ix	Ax Bx Cx Dx Ex Fx Gx H+ I+	A+ B+ Cx Dx E++ Fx Gx	Ax B+ C+ Dx Ex F+ G++ Hx Ix Jx
Kenya Community Health Policy (54)	A+ B+ C+ Dx E+ F++ Gx H+ Ix J+ Kx L+ M+	A+ B++ Cx D+ E+ F+ Gx Hx I+	Ax Bx Cx D+ E+ Fx Gx Hx I+	A+ Bx Cx Dx Ex Fx Gx	Ax B+ C+ Dx E+ Fx Gx Hx Ix J+

**++**, Mentioned and provided some details about plans or implementation.

**+**, Just mentioned with no details on plans or implementation).

x, Not mentioned at all.

**Table 3:** Data coding for the components of the nurturing care framework as addressed in the Kenyan CIDPs.

**Kenya's 47 County Integrated Development Plans (CIDPs) from 2018-2022/3**	**Good Health:**	**Adequate nutrition:**	**Responsive caregiving:**	**Opportunities for early learning:**	**Security and safety:**
	A. Family planning B. Immunization for mothers and children C. Prevention and cessation of smoking, alcohol and substance use D. Prevention of mother-to-child transmission of HIV E. Support for caregivers' mental health F. Antenatal and childbirth care G. Prevention of preterm births H. Essential care for new-born babies, with extra care for small and sick babies I. Kangaroo care for low-birthweight babies J. Support for timely and appropriate care seeking for sick children K. Integrated management of childhood illness L. Early detection of disabling conditions (such as problems with sight and hearing) M. Care for children with developmental difficulties and disabilities	A. Maternal nutrition B. Support for early initiation, exclusive breastfeeding and continued breastfeeding after 6 months C. Support for appropriate complementary feeding and for transition to a healthy family diet D. Micronutrient supplementation for mother and child, as needed E. Fortification of staple foods F. Growth monitoring and promotion, including intervention and referral when indicated G. Deworming H. Support for appropriate child feeding during illness I. Management of moderate and severe malnutrition as well as being overweight or obese	A. Skin-to-skin contact immediately after birth B. Kangaroo care for low-birthweight babies C. Rooming-in for mothers and young infants and feeding on demand D. Responsive feeding E. Interventions that encourage play and communication activities of caregiver with the child. F. Interventions to promote caregiver sensitivity and responsiveness to children's cues G. Support for caregivers' mental health H. Involving fathers, extended family and other partners I. Social support from families, community groups and faith communities	A. Information, support and counseling about opportunities for early learning, including the use of common household objects and home-made toys B. Play, reading and story-telling groups for caregivers and children C. Book sharing D. Mobile toy and book libraries E. Good-quality day care for children, and pre-primary education F. Storytelling of elders with children G. Using local language in children's daily care	A. Birth registration B. Provision of safe water and sanitation C. Good hygiene practices at home, at work and in the community D. Prevention and reduction of indoor and outdoor air pollution E. Clean environments free of hazardous chemicals F. Safe family and play spaces in urban and rural areas G. Prevention of violence by intimate partners and in families, as well as services for addressing it H. Social care services I. Cash or in-kind transfers and social insurance J. Supporting family care and foster care over institutional care
Baringo (55)	A**+** B**+** Cx D+ Ex F++ Gx Hx Ix Jx Kx Lx Mx	Ax B+ C+ D+ Ex F+ G+ Hx I++	Ax Bx Cx D+ E+ F+ Gx Hx Ix	A+ B+ Cx D+ E**+** F**x** G**x**	A++ B++ C+ D++ E+ F+ G+ H+ I++ Jx
Bomet (56)	A**++** B+ Cx D+ Ex F++ Gx Hx Ix Jx Kx Lx Mx	A+ B+ Cx D+ Ex F+ G+ Hx I+	Ax Bx Cx D+ E+ F+ Gx Hx Ix	A+ Bx Cx D+ E**++** F**x** G**x**	Ax B++ C+ D+ E+ Fx Gx H+ I+ Jx
Bungoma (57)	A**++** B+ C+ Dx Ex F++ Gx H+ Ix Jx Kx Lx Mx	Ax B+ C+ D++ Ex F+ G+ Hx Ix	Ax Bx Cx D+ E+ F+ Gx Hx Ix	A+ B+ Cx D+ E**++** F**x** G**x**	Ax B++ C+ D+ E+ F+ G+ H++ I+ Jx
Busia (58)	A++ B+ C+ D++ Ex F++ Gx Hx Ix Jx Kx Lx Mx	Ax B+ C+ D++ Ex F+ G+ Hx I++	Ax Bx Cx D++ E+ F+ Gx Hx Ix	A+ B+ Cx D+ E++ Fx Gx	Ax B++ C+ D+ E+ Fx G+ H+ I++ Jx
Elgeyo/Marakwet (59)	A++ B+ C+ D++ Ex F++ Gx H+ Ix Jx Kx Lx Mx	A+ B+ C+ D++ Ex F++ G+ Hx I+	Ax Bx Cx D+ E+ F+ Gx Hx Ix	A+ Bx Cx D+ E++ Fx Gx	Ax B++ C+ D+ E++ Fx G+ H+ I+ Jx
Embu (60)	A++ B+ C+ D++ E+ F++ Gx Hx Ix Jx Kx Lx Mx	A+ Bx Cx D+ Ex F++ Gx Hx I++	Ax Bx Cx D+ E+ F+ G+ Hx Ix	A+ B+ Cx D+ E++ Fx Gx	Ax B++ C+ D+ E+ F+ G+ H+ I+ Jx
Garissa (61)	A++ B+ C+ D++ Ex F++ Gx Hx Ix Jx K+ Lx Mx	A+ Bx Cx D+ Ex F++ Gx Hx I++	Ax Bx Cx D+ E++ F++ Gx Hx Ix	A+ B+ Cx D+ E++ Fx Gx	A++ B++ C+ D++ E+ F++ G+ H+ I++ Jx
Homa Bay (62)	A++ B+ Cx D+ Ex Fx Gx H++ Ix J+ Kx Lx Mx	Ax B++ C+ D+ Ex F+ G++ Hx Ix	Ax Bx Cx D+ E+ F+ Gx Hx Ix	A+ B+ Cx D+ E++ Fx Gx	Ax B++ C+ D+ E+ Fx G+ H+ I+ Jx
Isiolo (63)	A++ B+ Cx D++ Ex F++ Gx Hx Ix Jx Kx Lx Mx	Ax B++ C+ D++ E+ F+ G++ Hx Ix	Ax Bx Cx D++ E+ F+ Gx Hx I+	A+ B+ Cx D+ E++ Fx Gx	A++ B++ C+ Dx E+ F+ G+ H++ I+ Jx
Kajiado (64)	A++ B+ C+ Dx Ex F++ Gx Hx Ix Jx K+ Lx Mx	A+ B+ Cx D+ Ex F++ G+ Hx I++	Ax Bx Cx D+ E+ Fx Gx Hx Ix	A+ Bx Cx D+ E++ Fx Gx	Ax B+ C+ D+ E+ Fx G+ H++ I++ Jx
Kakamega (65)	A++ B+ C+ D+ Ex F++ G+ Hx Ix Jx Kx Lx Mx	Ax B+ Cx D+ Ex F+ G+ Hx I+	Ax Bx Cx D+ E+ F+ Gx Hx Ix	A+ B+ Cx D+ E++ Fx Gx	Ax B++ C++ D+ E+ Fx G+ H++ I++ Jx
Kericho (66)	A++ B+ Cx D++ Ex F++ Gx Hx Ix Jx Kx Lx Mx	A+ Bx Cx D+ Ex F+ G+ Hx I+	Ax Bx Cx D+ E+ F+ Gx Hx Ix	A+ Bx Cx D+ E++ Fx Gx	Ax B++ C+ D+ E+ Fx G+ H++ I++ Jx
Kiambu (67)	A++ B+ C+ D++ Ex F++ Gx Hx Ix Jx Kx LxM+	Ax B+ C+ D+ Ex F++ G+ Hx I+	Ax Bx Cx D++ E+ F+ Gx Hx Ix	A+ B+ Cx D+ E+ Fx Gx	Ax B++ C++ D+ E++ F+ G+ H+ I+ Jx
Kilifi (68)	A++ B+ C+ Dx Ex F+ Gx Hx Ix Jx Kx Lx Mx	A+ Bx C+ D+ Ex F+ Gx Hx I+	Ax Bx Cx D+ E+ Fx Gx Hx Ix	A+ B+ Cx D+ E+ Fx Gx	A++ B+ C+ D+ E+ Fx G+ H++ I+ Jx
Kirinyaga (69)	A++ B+ C++ D++ Ex F++ Gx Hx Ix Jx Kx Lx Mx	A+ B+ C+ Dx Ex F+ Gx Hx I+	Ax Bx Cx D+ E+ Fx Gx Hx Ix	A+ B+ Cx D+ E+ Fx Gx	Ax B+ C+ D+ E+ Fx G+ H+ I+ Jx
Kisii (70)	A++ B+ Cx D++ Ex F+ Gx H+ Ix Jx Kx Lx Mx	A+ Bx Cx D++ Ex F+ G+ Hx I+	Ax Bx Cx D+ E+ Fx Gx Hx Ix	A+ B+ C+ D+ E++ Fx Gx	Ax B++ C+ D+ E+ Fx Gx H+ I+ Jx
Kitui (71)	A++ B+ C+ D++ Ex F++ Gx Hx Ix Jx Kx Lx Mx	A+ B++ C+ D+ E+ F+ G++ Hx I+	Ax Bx Cx D++ Ex Fx Gx Hx Ix	A+ Bx Cx D+ E++ Fx Gx	Ax B+ C+ D+ E+ Fx G+ H+ I+ Jx
Kisumu (72)	Ax B+ C+ Dx Ex F+ Gx Hx Ix Jx Kx Lx Mx	A+ B+ Cx D+ Ex F+ G+ Hx I+	Ax Bx Cx D++ E+ Fx Gx Hx Ix	A+ B+ Cx D+ E++ Fx Gx	Ax B++ C+ D+ E+ Fx G+ H+ I++ Jx
Kwale (73)	A++ B+ Cx D++ Ex F++ Gx Hx Ix Jx Kx Lx Mx	Ax Bx C+ Dx Ex F+ Gx Hx I+	Ax Bx Cx D+ E+ Fx Gx Hx Ix	A+ Bx Cx D+ E+ Fx Gx	Ax B+ Cx Dx E+ Fx G+ Hx I+ Jx
Laikipia (74)	A++ B+ C+ D+ Ex F++ Gx Hx Ix Jx Kx Lx Mx	A+ Bx Cx D+ Ex F++ Gx H+ Ix	Ax Bx Cx D+ Ex F+ Gx Hx Ix	A++ Bx Cx D+ E+ Fx Gx	Ax B++ Cx D+ Ex Fx Gx H+ I+ Jx
Lamu (75)	A++ B+ Cx D+ Ex F++ Gx Hx Ix Jx Kx Lx Mx	Ax Bx C+ Dx Ex F+ Gx Hx Ix	Ax Bx Cx D+ E+ F+ Gx Hx Ix	A+ B+ C+ D+ E+ Fx Gx	Ax B++ Cx D+ Ex Fx Gx H+ I+ Jx
Machakos (76)	A++ B+ C+ D++ Ex F++ Gx Hx Ix Jx Kx Lx Mx	Ax B+ Cx D+ Ex F++ Gx Hx I+	Ax Bx Cx D+ E+ Fx Gx Hx Ix	A+ Bx Cx D+ E+ Fx Gx	Ax B++ C+ D+ E+ F++ G+ H+ I+ Jx
Mandera (77)	A++ B+ C+ D++ Ex F++ Gx Hx Ix Jx Kx Lx Mx	Ax Bx Cx Dx Ex F++ G+ Hx I+	Ax Bx Cx D+ E++ F++ Gx Hx Ix	A++ B+ Cx D+ E++ Fx Gx	Ax B++ C+ D+ E+ F+ G+ H+ I+ Jx
Makueni (78)	A++ B+ C+ Dx Ex F++ Gx Hx Ix Jx Kx Lx Mx	Ax Bx Cx Dx Ex Fx G+ Hx Ix	Ax Bx Cx D+ Ex F+ Gx Hx Ix	A+ Bx Cx D+ E++ Fx Gx	Ax B++ C+ Dx E+ Fx G+ Hx I+ Jx
Marsabit (79)	A+ B+ C+ D+ Ex F+ Gx Hx Ix Jx Kx Lx Mx	A+ Bx Cx Dx Ex F+ Gx Hx I+	Ax Bx Cx D+ E++ F+ Gx Hx Ix	A++ B+ Cx D+ E++ Fx Gx	Ax B++ C+ D+ E+ Fx G+ H+ I+ Jx
Meru (80)	A++ B+ C+ D+ Ex F++ Gx Hx Ix Jx Kx Lx Mx	Ax Bx C+ D+ E+ F+ G+ Hx Ix	Ax Bx Cx D+ E+ F+ Gx Hx Ix	A+ Bx Cx D+ E+ Fx Gx	Ax B++ C+ D+ E+ Fx G+ Hx Ix Jx
Migori (81)	A++ B+ C+ D++ Ex F++ Gx Hx Ix Jx Kx Lx Mx	Ax B+ Cx D+ Ex F+ G++ Hx Ix	Ax Bx Cx D+ E+ F+ Gx Hx Ix	A+ Bx Cx D+ E++ Fx Gx	Ax B+ C+ D+ E+ Fx G+ H+ I+ Jx
Mombasa (82)	A++ B+ Cx Dx Ex F++ Gx Hx Ix Jx Kx Lx Mx	Ax Bx C+ Dx Ex F+ G+ Hx Ix	Ax Bx Cx D+ E+ F+ Gx Hx Ix	A+ B+ Cx D+ E++ Fx Gx	Ax B+ C+ D+ E+ Fx G+ H+ I+ Jx
Muranga (83)	A++ B+ C+ D+ Ex F++ Gx Hx Ix Jx Kx Lx Mx	Ax Bx C+ D+ Ex F+ G+ Hx I+	Ax Bx Cx D+ Ex F+ Gx Hx Ix	A+ Bx Cx D++ E+ Fx Gx	Ax B+ C+ D+ E+ F+ Gx H+ I+ Jx
Nairobi (84)	A++ B+ Cx Dx Ex F++ Gx Hx Ix Jx Kx Lx Mx	Ax B+ Cx Dx E+ F+ G++ Hx Ix	Ax Bx Cx D+ E+ F+ Gx Hx Ix	A+ Bx Cx D+ E++ Fx Gx	Ax B+ C+ D+ E+ F+ G+ H+ I+ Jx
Nakuru (85)	A++ B+ C+ D++ Ex F++ Gx Hx Ix Jx Kx Lx Mx	Ax B+ Cx D+ Ex F+ G+ Hx I+	Ax Bx Cx D+ E+ F+ Gx Hx Ix	A+ B+ Cx D+ E+ Fx Gx	Ax B++ C+ D+ E+ Fx G+ H+ I+ Jx
Nandi (86)	A+ B+ C++ Dx Ex F++ Gx H+ I+ Jx Kx Lx Mx	Ax B+ Cx D++ Ex F+ Gx Hx Ix	Ax B+ Cx D++ E+ F+ Gx Hx Ix	A+ B+ Cx D+ E++ Fx Gx	Ax B++ C++ D+ E+ F+ G+ H+ I+ Jx
Narok (87)	A++ B+ Cx D++ Ex F++ G+ Hx Ix Jx Kx Lx Mx	A+ Bx Cx Dx Ex F+ G+ Hx I+	Ax Bx Cx D+ E+ F++ Gx Hx Ix	A+ Bx Cx D+ E+ Fx Gx	A++ B++ C++ D++ E++ F+ G+ H++ I++ Jx
Nyamira (88)	A++ B++ C+ D+ Ex F++ Gx H+ Ix Jx Kx Lx Mx	A+ B++ C++ D+ Ex F+ Gx Hx Ix	Ax Bx Cx D++ E+ F+ Gx Hx Ix	A++ Bx Cx D+ E+ Fx Gx	Ax B++ C++ D+ Ex F+ G+ H++ I+ Jx
Nyandarua (89)	A++ B+ C+ D++ Ex F++ Gx Hx Ix Jx Kx Lx Mx	A+ B+ C+ D++ Ex F+ Gx Hx Ix	Ax Bx Cx D+ E+ F++ Gx Hx Ix	A+ B+ Cx D+ E++ Fx Gx	Ax B++ C++ D+ E+ F+ Gx H+ I+ Jx
Nyeri (90)	A+ B+ C+ Dx Ex F++ Gx Hx Ix Jx Kx Lx Mx	Ax Bx Cx Dx Ex F+ G+ Hx I+	Ax Bx Cx D+ E+ Fx Gx Hx Ix	A+ B+ Cx D+ E++ Fx Gx	Ax B++ Cx D+ E+ F+ Gx H+ I++ Jx
Siaya (91)	A++ B+ C+ D+ Ex F++ Gx Hx Ix Jx Kx Lx Mx	A+ B+ C++ D+ Ex F+ G++ Hx Ix	Ax Bx Cx D++ E+ F+ Gx Hx Ix	A+ Bx Cx D+ E++ Fx Gx	Ax B++ C+ D+ E+ Fx Gx H+ Ix Jx
Samburu (92)	A+ B+ C+ D++ Ex F++ Gx Hx Ix Jx Kx Lx Mx	A+ B+ Cx D++ Ex F++ G+ Hx Ix	Ax Bx Cx D++ E++ F+ Gx Hx Ix	A+ B+ Cx D+ E++ Fx Gx	A++ B++ C+ D+ Ex Fx G+ H++ I++ Jx
Taita Taveta (93)	A++ B+ C+ D+ Ex F++ Gx Hx I+ Jx K+ Lx Mx	A+ B+ Cx D+ Ex F++ Gx Hx Ix	Ax B+ Cx D+ E+ F++ Gx Hx Ix	A+ Bx Cx D+ E++ Fx Gx	Ax B++ C+ D+ E+ F+ G+ H+ I++ Jx
Tana River (94)	A++ B+ C+ D++ Ex F++ Gx H+ I+ Jx K++ Lx Mx	A+ B+ C++ D++ E+ F+ G+ Hx Ix	Ax B+ Cx D++ E+ F+ Gx Hx Ix	A+ Bx Cx D+ E++ Fx Gx	A++ B++ C+ D+ E+ F+ G+ H+ I+ Jx
Tharaka-Nithi (95)	A++ B+ C+ Dx Ex F++ Gx Hx Ix Jx Kx Lx Mx	Ax Bx Cx D+ Ex Fx G+ Hx Ix	Ax Bx Cx D+ E++ F+ Gx Hx Ix	A+ Bx Cx D+ E++ Fx Gx	A++ B++ C+ D+ E+ Fx G+ H+ I+ Jx
Trans Nzoia (96)	A++ B+ C+ D++ E+ F++ Gx H+ Ix Jx K+ L+ Mx	A+ Bx Cx Dx Ex F++ G+ Hx I+	Ax Bx Cx D+ E++ F+ G+ Hx Ix	A+ B+ Cx D+ E++ Fx Gx	Ax B++ C+ D+ E+ Fx G+ H+ I++ Jx
Turkana (97)	A++ B+ C++ D++ Ex F+ Gx Hx Ix Jx Kx Lx Mx	A+ B+ C+ Dx Ex F+ G+ Hx I+	Ax Bx Cx D+ E+ F++ Gx Hx Ix	A+ Bx Cx D+ E++ Fx Gx	Ax B++ C+ D+ E+ Fx G+ H+ I++ Jx
Uasin Gishu (98)	A++ B+ C+ D++ Ex F++ Gx Hx Ix Jx Kx Lx Mx	Ax Bx Cx D+ Ex Fx G+ Hx I+	Ax Bx Cx D+ E+ F+ Gx Hx Ix	A+ Bx Cx D+ E++ Fx Gx	Ax B++ C+ D+ E+ Fx G+ H+ Ix Jx
Vihiga (99)	A++ B+ C+ D++ Ex F++ Gx Hx Ix Jx Kx Lx Mx	Ax B+ C+ D+ Ex F+ Gx Hx Ix	Ax Bx Cx D+ E+ F+ Gx Hx Ix	A+ Bx Cx D+ E+ Fx Gx	Ax B++ Cx D+ Ex F+ G+ H+ I++ Jx
Wajir (100)	A++ B+ Cx D++ Ex F++ Gx Hx Ix Jx K+ Lx Mx	Ax B+ C+ D+ E+ F+ G++ Hx Ix	Ax Bx Cx D+ E+ F+ Gx Hx Ix	A+ B+ Cx D+ E++ Fx Gx	A++ B++ C+ D+ E+ F+ G+ H+ I+ Jx
West Pokot (101)	A++ B+ Cx D+ Ex F++ Gx Hx Ix Jx Kx Lx Mx	Ax Bx C+ D++ Ex F+ G+ Hx Ix	Ax Bx Cx D+ E+ F+ Gx Hx Ix	A+ B+ Cx D+ E++ Fx Gx	A++ B++ C+ D+ E+ F+ G+ H+ I++ Jx

++, Mentioned and provided some details about plans or implementation.

+, Just mentioned with no details on plans or implementation).

x, Not mentioned at all.

### Policy/decision-maker interviews

We conducted individual qualitative interviews with policy and decision-makers at national, Nairobi County and sub-County (Makadara and Ruaraka) levels to understand the policy and planning context, the extent to which the components of the NCF have been addressed within policies and plans and any perceived gaps or challenges to their inclusion in future. Nairobi County was the only county involved in the qualitative interviews as this policy review has been nested within a larger feasibility study being conducted in Nairobi (35). This interview study has been approved by Amref Health Africa's Ethics and Scientific Review Committee (Ref: P7802020) and the University of York Health Sciences Research Governance Committee (Ref: HSRGC). All participants provided their written informed consent to participate in this study including to audio-record interviews. Interviews were conducted in English.

Participants were selected using purposive sampling including national policy makes and county-level decision-makers of different levels of seniority within departments that focus on child health, child protection, development, and early years education. Both telephone and face-to-face interviews were utilized based on participant's availability, prevailing COVID-19 restrictions, and convenience. All the face-to-face interviews were conducted at the participants' workplace and the telephone interviews were held at a time convenient to the participant. A semi-structured interview guide was used to facilitate the interviews and included views on ECD and its perceived importance to government policy, details of any policies, plans or guidance at national and county level relating to child health and ECD and the extent they aligned to the NCF domains, the age groups addressed and any specific focus on childcare centers for vulnerable communities. The questions were open-ended and allowed flexibility to focus on areas of relevance to the participants. The interviews were conducted by LO, PA, MN, KO and PW, all of whom had experience in qualitative methods. Before the interviews, the researchers introduced themselves to the participants, explaining the objectives of the study.

An initial sample size of *n* = 20 participants were anticipated and interviews continued until data saturation was achieved (thus as interviews progressed, emerging themes were discussed among interviewers periodically and agreement was reached on the sufficiency of data collected to answer the objectives set for the interviews). The audio recorded interviews were transcribed soon after the interviews, anonymized and combined with the notes taken by the interviewers during the interview sessions. Due to time constraints, the transcripts were not returned to the participant for comments and/or corrections. However, findings were shared at engagement events with policy and decision makers.

### Qualitative analysis

The analysis process followed the stages outlined in the Framework Approach (102) with the NCF used as the main framework to guide analysis. Given the multiple interviewers, the initial analysis process was conducted as a group activity with team members coding transcripts and identifying common emerging issues. These discussions informed the subthemes emerging within each of the NCF domains. Additional themes on the challenges, successes and future plans in policy development and implementation in relation to the NCF were included within the framework. The final framework was used within NVivo 2020 where transcripts and notes were coded and grouped into the themes by authors PA and HE. Following coding of the first three transcripts, we checked consistency between the two coders and agreed how to resolve any differences. Summaries were written under each theme highlighting similarities and differences in respondents' views across sector and seniority level. To further understand the challenges, successes and future plans of policy and implementation under each of the NCF domains, the Jaccards' similarity coefficient (103) function in NVivo 2020 was used to assess similarities between the themes being compared (where 0 = least similar, 1 = most similar). The coefficient shows the similarity index between the themes. Jaccards' coefficient only counts the true positives (similar coding) in the groups being compared. This quantification of the relationships allowed us to further explore the underlying reasons for coverage of each of NCF domains by different policy and decision makers at different levels.

## Results

### Characteristics of policy documents identified

**Figure 1:**
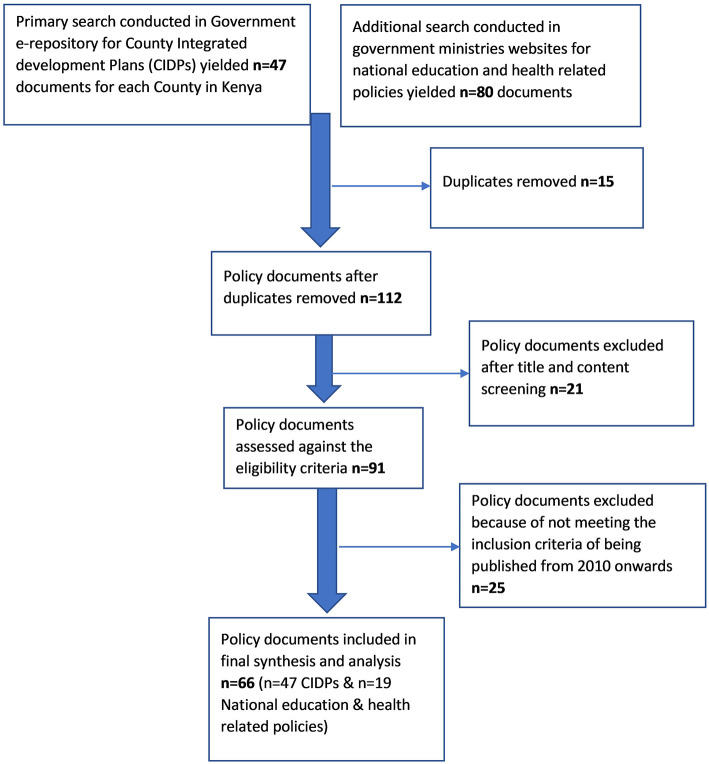
Policy documents selection process.

A total of 127 policy documents were identified and retrieved from the government e-repository and county websites. Of these 127 documents, *n* = 15 duplicates were removed, *n* = 21 were excluded after title screening and *n* = 91 were assessed against the inclusion and exclusion criteria. More than half (*n* = 66) met the inclusion criteria and *n* = 25 was excluded (see Figure 1). The 66 documents included 19 national policy documents that covered single ministries (health, education, labor, and social and child protection among others) and 47 CIDPs, see Tables 2, 3.

### Decision-maker interview participant characteristics

Twenty key decision-maker interviews were conducted with various personnel at the national level and within Nairobi County as well as sub-County governments. Of the 20 interviews conducted, 11 were by telephone and nine were face to face interviews. Characteristics of the qualitative participants are given in Table 4.

**Table 4:** Qualitative interview participant characteristics.

**Respondent**	**Sex**	**Seniority**	**Sector**	**Level**
P1 (COP/PM/001)	Male	Junior official	Health	Sub-county
P2 (COP/PM/002)	Female	Senior official	Health	National
P3 (COP/PM/003)	Male	Junior official	Education	National
P4 (COP/PM/004)	Female	Junior official	Health	Sub-county
P5 (COP/PM/005)	Female	Junior official	Education	National
P6 (COP/PM/006)	Female	Junior official	Health	Sub-county
P7 (COP/PM/007)	Female	Senior official	Health	Sub-county
P8 (COP/PM/008)	Male	Senior official	Health	County
P9 (COP/PM/009)	Male	Senior official	Education	National
P10 (COP/PM/010)	Female	Senior official	Health	Sub-county
P11 (COP/PM/011)	Female	Junior official	Education	County
P12 (COP/PM/012)	Male	Senior official	Education	County
P13 (COP/PM/013)	Female	Senior official	Health	County
P14 (COP/PM/014)	Male	Senior official	Health	Sub-county
P15 (COP/PM/015)	Male	Junior official	Health	Sub-county
P16 (COP/PM/016)	Female	Junior official	Health	Sub-county
P17 (COP/PM/017)	Female	Senior official	Health	Sub-county
P18 (COP/PM/018)	Female	Junior official	Health	Sub-county
P19 (COP/PM/019)	Male	Junior official	Health	Sub-county
P20 (COP/PM/020)	Female	Junior official	Health	Sub-county

### Findings from policy data synthesis and policy/decision maker's reflections

National policies and CIDPs data synthesized mainly focused on information captured on children under 5 years however, majority of the CIDPs addressed age groups ranging from 0 to 5 years, 3 to 5, and 4 to 5 year olds (see Supplementary Tables 1, 2). Most plans in the CIDPs are targeted at pre-primary education, which mainly captured information on children from age 3–5 and sometimes 4–5 year olds. The national policies also have specific age ranges within their focus (e.g., the National Pre-Primary Education Policy addressed 4–5 year olds). These findings highlight the gaps in addressing the needs of children age 0–3 years in both national policies and CIDPs. Although the CIDPs captured information on almost all the components of the NCF, national policies predating 2018 had the most gaps in terms of the NCF components, see Table 2. Details regarding the coverage on the various components of the NCF have been discussed in turn below.

#### Coverage on “good health” components in national policies and CIDPs

**Figure 2:**
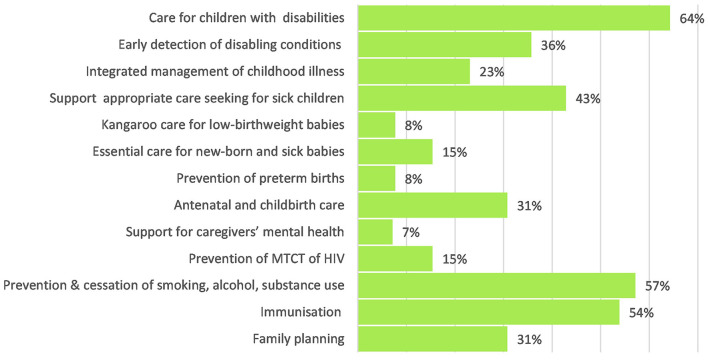
Coverage of “Good Health” within national policies.

National policies had a strong focus on breastfeeding, micronutrient supplements and management of malnutrition see Figure 2. Emphasis on care for children with disabilities, immunisations and prevention of substance abuse was also relatively strong. Care for new-borns, deworming, prevention of mother to child transmission (PMTCT) of HIV and critically, support for caregivers' mental health were not strongly addressed within national policies.

**Figure 3:**
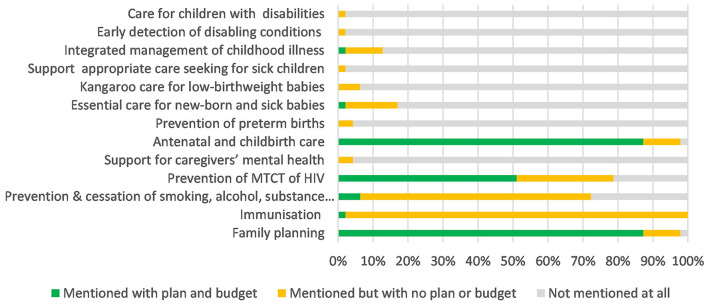
Coverage of “Good Health” within county integrated development plans.

Several areas that were addressed well in national policies received limited mention or budget allocation in the CIDPs see Figure 3. This was particularly evident in the “good health” domain where the early detection of and care for children with disabilities received relatively good coverage in national policies (64%) but was only mentioned in 2% of CIDPs and with no budget allocated.

#### Coverage on “adequate nutrition” components in national policies and CIDPs

**Figure 4:**
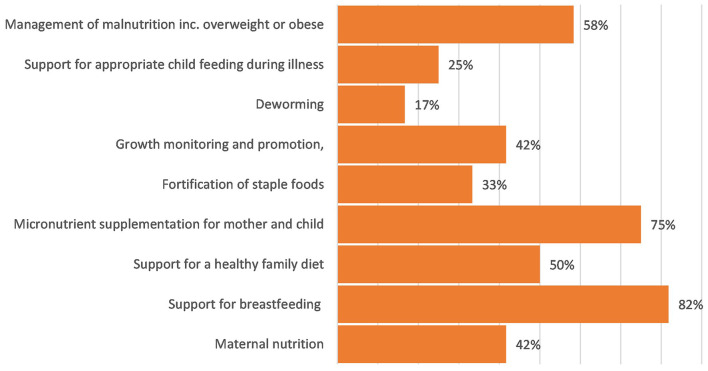
Coverage of “Adequate Nutrition” within national policy.

**Figure 5:**
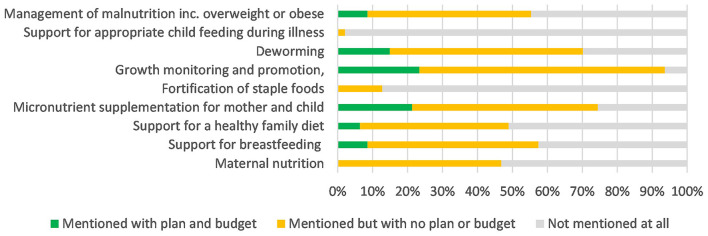
Coverage of “Adequate Nutrition” within county integrated development plans.

Under the “adequate nutrition” domain, growth monitoring received the most focus, with 70% of the CIDPS mentioning this; however, there was limited attention given, particularly within budgets to the other aspects of this domain, see Figures 4, 5.

#### Policy and decision-maker reflections on health and nutrition

The interviews with policy/decision-makers at both national and county level showed a high level of commitment to under five child health and nutrition. Consistently, respondents were able to identify and describe with the relevant policies in this area. The analysis of Jaccard's similarity coefficient confirmed that a high proportion of the text relating to nutrition and health was coded to “adequately addressed in policies and guidelines” (Jaccard's coefficient of over 0.5 for both nutrition and health, see Table 5). This was particularly the case for the county government officials who are responsible for implementation of policies. The health of children in the early years (0–3 years) was identified as being well-addressed in the policies, however with challenges in implementation, particularly beyond the health facility:

“*You will find that 0-3 is mostly health. Okay that's where we talk about in fact, we talk about bringing the child for clinics, Vitamin A supplementation, nutritional assessments. So, that aspect [health and nutrition] is well covered as long as the child is being brought to the facility. But more often than what we find after they finish their last vaccine at 18 months which is the second measles, a lot of caregivers don't bring the children for wellness check-ups.” (COP/PM/002 Ministry of Health, National Government)*.

**Table 5:** Relationship between NCF components and policy-maker perceptions of policy gaps.

**Component of NCF**	**Participants' responses**	**Jaccards' coefficient**
Good health	Well-covered in policy and guidelines	0.71
Good health	Adequate implementation	0.63
Good health	Plans for future policies exist	0.63
Adequate nutrition	Well-covered in policy and guidelines	0.75
Adequate nutrition	Adequate implementation	0.75
Adequate nutrition	Plans for future policies exist	0.63
Responsive caregiving	Well-covered in policy and guidelines	0.38
Responsive caregiving	Adequate implementation	0.38
Responsive caregiving	Plans for future policies exist	0.25
Opportunities for early learning	Well-covered in policy and guidelines	0.50
Opportunities for early learning	Adequate implementation	0.50
Opportunities for early learning	Plans for future policies exist	0.38
Safety and security	Addressed gaps on policies made and guidelines	0.75
Safety and security	Addressed gaps on policy implementation	0.75
Safety and security	Plans for future policies	0.63

The green colour indicates gaps and future plans of the components of the NCF addressed in both national policies and CIDPs with coefficients >0.5, The yellow colour indicates gaps and future plans of the components of the NCF addressed in both national policies and CIDPs with coefficients ≤ 0.5).

#### Coverage on responsive caregiving components in national policies and CIDPs

**Figure 6:**
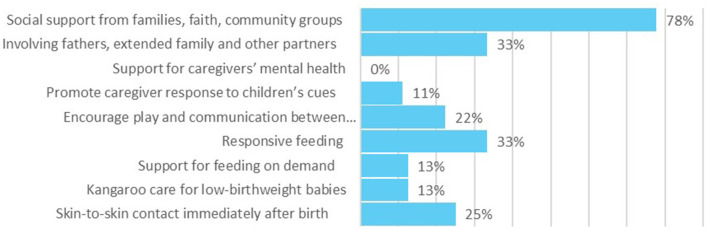
Coverage of “Responsive Caregiving” within national policies.

**Figure 7:**
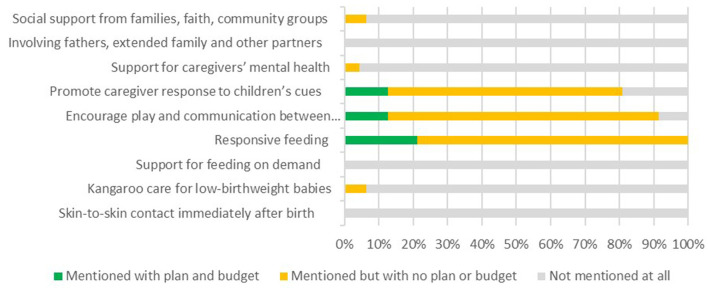
Coverage of “Responsive Caregiving” within county integrated development plans.

Responsive caregiving received limited coverage within national policies and CIDPs, see Figures 6, 7. While social support from families, involving fathers and families received relatively high emphasis in national level policies, these areas were barely mentioned in the CIDPs, and had no budget allocations across the 47 counties. The only involvement of fathers mentioned in the qualitative interviews was in relation to the issuing of birth certificates, and not as a key aspect of responsive care giving.

#### Policy/decision-maker reflections on responsive caregiving components

Responsive feeding was one area that did have a budget allocation in 21% of the CIDPs. This reflects the general trend for a stronger focus on health and nutrition than early childhood development particularly for the youngest children. Several policy makers highlighted the challenges of addressing areas such as responsive care and other aspects of early childhood development as the issues often fell between sector ministries:

“*I must agree we have had a challenge before because we really did not bring out the ECD the way it's supposed to be. You remember this thing cuts across two ministries. That's where the challenge is: there is the Ministry of Education, then there is the Ministry of Health. Education will say our work is to take care of these children in school from PP1 [pre-primary 1].The Ministry of Health will just say we take care of them when they are at home. Under whose supervision are these centers [childcare centers for 3 years and below], we are not sure whether they are under the Ministry of Health or under the Ministry of Education and that's why we must have a policy in place.” (COP/PM/008 Ministry of Health, County government)*.

National and County level decision-makers highlighted that the challenge of working across sectors was also experienced at County level:

“*The gap which is there is the collaboration. This is lacking because they devolved the health and they devolved ECD” (Policy maker COP/PM/005 Ministry of Education, National)*.

#### Coverage on “opportunities for early learning” components in national policies and CIDPs

**Figure 8:**
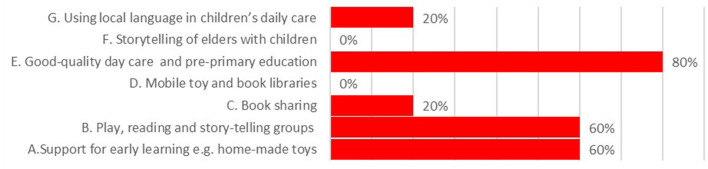
Coverage of “Opportunities for Early Learning” in national policies.

**Figure 9:**
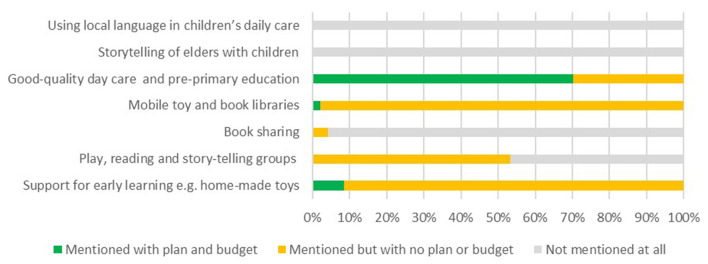
Coverage of “Opportunities for Early Learning” in county integrated development plans.

National policies indicated strong coverage for the sub-domains of play, reading and story-telling groups, support for early learning and quality day-care and pre-primary education, see Figure 8. These areas were also mentioned in CIDPs, see Figure 9, however < 10% of CIDPs allocated budget for support for early learning.

#### Policy-maker reflections on the opportunities for early learning components

The high proportion of CIDPs allocating budget to day-care and pre-primary reflects the progress made on provision of ECDE centers. Across all 47 CIDPs we found the implementation plans mentioned a total of 33,919 ECD centers, both public and private that are already up and running. Seven counties specified plans to increase the number of ECDE centers potentially adding a further 1,455 nationally. Policy and decision-makers, particularly at County and sub-County levels highlighted challenges with the regulation of the ECDE centers. This was seen as a particular problem in urban areas such as Nairobi where a plethora of private and NGO centers have sprung up to meet the high demand, yet lack of policies guiding engagement and regulation with the private sector undermines progress on implementation:

“*We don't have that clear guideline on supporting private institutions, private institutions that are the majority” (COP/PM/012, Ministry of Education, County)*.

Attention to quality is a key aspect of this sub-domain, and it should be noted that of the 19 counties that specified a child: staff ratio in public sector ECDE centers, average ratio was one staff member to 47 children. The National Pre-Primary Education Policy Standard Guidelines (2018) specify no more than 25 children (age 4–5 yrs) per class so even for this age group, this ratio is clearly above the 13 recommended level. Several policy makers also indicated concerns about the quality of ECDE centers:

“*They should offer directions especially on issues regarding nutrition and WASH. They should ensure they offer supportive supervision and mentorship and coaching to the managers of these ECDEs because most of them don't know which food to give the children that have been brought to their 403 facilities, issues to do with water, sanitation and hygiene is also not well looked up, and also during Covid, they broke covid protocols.” (COP/PM/013 Ministry of Health, County government)*.

Most CIDPs did not specify the age-range of children targeted by these ECDE centers. In the CIDPs that did specify the target age for child attending the ECDE centers, none covered children from 0 to 3 years old. This reflects the national policy position with a National Pre-Primary Education Policy addressing the 4–5 year olds, but with little policy focus on those 0–3 years. While the provision of daycare and pre-primary education category scored highly in policy and CIDP coverage, it should be noted that this was limited to pre-primary ECDE centers, and there was very limited mention of center-based care for children 0–3 years. Participants in the qualitative interviews explained that provision for those children 0–3 years was at the discretion of each county:

“*One of the roles of the county government is that, to undertake the early childhood education which now takes care of the three to five years. So, each and every county is supposed to factor that one as a budget proposition. The other area is that Nairobi County right now we have started talking about childcare centers” (COP/PM/012 Ministry of Education, County government)*.

While some counties, such as Nairobi are addressing the challenges of day-care provision for children 3 years and below, an interview with a sub-county level manager, indicates that there may well be a perception that children of this age are cared for at home by their mothers, and therefore center-based care is not necessary:

“*I think mainly children under three years you know have not started school, most of them, so you find them at home. So we give them services at the household level this includes health education to 424 the mothers, treatment of minor illnesses at household level by the CHVs like diarrhea.” (COP/PM/007 Ministry of Health, sub-County government)*.

Furthermore, similar to the challenges of addressing the wider domains of responsive care and learning opportunities, was the lack of a clear ministerial remit for early years' child-care centers. However, several policy makers alluded to recent activity to address this gap:

“*Then the question is for example where are the early centres or centres for taking …. your small baby to be taken care of? Under whose supervision are these centres? We are not sure whether they are under the Ministry of Health or under the Ministry of Education and that's why we must have a policy in place. And it's like a month ago the division brought together key partners from the Ministry of Education different departments nutrition, social services just to try and bring out– Come up with a policy that will address this early childhood development.” (COP/PM/008 Ministry of Health, County government)*.

#### Coverage on safety and security components in national policies and CIDPs

**Figure 10:**
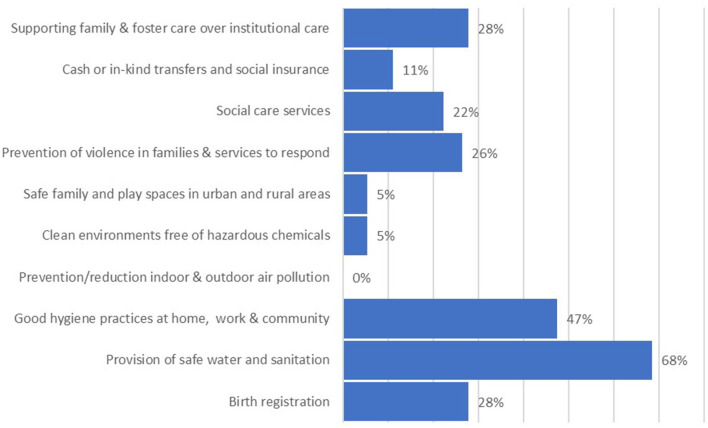
Coverage of “Safety and Security” within national policies.

**Figure 11:**
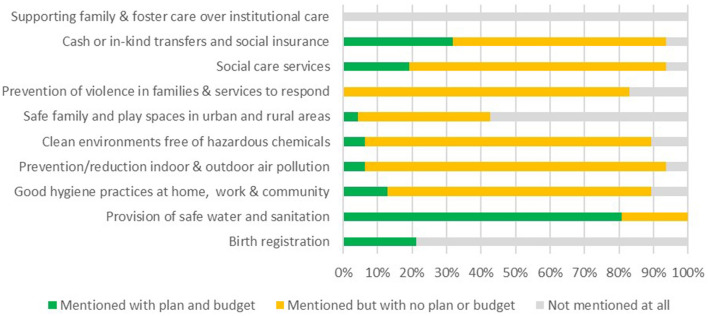
Coverage of “Safety and Security” within county integrated development plans.

Within the “safety and security” domain, the provision of safe water and sanitation was given good consideration both within national policies (Figure 10) and with over 80% of CIDPs including specific plans and budgets to address this vital component (Figure 11). Few CIDPs specified plans and budgets for other key areas within this domain, with no budgets available for prevention and services to respond to violence within families or support for families to foster. Despite the lack of national policy focus on protecting young children from air pollution and hazardous chemicals, over 80% of CIDPs did mention these areas, although few (below 10%) had allocated any budget for them, see Figures 10, 11.

#### Decision-maker reflections on safety and security

Decision-makers highlighted the importance of water and sanitation which was consistent with the focus on this element within current policies and county budgets. County and sub-county policy makers within Nairobi frequently mentioned the difficulties of implementation of adequate water and sanitation for children within urban environments and the impact this would have on health. The other aspect of this domain that emerged as a priority for policy makers was the security of children within schools in terms of protection from violence, security and allowing safe places for play, again, this emerged as a particular issue for decision-makers within Nairobi:

“*A safe environment at school is provided. We have taken care of them in school and also a very good environment for them to play” (COP/PM/012 Ministry of Education, County government)*.

Again, the challenges of providing this within urban environments was recognized:

“*Learners should be in an enclosed place with security, you know the urban areas have a lot of 337 challenges” (COP/PM/009 Ministry of Education, County government)*.

### Challenges in addressing all components of the NCF

The analysis of the qualitative coding displayed in Table 5 reconfirms the overall finding of the review of policies and CIDPs that, while areas of health, nutrition and safety and security have been considered in policies and county level plans (coefficients >0.5, shown in green in Table 5), the domains of early learning and responsive caregiving face significant policy and implementation gaps including future policy plans (coefficients ≤ 0.5).

Budget allocations, particularly within the devolved structures, were identified as one reason behind the limited focus on ECD beyond health and nutrition. There was also a concern of limited clarity on which level of government should fund implementation, with concerns from the national level that despite counties having devolved budgets, activities were left to the national level:

“*Now the only problem is who should carry that burden! You see, like now we are carrying it. The National government may not have allocated funds and money for the National ECD office. It is saying those are devolved functions.” (COP/PM/005 Ministry of Education, National)*.

## Discussion

To our knowledge, this review is the first to systematically scrutinize health and education national policies and all 47 CIDPs in Kenya to assess coverage of the NCF. The review, coupled with the qualitative findings highlight the priority given to nutrition, water, sanitation and hygiene and the limited coverage, particularly in terms of budget, given to the domains of responsive caregiving and Opportunities for early learning domains. This reflects the historical focus on child health in both policy, practice and research, but it is disappointing given the initial progress in Kenya with the integrated ECD Policy Framework of 2006. However, this does reflect the global situation across many LMICs where ECD and education are still an emerging area of government responsibility and scholarship (104). In part, this reflects donor priorities with only 0.5% in 2017, down from 0.8% in 2015, of donor education funding directed to early childhood education (105). Thus, this lack of priority to ECDE is not limited to Kenya but a global phenomenon driven in part by limited funding. Also, most of the national policies that predated the CIDPS did not seem to have a wider coverage on the components of the NCF however, current national policies for example the National Pre-Primary Education Policy Standard Guidelines 2018 and the Kenya Community Health Policy (53, 54), have demonstrated wider coverage for the components of the NCF. This is a positive development for Kenyans, who can look forward to future national policies improving and becoming better at addressing all the components of the NCF.

Our findings, particularly from the qualitative interviews highlight that the complexities of addressing this multi-sectoral challenge require more than funding alone. The challenge of working across sectors in an integrated fashion was a consistent view across our respondents. Yet the need for this multi-sectoral action to address ECD is frequently emphasized (4, 31). The Kenyan example underscores the need to not only consider multisectoral and integrated policy frameworks but also improve coordination and clarity across levels of governance, particularly national and county governments. While the context of devolution presents many opportunities for better integration across sectors within local governments, until there is greater clarity on where budgets and responsibilities should sit, little progress can be made. Despite this, there have been achievements with the establishment and expansion of ECDE centers in many counties. Based on national survey data, UNICEF estimates that 46% of children now participate in organized learning defined as 1 year before the official primary entry age and this is similar to neighboring countries (e.g., Tanzania 56%, Ethiopia 43%, Uganda 34%). However, the rapid growth of private ECDE centers has led to concerns about quality, particularly when the trained workforce and facilities for appropriate provision are consistently lacking (106).

Consideration of responsive care and early learning for children below 4 years of age is a clear gap within national policy and county plans in Kenya. The lack of policy and plans for this age group undermines services and support for nurturing care both within the home and out-of-home interventions such as childcare centers. This is acknowledged as a global policy and practice gap and means that services for the youngest children are often uncoordinated across a plurality of public, private and informal providers with varying quality and inequitable access (107). Where Acts and policies do exist for all children in the early years (e.g., Nairobi Childcare Act 2017) (108), translating these into achievable plans, with budget allocations, is vital. Given the rapidly changing context of families due to urbanization and growing participation of women in the labor force, there is a need to ensure Acts and policies reflect the realities of childcare centers, including those in the informal sector and those that cater for children under 4 years as this review also shows a lack of policies to support early learning at home of children under 4 years. The plurality of childcare providers with the urban context increases the complexity of this task which falls to county governments and requires consideration to regulation, registration, support and monitoring of childcare centers.

Another consistent gap was the limited attention given to the role of fathers within policy and plans. This is despite evidence from national surveys across 38 LMICs identifying a significant relationship between fathers' interaction with their 3 and 4 year olds and their ECDI scores (109). Traditional gendered norms of the roles of mother and fathers continue to influence policy, plans and service provision. These norms help to explain both the lack of emphasis on the role of fathers in caring for their under five children and the lack of provision of quality center-based care to allow women seek opportunities beyond childcare. The World Values Survey asks the question: “when a woman works for pay, children suffer” in 57 countries across the income spectrum. They found that almost half (46%) of those asked agreed with the statement, with nearly as many women as men holding this view and mothers more likely to hold this view than women without children (15).

### Limitations

Although we made efforts to retrieve all relevant documents, county level laws and acts were not available. Furthermore, the qualitative interviews with policy makers only covered Nairobi County and without the remaining 46 counties in Kenya, hence views expressed by the Nairobi County policy makers may not be representative of other counties. As the qualitative interviews were only conducted with officials from health and education departments, the presented perspectives lack the views of those from other departments which deal with children's issues such as social protection.

### Recommendations for policy, practice and research

1. Given the rapidly changing context of families including growing participation of women in the labor force, there is a need to ensure Acts and policies reflect the realities of childcare, as well as those in the informal sector.
2. A thorough review of the Labor Laws of Kenya to understand the extent to which they provide a family-friendly environment for parents, particularly working mothers, through the provision of childcare facilities, maternity and parental leave would be a valuable addition to this research.
3. As much childcare happens in the home in rural areas, good support for this is needed especially in relation to responsive caregiving and support for early learning that can build on existing strengths in the community health strategy.
4. Policies specifying minimum standards and plans which provide support for improvements across all domains of the NCF are needed including family-friendly policies in the workplace and affordable childcare services.
5. Understanding the values and strengths of the local context and community participation is key to feasibility, effectiveness and sustainability of strategies that address ECD.
6. There is also the need to identify responsible department(s) in relation to childcare services and to conduct further research that will help countries to develop appropriate models of care that are feasible for implementation at scale, build capacities for implementation, and obtain context-specific cost data. This data will also support identification of innovative solutions to increase access, quality, and coverage of services.
7. Given the time and resources available for this review, we were unable to analyse budget allocations to the elements of the NCF at national or county levels. Extending this analysis to also cover the extent to which county governments meet the targets set out in their monitoring and evaluation plans would allow a thorough review of progress toward implementation of the NCF.

## Conclusion

This research aimed to establish the extent to which Kenyan government policies address the components of the NCF and to explore policy/decision makers' views on policy gaps and emerging issues. Findings indicate a strong focus on nutrition, water, sanitation and hygiene with clearer policies for children aged 4–5 years and gaps in the provision for children under 3 years in both national policies and county integrated development plans. Furthermore, limited coverage of responsive caregiving and opportunities for early learning domains were found in the CIDPs particularly with a lack of budgeting and plans to involve fathers within early years care. The NCF provides a roadmap for action to address wide range of stakeholders and sectors, conveying key roles involved in giving children the best start in life. Therefore, if nurturing care goals are to be achieved in Kenya, policies are needed to support current gaps identified with urgent need for policies of minimum standards that provide support for improvements across all Nurturing Care Framework domains. Although some Counties within Kenya are ahead of others, this offers opportunities for cross learnings between Counties with inputs required from a range of sectors through policies, services, infrastructure, and information to improve the holistic development of children. Furthermore, early integration of nurturing care relevant content is needed, complemented by in-service training and continuing education of existing workforce. Nurturing care is so embedded within the lives of each family and child thus, communities can play a major role in creating enabling environments that benefit both caregivers and children.

## Data availability statement

The original contributions presented in the study are included in the article/Supplementary material, further inquiries can be directed to the corresponding author/s.

## Ethics statement

The studies involving human participants were reviewed and approved by Health Sciences Research Governance Committee, University of York and Amref Research, and Ethics and Scientific Review Committee. The patients/participants provided their written informed consent to participate in this study.

## Author contributions

HE, MA-O, MN, GO, EH, RH, and PG developed the protocol and concept of the review. MA-O, PK-W, KO, IC, MW, RM, LO, EH, MM, EM, and HE screened and extracted data from the included policy documents. MA-O and HE synthesized the policy data findings, conducted the overall synthesis of results, and drafting of the manuscript with input from MN, GO, PA, LO, RH, and PG. All authors read and approved the final manuscript.

## Funding

This research was funded as part of a British Academy Award Grant Ref: ECE190115 and Medical Research Council GCRF Impact Fund Ref: G0086101 0026 for funding the article publication charge.

## Conflict of interest

The authors declare that the research was conducted in the absence of any commercial or financial relationships that could be construed as a potential conflict of interest.

## Publisher's note

All claims expressed in this article are solely those of the authors and do not necessarily represent those of their affiliated organizations, or those of the publisher, the editors and the reviewers. Any product that may be evaluated in this article, or claim that may be made by its manufacturer, is not guaranteed or endorsed by the publisher.
