# Supplementary material for: Improving early childhood development in the context of the nurturing care framework in Kenya: A policy review and qualitative exploration of emerging issues with policy makers
Source: Front Public Health. 2022 Sep 27;10:1016156. doi: 10.3389/fpubh.2022.1016156 (PMC9551223; doi:10.3389/fpubh.2022.1016156)
Supplement: Supplementary file 1 [file Table_1.DOCX]

**Supplementary Table 1: Data for the Components of the Nurturing Care Framework as addressed in the Kenyan 47 County Integrated Development Plans (CIDPs)**

| **County & CIDP/year** | **Age (0 to 5)** | **Centre-based care in relation to under-fives (ECD/ECDE)** | **Nurturing Care Framework** | | | | |
| --- | --- | --- | --- | --- | --- | --- | --- |
|  |  |  | **Good health** | **Adequate nutrition** | **Responsive caregiving** | **Opportunities for early learning (ECD/ECDE)** | **Security and safety** |
| 1.Baringo | 0 to 5 | Currently the county has 1012 functional public ECDE with an enrolment of 47,409 pupils. There are 60 upcoming ECDE centres. The county has employed 1772 ECDE teachers and 9 ECDE co-dinators, who oversee activities in the sub counties. The county has 331 special needs children (4 to 9yrs) in ECCDE with 12 ECCDE teachers. The county has established a college which trained ECDE teachers who` are employable in various centres as well as self-employment in private ECDE centres. | **a**. Current access to and uptake of family planning services is still low and this needs to be stepped up. **b**. Proportion of children under one year who are fully immunised currently stands at 52%. The ideal situation is to have all children under-5 immunised against vaccine preventable diseases, to increase community awareness of the dangers of missing immunisations. **d**. Currently there are 313 mothers needing prevention of mother to child transmission (PMTCT). **f**. To encourage pregnant women to attend antenatal care (ANC) clinics. Current proportion of skilled delivery is 40% with plans to increase to 60%. | **b**. More mothers are currently adhering to exclusive breastfeeding. The county plans to carry out one world breastfeed week every year in this current CIDP. **c**. ECDE meal and nutrition programme has been proposed to support provision of meals and nutritional interventions for public ECDE centres in the County. **d**. This CIDP plans to increase Vitamin A supplementation, **f**. to reduce children who are underweight to 10%, wasting to 5% and stunting to 20% and **g**. increase deworming for children. **i**. Support of nutrition activities has helped in reducing complications related to underweight, malnutrition and stunting rates. | **d**. ECDE Meal and Nutrition Programme is proposed for this CIDP in cognizance of a myriad of challenges of low access, poor retention, transition, attention and inconsistent enrolment rates in public ECDE centres in the county. **e**. The current CIDP proposes construction of six model ECDE centres per Sub-County to provide a conducive environment for learning that meets the recommended global standard as a benchmark for quality ECDE in the county. **f**. The centres will preferably comprise of 4 standard classrooms, playground well equipped with modern ECDE play infrastructure, modern ablution blocks and a standard kitchen with a dining area. | **a**. The current CIDP plans to ensure inclusive and equitable quality education by providing all girls and boys access to quality early childhood development by 2030 to promote lifelong learning opportunities for all. **d**. There are three main operational libraries in the county however, these existing libraries are concentrated in Baringo central sub-county therefore the is a need for special consideration to the other 5 sub-counties.  **b & f**. Construction of Kampi ya childcare centre and to equip the centre with modern play equipment as well as construction and upgrading of ECDE classrooms to improve learning and performance | **a & g**. Provide security; carry out inspections on matters pertaining to children and enforcement of children rights, issuance of children birth certificates, sexual and gender-based violence, preparation of court reports on matters pertaining to children and enforcement of children rights. **b & c**. The current CIDP plans a construction & rehabilitation of water supplies to increase access to clean and safe water, reduce distance to water points to the acceptable standard of 30 minutes’ walk, as well as increase access to sanitation and hygiene facilities. **d**. There are plans to control air pollution, noise pollution, other public nuisances, and outdoor advertising. **e**. The county intends to enhance access to quality health and a sustainable access to clean environment for living to the residents of Baringo. **f**. There are also plans to establish public recreation parks in each sub-county on at least five acres of land as well as construction of county player’s theatre/social hall. **h & i**. The county has plans to reduce poverty in the households living with vulnerable persons through the provision of grants, cash transfers and loans; awareness creation on the persons with severe disabilities cash transfer program (PWSD-CT), up scaling PWSD-CT pro-grams and monitoring and evaluation on the cash transfer programs. |
| 2.Bomet | Under 5 | The county currently has 1,221 public ECDE with 2,022 teachers. The private centres have 350 centres with 424 teachers managed by individual proprietors and faith-based organizations. The enrolment as per the current statistics is 53,727 children in public centres and 12,981 children in private ECDE centres. Teacher-pupil ratio is stands currently at 1:58 in public and 1:27 in the private centres. | **a**. The current uptake of family planning among women of reproductive age (15-49) is 47.5% which is low compared to the national of 55%. The county plans to increase investments targeting family planning services to improve access, intensify programmes to address myths and misconceptions on certain family planning methods. **b**. Immunization coverage for children between the ages of 12 to 18 months is currently 81%, which is above national average of 68%. **d**. Currently pregnant mothers receiving preventive antiretroviral therapy (ARV) stands at 98.7%. **f**. Pregnant women who currently attend 4 or more ANC stands at 37% with 52% receiving skilled delivery. | **a**. Poor nutrition has been highlighted as a challenge in maternal healthcare. **b**. The SDGs have been mainstreamed in several policy documents including breastfeeding policy. **d & g**. This CIDP plans to increase Vitamin A supplements and deworming programmes as well as establishment of feeding programme for ECDE. **f & i**. The nutritional status highlights that stunting and wasting in children under the age of 5 is at 38% in the county. Hence this CIPD plans to establish feeding programmes to manage malnutrition and stunted growth. | **d**. This CIPD plans to establish feeding programmes to manage malnutrition and stunted growth. **e & f**. Also, to expand and establish ECDE centres as well as capitation fund to facilitate provision of teaching, learning materials and support for modern and well-equipped learning ECD centres in every public primary school.  . | **a**. Opportunities for early learning are provided under ECD with well-equipped modern learning ECD centres in every public primary school. **d**. The county government is in the process of setting up community-based libraries with ICT centres in every sub-county. **e**. The enrolment of children per current statistics in public centres is 53,727 and 12,981 children in private ECDE centres. The transition and completion rates are at 89% and 95% respectively while retention rate stands at 80%. | **b & c**. The county aims to increase access to clean and safe water by developing county water master plan, water policy and water bill, as well as increase access to sanitation and hygiene facilities. **d & e**. Also, effective enforcement of environmental and natural resources policies, control of in-door air pollution as well as ensuring a clean environment. **h & i**. The social protection programme which targeted persons with severe disability was started following the enactment of the Bomet County Support for the Needy Act, 2014. The programme entailed cash transfers to persons with disabilities and also in the payment of the National Hospital Insurance Fund (NHIF) premiums to cater for their medical cover. |
| 3.Bungoma | Under 5 | The county currently has 834 public ECDE with 3,290 teachers. The private centres are 458 with 1,316 teachers. The enrolment as per the current statistics is 110,335 children in public centres and 25,114 children in private ECDE centres. Teacher-pupil ratio currently stands at 5:33 in public and 1:19 in the private centres. | **a**. Family planning by women  currently married is 54% with urban and rural areas at 54% and 55% respectively. Half of the women aged 20-24 years use family planning, as well as over 60% of women aged 25-34 years, declining thereafter. **b**. 73% of children aged 12-23 months are fully immunized compared to a national average of 68%. The percentage of children fully vaccinated is higher for rural areas (71%) than for urban areas (59%). **c.** Key public health issues such as smoking, and alcohol consumption will be addressed as factors affecting a person’s wellbeing. **f.** Currently, almost 9 in 10 mothers receive more than 1 ANC and half of mothers received at least 4 ANC. 50% of births were delivered by skilled personnel. **h**. Overall, 60% of new-borns received a health check following birth while in a health facility or at home. | **b**. The county plans to monitor the proportion of ECDEs provided with breastfeeding; **c**. implementation of the School feeding programme in 860 ECDE to provide nutritional feeds to children; **d**. Vitamin A supplementation to children as well as iron and folic acid supplements for pregnant women. **f**. Currently children under 5 years who are stunted stand at 24% compared to 26% nationally, 2% are wasted compared to 4% nationally, those underweight stand at 9% against 11% at the national level and overweight or obese are 3% compared to 4% nationally. **g**. There is deworming services provided to school children. | **d**. The county plans to monitor the proportion of ECDEs provided with breastfeeding; implementation of the School feeding programme in 860 ECDE to provide nutritional feeds to children. **e**. Also, ECDE centres to be provided with toys and outdoor play facilities, **f**. Current investments have contributed towards improving the teaching/learning  environment in ECDE through measures such as construction of infrastructure facilities, recruitment of ECDE teachers and instructors, undertaking  quality assurance and standards in ECDE centres. | **a**. Current investments have contributed towards improving the teaching/learning  environment in ECDE through measures such as construction of infrastructure facilities, recruitment of ECDE teachers and instructors, as well as providing toys and **b**. outdoor play facilities. **d**. The County has Kimilili community library which is under Kenya Library Services as the only public library in operation. **e**. The county currently has 834 public ECDE with 3,290 teachers. The private centres are 458 with 1,316 teachers. The Gross Attendance Ratio for pre-primary is 101.5% compared to the national average of 94.4% with variability among rural and urban areas. | **b & c**. The county plans to improve access to safe water sources and decent sanitation through construction, operation and maintenance of appropriate community safe water supply systems as well as strengthening collaboration amongst the institutions responsible for sanitation activities and law enforcement bodies with regards to sanitation and hygiene. **d**. Plans for pollution control management through mitigation of air, sound and odour pollution to improve the health of all Kenyans as well as **e**. ensuring a clean environment free from contamination and ecological degradation. **f**. The County shall prioritise investments in maintenance of designated public open spaces and in community and recreational facilities including development of talent centres for the populace. **g**. Preventing and responding to gender-based violence, **h** There are plans to expand the scope and coverage of the social security services to poor and vulnerable households as well as protect their rights against abuse, exploitation, violence and  neglect. **i**. There is also National Safety-Net  Programme (Cash Transfers). |
| 4.Busia | Under 5 | There are 919 ECDE  centres in the county of which 440 are public and 479 private. The teacher: pupil ratio stands at 1:100. The current CIDP commit to expand on  ECDE learning facilities and recruit additional 900 ECDE teachers. | **a**. Family planning uptake in the county among women of reproductive age (15 – 49 years) stands at 34%. Contraceptive acceptance stands at 46.5% among females aged 18 years and above.  **b**. Immunisation coverage of children under 5 years was over 69% in the county with all sub - Counties reporting good response to all immunization campaigns carried out. **c**. Awareness campaign on control of alcohol and drug abuse as well as support for victims. **d**. Currently HIV+ mothers receiving preventive antiretroviral (ARVs) stands at 101%. **f**. Pregnant women currently attending 4 ANC visit stands at 42% with skill delivery at 51%. | **b, c & d**. Currently 27% of children have access to nutritional services covering exclusive breastfeeding, 61% have Vitamin A supplement and 39% pregnant women receive Iron supplements for at least 90 days. There are efforts to improve provision of food supplements enhancing food access, provision special nutrition interventions for specific vulnerable groups and creating awareness to provision of nutritious food to all family members and especially children.  Improved health of ECDE learners. **f**. 31% of the children below five years are malnourished while 26.5% are stunted hence plans to improve health of ECDE learners by  increasing ECDE boys and girls provided with milk and supplements. **g**. There are 15% of children that are currently dewormed with plans to increase this percentage. | **d**. There are efforts to improve provision of food supplements enhancing food access, provision special nutrition interventions for specific vulnerable groups and creating awareness to provision of nutritious food to all family members and especially children. **e & f**. ECDE is mandated to build capacity of ECDE teachers and improving the physical environment of the ECDE children through construction of classes and provision of outdoor play equipment as well as supported with grant for equipping and purchase of teaching and learning materials to improve quality of learning. **i**. The county has a total of 110,000 Orphans and Vulnerable Children who are entirely dependent on relatives and well-wishers who volunteer to assist them with other social support facilities for care and educational support. | **a & b**. The directorate of ECDE is mandated to supervise ECDE curriculum implementation, monitoring and development of ECDE children, collect data for equitable distribution of resources and improving the physical environment of the ECDE children through provision of outdoor play equipment. **d**. There are seven library and information documentation centres in all seven sub – Counties. **e**. The county has 919 pre-school ECDE centres attached to public primary schools and private ones and the County Government recruited 439 ECDE teachers in 2013-2017 period. The County had a population of 51,160 attending pre-school however the total pre-school population within the county stands at 71,519 in 2018. | **b & c**. The county plans to rehabilitate dilapidated infrastructure in order to improve access to safe water, sanitation and hygiene, including reducing distance travelled and ensuring adequate water supply & reliability of sanitary and hygiene facilities. The benefits of having access to improved drinking water source can only be fully realized when there is also access to improved sanitation and adherence to good hygiene practices. **d**. There is control of air pollution, noise pollution, other public nuisances. **e**. And plans to improve environmental education, urbanization, sand harvesting, land management practices and sustainable management of ecosystem to keep the environment clean. **g**. Gender Based Violence. **h &** i. There are appropriate social security to vulnerable groups in the society including plans to establish more child protection centres, and enhancing safety nets and social protection programs like cash transfer programme for persons living with disability as well as orphans and vulnerable children with about 8,630 beneficiaries on cash transfer. |
| 5.Elgeyo-Marakwet | Under 5 | Currently there are 180 ECD classrooms completed and equipped with special need learners’ friendly structures and a pupil: classroom ratio of 94:1. 778 ECD teachers have been recruited with 44:1 pupil: teacher ratio | **a**. Contraceptive prevalence among women in the reproductive age group in the County stands at 44.1% and the county leads in the use of traditional family planning methods **b**. Currently, immunisation coverage for children under one stand at 66.3% which is lower than the WHO recommended standard of above 80%. **c**. There are plans to eliminate drug and substance abuse, **d & h**. and to promote neonatal and child survival, scale up elimination of Mother to Child Transmission of HIV/AIDS services and enrol all HIV positive clients into the ART programme. **f**. Mothers currently completing 4+ ANC visits is 17.3%, and deliveries by skilled attendants stand at 41.8% which is below the national estimates of 40% for ANC visits and 60% for skilled delivery. | **a, b & c**. There is currently poor maternal nutrition during pregnancy and at postpartum with mothers not practising exclusive breastfeeding and early introduction of complementary feeds at 2-3 months of a baby.  Exclusive breastfeeding of children is currently 30% with plans to increase it to 40%.  **d**. Vitamin A supplementation to children 6-59 months is under 20% and 27.3% of pregnant women are supplemented with Iron Folic Acid with plans to increase Vitamin A supplements to 45% and Iron Folic Acid to 50% for pregnant women. **f**. Children under-5 years who are underweight is 12% and those stunted at 32% with plans to reduce underweight to 7% and stunting to 20%. **g**. There is a deworming program. **i**. Hence the need to reduce prevalence of underweight and stunting among children aged less than 5 years by improving nutrition and increasing uptake of micronutrients among children. | **d**. The county plans to supplement 9,000 households with Micro-Nutrient Powders, as well as prioritise feeding programs to enhance access, retention and transition to 90%  **e & f**. The county government invested in the employment of ECD teachers, construction and equipping of pre-primary school classroom and the introduction of capitation grant in pre-primary and sensitization of parents/caregivers. | **a**. The county plans to provide pre-primary education for all children as well as a conducive environment for learning, including establishment of ECD learning Resource Centre, capacity building of staff.  **d**. There are plans to complete and furnish ICT Centre with a library. **e**. The Pre-primary education Gross Enrolment Rate in ECD centres is expected to rise from 75% in 2017 to 95% in 2022. Key achievements included increased enrolment from 31,110 in 2013 to 34,464 in 2017 for pre-primary education. | **b**. The county plans to increase access to clean and safe water, improved liquid and solid waste management systems. **c**. Scale up of sanitation and hygiene in households and within the community. **d**. Control noise and air pollution. **e**. There are plans to ensure all county residents have sustainable access to high standards of health and sanitation in a clean and healthy environment free from hazardous wastes contamination of site and debris management. **g**. Other issues which affect the participation of women in productive ventures are high incidences of gender-based violence and discriminatory traditions, policies and laws which violate women’s rights. **h**. There are plans to strengthen the design and implementation of inclusive policies and social safety net mechanisms, including community involvement integrated with livelihood enhancement programmes. **i**. It also strived to protect the vulnerable and improve child welfare with 4,012 households currently benefitting from the Orphans and Vulnerable Children Cash transfers program. |
| 6.Embu | Under 5 | There is a total of  619 ECDE centres with 399 public and 220 private centres. The teachers to pupil’s ratio are 1:42. There is a higher number of boys than girls enrolled in public ECDE by 4.28% and 9.04% in private ECDE.  There are plans to build 20 model ECDE centres every year. | **a**. Currently 28% of women in the reproductive age use family planning with plans to increase to 70%. **b.** Also, 80% of children under 1 year are fully immunized  with plans to increase the immunisation rate to 92%. **c & e.** There are plans to Increase the number of identified drug and substance users and to rehabilitate them as well as increase uptake of mental health services. **d**. The estimated number of pregnant mothers living with HIV currently stands at 487 with 105 PMTCT sites. **f**. There are 48% of mothers attending 4th ANC and 65% receiving skilled deliveries with plans to increase ANC and skills delivery to 70% and 82% respectively. | **b**. The county plans to promote breastfeeding. **d**. 50% of ECDE children and 49% of under-fives are currently receiving vitamin A supplements with plans to increase to 100% and 75% respectively. **f & i**. 26.8% of children are stunted, 11.1% are underweight and 3.0% wasting with plans to reduce stunting to 21.8%, underweight to 6.6% and wasting to 1.8%. Efforts to prevent the current rates of malnutrition from worsening and correcting the situation includes increasing access to integrated management of acute malnutrition program and knowledge and skills gap among health care providers and nutrition status assessment using child growth standards. | **d**. Currently, 17000 children are under milk feeding programme to improve health, access, retention & completion of ECDE. **e**. There are plans to purchase of play equipment to provide holistic development of children. **f**. Employment of new ECD teachers to continue enhancing and improving the quality and relevance of early childhood education. Training and capacity development, strengthening ECDE management to improve teacher learner’s ratio and curriculum delivery. **g**. Increase uptake of mental health services. | **a**. The county is giving greater attention to cases of disadvantaged and vulnerable children with disabilities in ECDE by improving their access to education and improving the quality and relevance of early childhood education. **b**. There are plans to purchase of play equipment to provide holistic development of children.  **d**. There are also construction of rehabilitation centre, library and resource centre. **e**. 98 ECDE centres have been constructed and 22 ECD centres renovated to improve learning environment for ECD pupils including pupils with special needs. | **b**. The county plans to improve access to clean and safe drinking water and proper sanitation by providing adequate and reliable water and improvement of storage capacity; **c**. as well as access to adequate and equitable sanitation and hygiene for all and to end open defecation, paying special attention to the needs of women and girls and those in vulnerable situations. **d**. Control of air pollution in the county, which are mainly from motor vehicles and dust. **e**. Maintaining a clean environment through Environmental Impact Assessment and ensuring that programmes being implemented in the county are complying with the environmental standards. **f**. Establishment of a recreation centre and people’s park at Masinga dam for public access. **g**. There are plans to establish human dignity centre to support and reduce cases of Gender Based Violence as well as improve wellbeing of victims. **h & i** The children department through the cash transfer programme will address some of the main issues that face the OVCs in the society by targeting the most vulnerable in the society. Child labour which is prevalent in the upper and lower parts of the county will also be addressed through community sensitisation. |
| 7.Garissa | Under 5 | There is a total of  281 ECD centres. There are 229 ECD teachers with teachers to pupil’s ratio 1:72. The total enrolment of pupil is 11,874 with 6,817 boys and 5,057 girls and a gross enrolment rate of 65%. | **a**. The county has a very low contraceptive acceptance rate of 6%, which is attributed to the cultural and religious practices that prohibit family planning. Hence the county plans to ensure universal access to family planning services. **b**. The vaccination coverage is 62%, which is attributed to the  inaccessibility of the area, long distances to health facilities and poor road network. The county plans to increase immunization coverage through increase in vaccine potency. **c**. There are plans to develop and implement alcohol and drugs abuse policy, **d**. and to scale up elimination of mother to child transmission. **f**. Maternal health care in the county has improved having attained antenatal and postnatal coverage of 48%, and skilled delivery stands at 52% and home delivery is 48%. | **a**. Increased uptake of Maternal, newborn, child health and nutrition services including reducing children <5yrs who are under weight and stunted. **f & i** The county plans to provide sustainable and effective feeding program to ECDE children including addressing nutritional status such as prevalence of stunting and wasting in children under 5 years, manifesting as height-for-age, weight-for-height, weight-for-age; and to increase uptake of nutrition services to improve the status of the child’s health, care and nutritional needs and link him/her to health check-ups and growth monitoring. | **d**. The county plans to provide sustainable and effective feeding program to ECDE children including addressing nutritional status such as prevalence of stunting and wasting in children under 5 years. **e & f**. There are plans to provide indoor and outdoor materials, which include fixed equipment, swings, slides,  see-saw, rocket frames tunnels, three, climbers etc., and make movable play materials, that is tires, halls, rings, ropes, etc, as well as provide teaching and learning materials like blackboards, chalks, pens, manila papers, paints, brushes, basins, blocks, puppets to enable the child to enjoy living and learning through play. Also, provision of high-quality infrastructure that is classrooms, sanitary facilities, offices, stores, kitchens, playgrounds. | **a**. The county’s priority is to improve accessibility of ECDE learning centres to provide education geared towards development of the child’s mental capabilities and physical growth as well as to enable the child to enjoy living and learning through play. **b**. There are plans to provide indoor and outdoor materials, which include fixed equipment, swings, slides, see-saw, rocket frames tunnels, three, climbers and make movable play materials, like tires, halls, rings, and ropes. **d**. Purchase of  mobile library, vans, books box,  motor bikes, library furniture and equipment and current  relevant resource materials.  **e**. There are ECD/ECDE centres that provides holistic early childhood development services through coordinated  partnerships, integrated quality service delivery and safeguarding the child’s rights and welfare as well as enabling  the child to develop understanding and appreciation of his/her culture and environment. | **a**. The county provides legal identity for all, including birth registration. **b & c**. The county plans to increase access to minimum amount of safe water supplies in rural and urban areas and to ensure hygiene and sanitation commodities for all to end open defecation. **d**. Control of air pollution, noise pollution, other public nuisances, and outdoor advertising. **e**. The Kenya Vision 2030 aims to provide a high quality of life to all its citizens in a clean and secure environment. **f**. The county plans to construct friendly spaces equipped with play materials-recreational parks/playgrounds and facilities in all the sub  counties. **g**. Elimination of all forms of violence against all women and girls in the public and private spheres. **h**. Provision on health insurance to vulnerable households (child-headed and vulnerable households). To empower marginalized/ needy individuals and communities for effective participation and sustainable social economic development. **i**. Cash transfer to orphans and vulnerable  child of about 1400 annually |
| 8. Homa Bay | Under 5, although the focus of pre-primary education is 3 to 5 years | The county employed on contract 1,319 ECDE teachers who have been deployed to at least one in the 876 ECDE centres in the county. This has increased the number of ECD pupils from 20,000 in 2013 to 86,859 by the end of 2017, with a percentage increase of 400%. | **a**. The contraceptive prevalence rate is 46.7%, which is less than the national average of 58%, and 50.3% of women of reproductive age are receiving family planning. **b**. The current immunisation rate stands at 64.4% hence the county plans to improve on the immunization coverage to enhance child health expectancy. **d**. The county noted improvement in women giving birth in facilities and HIV positive mothers getting prophylaxis. **h &** **j** Currently the county has 8 comprehensive emergency obstetric and neo-natal care and 25 basic emergency obstetric and neo-natal care facilities to respond to obstetric and neo-natal emergencies. | **b**. The current exclusive breastfeeding rates are at a low of 38% compared to the national average of 61%. Early initiation of breastfeeding among new-borns was 67%, which is below the national target of 90%. **c**. Integration of nutrition services in the ECDE centres is ongoing to ensure that all ECDE children are reached on a regular basis. **d & g**. Vitamin A supplementation and deworming coverage are low and below the national targets of 80% with only 43.8% of children 12 to 59 months supplemented with vitamin A and 7.1% dewormed. **f**. The stunting levels are high at 21.8%, underweight of 8.6% and wasting of 4.6%. This shows a decline compared to stunting levels of 18.7%, underweight of 5.4% and wasting of 4.1% in 2014. | **d**. Integration of nutrition services in the ECDE centres is ongoing to ensure that all ECDE children are reached on a regular basis. **e & f**. The county plans to establish indoor and outdoor play equipment with teaching and learning materials, as well as recruit and remunerate adequate ECDE teachers, supervisors and inspectors to deliver, monitor and evaluate ECDE quality service delivery. | **a**. The county plans to ensure that all children enrol and complete full cycle of free, equitable and quality ECDE leading to relevant and effective learning outcome. **b**. The county plans to establish indoor and outdoor play equipment with playgrounds, Crèches and play kits for teaching and learning. **d**. Homa Bay County currently has no public library hence the current CIDP has prioritized the construction of a County Library in Homa Bay Town. **e**. The county has ECD and ECDE centres which are conducive and the county supplies teaching and learning materials to all public ECDE learning centres to ensure that all children have access to quality early childhood development, care, support and pre-primary education. | **b**. The county plans to sustain and manage water resources to provide access to safe water and sanitation as well as improvement of access to water in all ECDE Centres through improved water harvesting, storage and treatment. **c**. There is also ongoing installation of tipping bins and waste transfer stations, construction of VIP latrines as well as promoting hand washing in all ECDE Centres. **d & e**. Control of air pollution, noise pollution and protection of nation parks and strengthen environmental governance by implementing climate change strategies to keep the environment safe from pollution.  **g**. The county is implementing policies on prevention and response to gender-based violence including handling of cases and prevention against domestic violence. **h**. The county social protection policy has been developed and approved by the Assembly to guide all measures of alleviating suffering for vulnerable households. **i**. The number of Orphans and Vulnerable children benefiting from cash transfers is currently estimated at 11,069. |
| 9. Isiolo | Under 5 | There are 160 ECDE centres of which 42 are private. 2017 total enrolment is 16,295 with 9,102 being boys and 7,193 girls. The teacher pupil ratio in the ECD centres is 1:87, which is far above the optimal of 1:40 implying that the county is running short of ECD teachers. | **a**. Access to family planning is a challenge with more than 60% of those in the reproductive age currently have access to family planning. The contraceptive acceptance rate stands at 25%. **b**. Immunisation coverage currently stands at 63.5% of children under one. **d**. The county is promoting prevention of Mother to Child Transmission of HIV as well as increasing the proportion of HIV+ pregnant mothers receiving preventive ARV‟s. **f**. 46% of pregnant women are currently attending 4 ANC visits with plans to increase to 95% and skilled deliveries stand at 51%. | **b & c**. Recent Knowledge, Attitude, Beliefs and Practices survey conducted in 2017 show that exclusive breastfeeding is high 74.2%, however complementary feeding practices remains suboptimal with minimum acceptable diet at 24% and minimum dietary diversity for women for More than 5 food groups at 27.4%. **d & g**. Pregnant women receiving iron foliate at least 90 days stands at 20% with plans to increase to 90% and children aged 6-59 months receiving Vitamin A twice a year also stands at 20% with 33% of school age children dewormed. **e & f.** Causes of malnutrition in the county include inadequate quantity and diversity of age specific foods. One out of 5 children under 5s are wasted, stunting also remains high at 19.1% compared to the national average of 26%. | **d**. The county plans to establish a functional school feeding programmes to improve enrolment levels such as reintroduction of mid-morning snack (porridge) and lunch. **e & f.** The high population of the under 5s group calls for development of more ECD Centres including provision of adequate play materials and equipment (indoor and outdoor). Currently 90% of the under 5s are supposed to be attending ECD centres but because of drought and inadequate ECDE infrastructures and pastoralism this is not the case. Hence the county plans a rapid infrastructure development, increase teaching manpower in ECD centres as well as accommodation for ECD teachers. **i**. Empowering the most vulnerable families or caregiver to be able to provide well for their children as well as strengthen community led care for the children. | **a**. The county plans to improve access to early learning opportunities by recruiting 345 ECDE care givers on contract, construct and equip 20 ECDE Centres per year as well as improve quality assurance in all ECDE services and provision of learning materials. **b**. There are plans to provide adequate instructional/ learning materials and play equipments in ECDE Centres. **d**. There is only one public library in Isiolo town which is ill equipped as it lacks necessary learning materials, hence there are plans to equip the library and open up more libraries in the sub-county levels. **e**. There has been increased enrolment of pupil to ECDE from 8,991 in 2013 to 16,295 in 2017 after construction and equipping of equipping of 37 ECDE Centres. | **a**. Birth registration is low in Isiolo County especially for pastoralist children who were not born in health facilities. Hence there are plans to decentralize birth registration services to the sub-counties so that children in rural areas can access birth registration services. **b & c**. There are plans to increase urban population access to clean and safe water as well as aim at achieving access to adequate and equitable sanitation and hygiene for all and end open defecation while improving the existing sanitation facilities. **e**. Reducing environmental pollution including chemical pollution from the Tannery Improved solid waste management and pollution control degradation are planned for the current CIDP. **f**. The county plans to construct rehabilitation centres and recreational parks and facilities for use in urban areas. **g**. Linkage with gender-based violence survivors by providing health and legal services for victims. **h &** **i**. The county has been running programs that provide support to most vulnerable children, which includes social safety /net programmes being run by national government (cash transfer programme) for orphan and vulnerable children and people with disability. There are also plans to establish special programs for street families and their rehabilitation, establishing livelihood kitty fund for the vulnerable groups and child protection units. |
| 10.Kajiado | Under 5 | Kajiado County has a total of 888 ECD Centres with a total population of 61,225 children and 53% are boys. Net enrolment rate is 86%. There are facilities that cater for children with special needs such as the Enkijape pre-primary in Loitokitok for hearing impaired and AIC Childcare in Kajiado for multiple needs. The overall retention rate stands at 67% with Kajiado East leading at 98%. The completion and transition rates stand at 83 and 89% respectively. | **a**. At least 45.2% of women aged 15-49 currently use any method of contraception, hence the county plans to increase family planning awareness. **b**. 84% of children under one are currently fully immunized which is slightly below the national fully Immunized children target of 90%. The county aims to scale up immunisation programme to attain 90% coverage. **c**. There plans to reduce alcohol drug and substance abuse. **f**. 96.7% of women between ages 15-49 receive antenatal care from a skilled provider; 62.4% of births are delivered in a health facility and 63.2% are delivered by a skilled provider. **k**. The county plans to scale up Integrated management of childhood illness both at the facility and community level. | **a & b**. The county plans to create awareness on the importance of exclusively breast for infants less than 6 months of age and create community awareness on maternal nutrition. **d & g**. There are plans to scale up vitamin A supplementation and deworming among children under 5. **f & i.** Prevalence of stunting (low height-for-age) in children under 5 stands at 25.3% while wasting (low weight-for-height) is at 10%. Efforts are ongoing to combat undernutrition and malnutrition and make progress towards achievement of sustainable development goals to ‘End hunger, achieve food security and improved nutrition and promote sustainable agriculture’. | **d**. The county is formulating and implementing school feeding program in all ECD centres to improve nutrition as well as programs to support retention of learners such as feeding, school shamba program to supplement nutritional requirements for children in this age category. **e**. There are plans to develop ECD schemes of service, employ qualified ECDE teachers and care givers and to enhance remuneration ECD teachers for teachers training of ECD teachers. | **a**. The county has plans to ensure that education facilities for the early learners are available and accessible to all children across the county as well as invest in fully fledged ECDE that are fully equipped to provide an enabling learning environment for all children in ECD centres. **d**. The county is in the process of completing the construction of a public library. **e**. The county has constructed 327 ECD classrooms across the county to improve learning infrastructure in areas where education came second to livestock herding and where long distances from learning centres deterred enrolment. The county also hired 560 ECDE care givers and deployed them to various schools across the county. | **b & c**. The county plans to increase access to safe and clean water and tackle challenges linked to drinking water, hygiene and sanitation for populations in addition to water-related ecosystems. **d & e**. There are plans to regulate the agrochemical supplies, enforce the law to ensure use of standard pesticides, educate farmers on best use of pesticides and other agrochemicals to reduce air and environmental pollution. **g**. There is also a framework to respond to gender-based violence in the county, sensitize women on their rights and gender mainstreaming as well as develop and review relevant gender policies and laws. **h**. Most children in Kajiado are at risk due to increase in divorce and separation cases, family negligence and breakdown of social fabric. Currently, there are 26,719 children in need of social protection within the county hence the county plans to establish rehabilitation program for orphans and vulnerable children. **i**. The county plans to provide cash transfers for qualified vulnerable groups as well as mobilise funding for the care and support of vulnerable groups. |
| 11.Kakamega | 3 to 5 | There are currently 1,943 ECDE centres with 924 centres supported by the county government while the remaining 1,019 centres are privately owned. The ECDE centres have a total teaching force of 4,702 ECDE teachers out of which 4,526 are trained and 176 are untrained. The current ECDE enrolment is 117,266 distributed as; 95,979 in public ECDE centres and 21,287 in private ECDE centres. | **a**. The current use of any method of family planning among married women aged 15-49 is 62% higher than the national average of 58%.  The county plans to promote family planning and sensitise on relevant contraceptives that also target men. **b**. Immunization coverage of the children aged 12-23 months who are fully immunized currently stands at 62%. **c**. Alcohol and drug abuse control is part of this CIDP. **d**. The county plans to establish Mobile Voluntary Counselling Testing and prevention of mother to child transmission services. **f.** The current percentage of pregnant women who received first antenatal care from a skilled provider is 96% equalling the national average and those who attended the 4th antenatal care is 65%. Births assisted by a skilled provider stood at 49% below the national average of 62. **g**. Furthermore, free maternity services are offered in all County hospitals and 33,000 needy mothers have been supported financially to improve child and mother survival rate. | **b**. The county plans to increase the current proportion of 39% of children below 6 months on exclusive breastfeeding. **d & g**. There are plans to reduce malnutrition by increasing the proportion of 6-59 months children administered on vitamin A and mothers receiving IFAS supplements as well as increase in the percentage of school age children dewormed. **f**. The nutrition status in the county currently stands at 8.6% of the under 5s who are underweight with 77,444 mainly from poor households severely or moderately undernourished. **i**. There are plans to sensitise the community on proper feeding methods and to enhance community strategy to boost the capacity of the community in handling nutritional issues. | **d**. County ECDE School have feeding programmes to improve malnutrition among children. **e & f**. The county also plans to construct and equip ECDE centres (including furniture, play equipment, teaching, and learning materials) as well as employment of more ECDE teachers, conduct quality assurance and standards assessment. | **a & b**. Attendance to early childhood education is 40%, support for learning is 63.3%, availability of ECDE children’s books is 3.7%, availability of ECDE children’s play materials is 69.3%, while inadequate care stands at 40.1%. **d**. The county has two Libraries to enhance reading culture and access to Library services. **e**. So far, the county supported ECDE centres enjoy services provided by a total of 1901 teachers employed and paid by the county Government. To ensure viable ECDE policies are developed, the County Government will work closely with the National Government to ensure that the stand alone ECDE centres are brought into the reporting framework. | **b**. Kakamega County Water and Sanitation Company has been established for effective provision of water and sanitation services in Kakamega including providing access to safe drinking water through construction, rehabilitation and augmentation of existing water supply schemes, drilling, and equipping boreholes. **c**. Increase in households practicing proper hygiene and improved hygiene in public places. **d** **& e**. Control of air pollution, noise pollution and ensure that the department will ensure that major projects in the county undergo environmental impact assessments/ audits and that the environmental monitoring plans are fully implemented. **g**. The construction of Gender Based Violence rescue centres, equipped child rescue centres and improved social welfare to reduce GBV cases. **h**. There are several social net programs carried out in the County including the Shelter improvement program where the county government constructs housing units for the vulnerable. So far, 720 housing units have been constructed for the Vulnerable people. **i.** Other social net programmes include financial and material support to Charitable Children institutions as well as Cash Transfers for vulnerable expectant and lactating mothers. |
| 12.Kericho | Not clear | Under ECDE, pupils’ enrolment as of 2017 comprised 23,094 boys and 22,526 girls giving a total of 45,620 pupils thus boys representing 50.6%.  There are over 1800 teachers teaching in these centres, with 935 employed by the county government on contract. | **a**. The contraceptive prevalence rate for the county is currently at 62% against the national figure of 58%. This is due to availability of family planning commodities, outreaches, and collaboration with partners. **b**. The immunization coverage stands at 61% which is below the expected national target of 90%. **d**. Prevention of Mother to Child transmission (PMTCT) is provided through the antenatal care (ANC) programs in selected facilities. There are about 813 pregnant women living with HIV who were provided with PMTCT services out of a total need of 917 yielding 89% PMTCT Coverage. **f**. Currently, pregnant women with 1^st^ attendance to ANC is 20,407 and those who attend at least 4 ANC visits is 9, 110. Deliveries conducted by skilled health attendants stands at 89.8%. | **a**. Through use of healthcare services and ECDs to identify and tackle child malnutrition the county will ensure that everyone can enjoy a safe, nutritious diet, all year round. **d**. Currently, 70% of the population receive nutritional supplements with a further 25% increase planned. **f & i**. The county will carry out feeding for ECDE School going children to address issues of malnutrition as currently there are 0.6% children under 5 who are stunting and 1.4% are underweight 0-59. **g**. There are also plans to increase children who are dewormed to 45%. | **d**. The county will carry out feeding programme for ECDE school children to address issues of malnutrition. **e &** **f**. Furnishing all ECDE centres built by the county in the last five years and construction of modern  child friendly classrooms and  equipping staffing. | **a**. The county plans to establish model ECDE centres to improved early childhood  learning. The county is responsible for ECDE that forms the foundation for education as such, it will continue to identify and tackle the challenges to access schools. **d**. Currently there is one library managed and run by the Kenya National Library Services hence the county plans to establish and promote libraries to encourage reading culture in the county.  **e**. Although great improvement has been made to increase access and quality of ECDE over the last five years, there is still need for improvement in the development of quality infrastructure and increase in the number of teachers for the same cadre of pupils. | **b & c**. Provision of safe and clean drinking water is a strategic intervention that the county promises to execute especially in water scarcity areas including sewer lines and septic tanks whereas majority of the populace uses pit latrines as well as improve hygiene for all.  There is need for a revolving fund for women and disabled as well as capacity building. **d**. Control of air pollution, noise pollution another public nuisance. **e**. Environmental pollution has contributed to loss of biodiversity and diminishing health and sanitation standards. **g**. Social protection in the county covers issues on gender in terms of gender violence. **h & i**. Social net programs in the county are all run and managed by the national government through the children department and social services including cash transfer program for the severely disabled, orphans and vulnerable children and children welfare services. The county through the department of social services network and support all the activities of children, disabled and gender in the county. |
| 13.Kiambu | Under 5 however pre-primary education is focused on age 3 to 5yrs | There are 491 ECDE centres in the county. The total number of ECDE teachers is 5370 with public centres having 1200 ECDE teachers. The teacher to pupil ratio is 1:28. The total enrolment for ECDE is 99,061 pupils, 33,336 in public ECDE comprising 17,071 males and 16,266 females while private ECDE centres have total enrolment of 65,725. The gross enrolment rate is 71.70% with completion rate retention and transition rate falling at 95%. There are 98 schools for pupils with special needs where 3163 pupils have been enrolled. | **a**. Total family planning attendances reached 72% of women of reproductive age receiving family planning. The country has a rapidly growing population with 24% being below 20 years, who puts great demands on provision of family planning service. **b**. The county had immunization coverage of 89% of children under one year who were fully immunized. There is still 11% of unreached children in the county due hard to reach areas such as slums, plantation worker’s children and inadequate supply of commodities. **c**. Rehabs, seminars and peer training for drug and substance abuse has been planned. **d**. Prevention of mother to child HIV transmission has improved with 100% of HIV positive pregnant mothers receiving preventive ARVs. **f**. 172,996 antenatal clients were attended in the last CIDP with a total number of skilled deliveries reaching 57,580. This has been accelerated by free maternity initiative. Skilled delivery is currently at 88.5%. **m**. Establishment of baby care for children with severe disabilities | **b**. 72% of children are currently exclusively breast fed with plans to improve this to 95%.  **c**. The county plans to provide sustainable livelihoods through household food and nutritional security to families. **d & g.** About 68% of children currently receive Vitamin A supplementation, and about 20,000 school children are dewormed. **f.** Growth monitoring revealed stunting level at 15.7%, wasting at 2.3% and underweight at 5.1%, while the national stunting level is at 37 26%, wasting at 4%, and underweight is at 11%. Improved survival rates of children by increasing the % of children exclusively breast feed. **i**. The county plans to establish  childcare and feeding programme to improve developmental health, learning and psychosocial wellbeing of ECDE going children. | **d**. Introduction of free school feeding program for ECDE going children increased the retention rate of ECDE going children as well as improved their health. The county plans to establish more feeding programmes to improve developmental health, learning and psychosocial wellbeing of ECDE going children. **e**. Including providing play, teaching and learning materials and aids to ECDE children and equipment to improve the quality of training and education. **f**. Under ECD programme, parents have been relieved of paying ECDE care givers by recruiting additional care givers and absorbing 1200 ECDE care givers. Procurement and distribution of teaching and learning materials to ECDE’s was done which included rulers, pencils, exercise books manila papers, crayons among others. | **a**. The document emphasises on the county government's commitment to offering quality education by employing more ECDE teachers, construction and renovation of ECDE classes and school feeding program for early childhood development, care and pre-primary education. **b**. Provide play and teaching and learning materials and aids to ECDE children. **d**. The county has initiated construction of two libraries at Lussengeti in Kikuyu and Karuri in Kiambaa sub counties however, the two projects are stalled, hence the need to revive them. **e**. The county government is fully committed to offering quality education by employing more ECDE teachers, constructing and renovating ECDE classes and pre-primary education. | **b**. Water policy development and management has been advocated to improve water and sanitation management as currently 50% of the population have access to clean and safe water and better sanitation services, with plans to increase access to 80% of the population. **c.** The county plans to ensure people have access to adequate and equitable sanitation and hygiene to end open defecation through construction of good drainage system to curb floods, provision of high standard hygiene facilities including toilets in market and public places to enhance good hygiene to residents. **d**. The county envisages providing non-motorised traffic lanes for the people as well as reduction of air pollution to road users. **e**. To enhance clean environment, the county plans to monitor and manage industry emissions that have led to air and water pollution as well as various agrochemicals in the water sources. **f**. There are also plans to establish recreational parks and to rehabilitate township public packs for people to relax. **g**. The county has gender-based violence bill to protect victims. **h & i**. The National Safety Net programme cash transfers have made positive impact in the lives of beneficiary households by improving their welfare and increasing their resilience. Cash Transfer for Orphans and Vulnerable Children was launched in 2004 |
| 14.Kilifi | Not clear | Unclear | **a**. The county plans to increase awareness on family planning by creating outreach programs, sensitisation on the importance of family planning and creation of awareness on the different methods of family planning. **b**. Also, to provide outreach immunization services, creating public awareness and provision of extensive immunization exercises. **c**. As well as creating awareness about alcohol and drug abuse. **f**. Equipping the existing maternal healthcare facilities to provide better and timely services. | **a & c**. As a result of poor diet and malnutrition issues the county plans on hiring Nutrition Officers to public health centres to create awareness on the importance of balance diet. **d**. Provision of micronutrient supplements at health facilities. **f & i**. Awareness creation and nutrition assessment. Treatment for malnutrition with nutritional supplements for stunting, provision of supplementary food in care facilities at the health units and employment of CHVs to provide awareness on malnutrition. | **d**. Establishment of ECD feeding programs such as provision of milk, provision of food for the children in ECD centres. **e.** Provision of game kits, playing kits and toys in all ECDEs. Improving Social development, child affairs and social safety net programmes. | **a & b**. The county currently lacks proper facilities in ECD centres including playgrounds hence the county plans to provide learning and teaching facilities in all ECD centres and to establish of playing grounds. **d**. Most communities do not have libraries hence the county plans to construction of a modern community library. **e**. There is a construction of modern ECDE facilities with ongoing employment of more teachers to administer the ECDE services. | **a**. Due to limited access to birth certificate registration services, the county plans to create awareness of people’s rights to access birth certificates. **b & c**. Construction of boreholes to provide safe and clean water as well as toilets in all schools improve sanitation and hygiene. **d** **& e**. Finding a suitable site for waste dumping to reduce events of air pollution and cholera outbreaks as well as provide education to people about conservation of environment. **g**. Gender mainstreaming programmes and rescue centre for Gender Based Violence clients are being established to support victims. **h & i**. Construction of three fully equipped rescue centres and sensitization campaigns on child protection as well as construction of a social welfare office at sub-county level with cash disbursement to Orphan and Vulnerable Children. |
| 15.Kirinyaga | Under 5 | Currently Kirinyaga has 198 public ECDE centres manned by 447 ECDE teachers under contract. The ECDE enrolment as at May 2017 was 15,851. This implies the teacher child ratio stand at 1:36. The ECDE centres are still allocated within the  public primary schools except for five (5) which are standalone ECDE centres and  feeder schools to nearby primary schools. | **a.** Contraceptive acceptance in the county is 66.3%, access to contraceptives is high due to services being offered free of charge in most government owned institutions. **b**. Child vaccination in the county is 98.3 percent while.  **c**. The county plans to strengthen the prevention and treatment of substance use including narcotic drug abuse and harmful use of alcohol. **d.** The proportion of Mother to Child Transmission of HIV had significantly dropped from 7.5% to 5.7% in 2017. **f**. Antenatal Care stands at 42%, which is higher than the national figure of 36%. That is a result of the increase in the health care facilities that offer antenatal care across the county and free access to skilled child delivery. | **a.** The county plans on improving maternal and childcare health services and nutrition standards.  **b & c**. Most mothers breastfeed their children during their first year coupled with constant supply of food. Young mothers and fathers are trained on breast feeding alongside centre construction. **f** **& i.** Malnutrition is not a big concern in the county as the proportions of stunting, underweight and acute malnutrition is below 2.5% among children below 5years. | **d**. For learners in ECDE and general population, the county plans to improve nutritional status of preschool which will help retention of children in school in relation to ECDE caregivers. **e**. Appropriate and safe play materials and equipment have been provided in existing centres and 20 ECDE Centres have also been provided with fixed play facilities. | **a & e**. The county has improved infrastructure and learning environment in ECDE centres as well as improved access, quality and equity of ECDE Services. There are trained 403 ECDE teachers on Child Counselling and Development of Teaching/  Learning Materials. **b**. The county has also provided 20 ECDE Centres with fixed play facilities. **d**. The county currently has one fully furnished library hence there are plans to construct more  community cultural  library to promote reading  culture particularly among  young people. | **b & c**. The water in the rivers has been harnessed through canals to provide safe water for domestic use as well as irrigation purposes. The piped schemes supply 51,515 households. There is no sewerage system in the entire county and the households with flush system construct their own septic tanks. About 90% of the households use a pit latrine, **d**. The county plans to control air pollution, noise pollution, other public nuisances and outdoor advertising. **e**. The sector will ensure that sustainable projects and programmes are initiated in the County and Environmental Impact Assessment will be done to keep the environment safe from pollution. **g**. Gender-based rescue centre is being built to support victims, with treatment and counselling facilities to reduce the number of domestic/  gender based violence. **h & i**. Social protection for people with disability with cash transfer programs which has been on pilot in the county and now it will be rolled out to cover the needy. |
| 16.Kisii | Under 4 | There are 2 Pre-School education including Nursery and Pre-Unit. 701 public ECDE centres and 378 registered private ECDE centres are in the County with a pupil enrolment of 124,991, representing 98.6% net enrolment rate. The County has initiated the construction of 315 classrooms in ECDE centres in a bid to improve the learning environment so as to reduce dropout rates and increase transition rates thereby enhancing the quality of learning and teaching in these centres. | **a**. Contraceptive prevalence stood at 62.8% compared to the national figure of 53.2% **b.** The immunization coverage rate in the County dropped from 97% to 57.3% in 2017, reflecting a decline due to prolonged nurses’ strike. **d**. There is currently reduced mother to child transmission of HIV and the county is adopting option B plus in PMTCT. **f & h**. There is antenatal care for all pregnant mothers and introduction of daily paediatric clinic, growth monitoring, health promotion and paediatric HIV screening. | **a & d**. Macronutrient supplementation to eligible clients in TB & HIV clinics and increased Vitamin A supplementation for lactating mothers & under-fives, increase zinc supplementation in diarrheal cases, f. Growth monitoring and promotion, as the County has a few numbers of stunted children’s growths. **g**. Increase de-worming target from 2 years and **h.** there is need to sensitize communities on proper feeding methods. | **d**. The county plans to improve ECDE School feeding programme and number of ECDE centres enrolled for the feeding programme to improve nutrition among children. **e**. There are also plans to purchase and distribute playing equipment to ECD centres across the County. | **a**. The county has equipped ECD classrooms with educational and learning materials, **b**. purchased and distributed playing equipment to ECD centres across the County and **c.** partnered with a donor to distribute books to learning centres across the County. **d**. Currently, there exists one National library in Kisii Town, additionally, the County has constructed two libraries, but are not operational as they are yet to be stocked with relevant materials. **e.** There are 2 Pre-School education including Nursery and Pre-Unit. 701 public ECDE centres and 378 registered private ECDE centres are in the County. | **b.** The county aims to achieve universal and equitable access to safe and affordable drinking water for all by 2030 by improving water quality and minimizing release of hazardous chemicals and materials and increasing recycling and safe reuse. Decent sanitation includes appropriate **c**. hygiene awareness and behaviour as well as acceptable, affordable, and sustainable sanitation services which is crucial for the health and wellbeing of people. **d**. Reducing the number of deaths and illnesses from hazardous chemicals and air, water, and soil pollution and contamination. **e.** Provision of clean environment through proper waste management. **h.** The county plans to establish structures and systems to support child protection, implement child protection policies, programmes, and projects, Establish children officer/child protection desk in the Sub Counties. **i**. There is need to enhance the cash transfer programme to ensure that the needs of the registered children are taken care of. |
| 17.Kitui | Under 5 | There is a total of 1850 ECDE centres in the county with total enrolment of 1826 children: 1518 enrolled in public ECDE centres and 308 enrolled in registered private ECDE centres as of 2014, with a pupil enrolment of 124,991, representing 98.6% net enrolment rate. | **a**. Contraceptive acceptance in the county stands at 58%. The low rate is attributed to the prevailing culture, traditions, and lack of awareness within the community which promotes negative attitudes towards the uptake of contraceptives. **b**. The immunization is generally low at 63 % compared to the National status 83.5 %. The trend has worsened from 75% in 2012. To reverse the trend and improve immunization coverage to 80%, the county is increasing outreach services and the number of immunisation centres, as well as ensuring adequate vaccine supply. **c**. There are plans to develop a workplace policy and public policy on alcohol, drugs and substance abuse. **d.** The county plans to reduce mother to child transmission of HIV by monitoring the percentage of pregnant women counselled and tested for HIV and percentage of HIV+ pregnant mothers receiving preventive ARVs. **f.** The percentage of mothers attending 4 antenatal clinics is 52.5%, compared to the country average of 52%. The proportion of mothers delivering under the care of skilled health workers is only at 27.6%, which is below the national average of 42%. Provision of reproductive health services needs to be brought closer to the people by setting up at least one well equipped and staffed maternity unit at the ward level. | **a & c.** Only 76.9% of the caregivers fed their children more than three times in a day indicating inadequate complementary feeding. Improve nutrition status of children by increasing the proportion of pregnant women receiving Iron Folate at least for 90 days. **b.** Exclusive Breast Feeding for six months stands at 45% while eHealth 85.4% had initiated breastfeeding within the first hour after delivery. **d**. Increase the proportion of children aged 6 - 59 months receiving vitamins A supplements twice a year. **e.** Expand Kitchen Garden programs for affordable local grown foods and Reduce Prevalence of Malnutrition among the under-five in the County. **f.** Wasting rates among children less than five years are at 4.6%. Stunting is currently at 38.2%, which is way above the national average of 26%. Prevalence of underweight is 20.7% as compared to the national average of 16%. **g**. The county plans to increase deworming programme for ECDE learners and increase the deworming programs and the number of deworming sessions conducted. **i.** The county plans to expand kitchen garden programs for affordable local grown foods and reduce prevalence of malnutrition among the under-five in the County. | **d**. The household dietary diversity score was 4.7%, with 95.2% of households accessing food by purchasing, which implies that many households have limited options of livelihoods in the wake of low household incomes and high prices of staple food. | **a.** The county plans a construction, equipping and operationalisation of ECDE centres to enhance quality Early Learning Education and support working teams to deliver effective services. **d**. The Modern Library in Kitui town has been completed with ICT Centre to assist youth in education, training, and research. **e**. There are 1850 ECDE centres in the county with total enrolment of 1826 children: 1518 enrolled in public ECDE centres and 308 enrolled in registered private ECDE centres. | **b & c**. The county is committed to provision of safe water, proper saste disposal, functional sewer systems and timely repair of leakages and enforcement of hygiene laws. **d & e**. Reduced indoor air pollution for the rural communities and to manage waste disposal and formulation of policies to improve quality of life through provision of clean environment. **g**. Sexual and Gender-based Violence (SGBV) programmes and creation of awareness on SGBV as well as available helplines/shelters have been created to empower victims and the linkage of SGBV survivors to health and legal services. **h. & i.** The National Government supports three cash transfer programmes in Kitui County, namely Orphans and Vulnerable Children and Persons with severe disabilities. There is also enhanced access to child protection and childcare services within the County, with strategy is to advocate for the guidelines for day care centres. |
| 18.Kisumu | Under 4 | The total number of children enrolled in public and private ECDE as of 2017 stood at 57,893 and 58,803 respectively against with 1,344 trained teachers in public and 2,118 in private ECDE centres. There is a total of 2,752 children with special needs (visual, hearing and speech) out of which 1,954 of these children are in public schools.  There are 483 public ECDE centres of which 217 are on permanent building structures, 237 are on temporary structures while 29 fall under others. 187 of these centres have tap water connections, 136 have wells and 59 depend on water vendors while 60 centres use other water source. | **b**. The County's immunization coverage currently stands at 84% above the national figure of 69%. **c**. The county has created public education awareness/ sensitization and has set up rehabilitation centres to reduce of alcohol and drug intake and abuse. **f.** All pregnant women are provided with Intermittent Presumptive Treatment with plans put in place to curb the infant mortality rate of 54/1000 live births which is above the national rate of 39/1000 live births. | **a**. The county aims to achieve improved maternal and child feeding practices as well as improved food security and ECDE feeding. **b**. 76% of children are exclusively breastfed, compared to the WHO target of 50% by 2030. Exclusive breastfeeding is being encouraged and plans to increase the number of established breastfeeding resource centres. **d & g**. Less than half (43.6%) of children aged 6-59 months receive Vitamin A capsules compared to the national average of 72% including deworming campaigns, with pregnant women receiving IFAS for 270 days. **f**. Growth monitoring indicate that 2.2% of children <5 years are wasted, 6.6% are underweight and 18% are stunted. These outcomes are closely linked to poor child feeding practices and the quality of children’s diets. **i**. There are facilities stocked with adequate therapeutic and supplementary feeds to reduce stunting among <5 years. | **d.** The county aims to achieve improved maternal and child feeding practices as well as improved food security and ECDE feeding. Poor nutrition among children under five is associated with long term deprivation and poor health resulting in reduced performance productivity later in life. **e**. The county plans to equip ECDE centres with play equipment as well as learning and teaching materials. | **a**. ECDE centres are being equipped to increase enrolment, improve learning environment supported with learning and teaching materials. **b**. The county plans to equip ECDE centres with play equipment. **d**. Existence of fully equipped centres with computers and library materials. **e**. There are 483 public ECDE centres of which 217 are on permanent building structures, 237 are on temporary structures while 29 fall under others. | **b & c**. The county plans to increase access to safe water supply in rural areas as well as promote and scale up rainwater harvesting at household, public institutions, community levels and to improve sanitation and hygiene. **d**. Controlled industrial air pollution and reduced levels of noise pollution. **e**. Healthy population living in clean environment. **g.** There are plans to construct and equip gender-based violence (GBV) recovery centre to enhance security and protection for GBV survivors. **h & i**. The county is improving the livelihood of vulnerable groups specifically the orphans and vulnerable children, and persons with disability by the establishment of Consolidated Social Protection Fund which provide Cash Transfer to the severely Disabled Persons and the Urban Food Subsidy in addition to the Cash Transfer programme to Orphans and Vulnerable Children. |
| 19.Kwale | Under 5 | The County has a total of 1,072 ECD centres spread evenly in the county with 820 being public and 252 being private. 238 new ECDE  Centres have also been established. The total enrolment stands at 77,067 pupils comprising of 39,580 boys and 37,487 girls.  There is a total of  2,087 ECDE teachers in the County with 784 employed by the County Government and 1,333 unemployed. The teacher/pupil ratio is at 1:37 and the average ECD attendance age is 4.5 years. | **a**. Contraceptive prevalence among women in the reproductive age group stands at 45% compared to 53% nationally. There is need to have male involvement to broaden the scope of methods in family planning. **b**. Immunization coverage is high above the recommended 80% immunization coverage for all the three vaccinations due to effective roll out in health facilities, outreach programmes and immunization campaigns. **d**. There is increase in HIV/AIDS Prevention and Control through increasing the Percentage of HIV pregnant mothers on ARVs. **f.** The percentage of women aged 15-49 who attended at least 4 Antenatal Clinic visits stood at 54%. The proportion of mothers who delivered in health facilities was 67%. | **f**. The predominant form of manifestation of malnutrition in the county are stunting, underweight and acute malnutrition accounting for the 29%, 21% and 6% respectively. The problem of malnutrition is attributed to poor diversification of food sources consumed in households. **i.** Proper nutrition interventions should be put in place to address the cases of malnutrition observed in all the livelihood areas involving concerted efforts together with the community and households in Kwale County. | **d**. The government aims at promoting accessible, quality, and responsive services for all and reduce malnutrition, including stunting and wasting in children under 5 years of age. **e**. Installation of outdoor  playing equipment in each  ECDE centre and establishment of an ECD training school for  teachers. | **a.** The County sees education as a critical component in development right from preschool to the highest levels of education, as such it prioritised the universalisation of ECD in the county, the engagement of competent staff and availing the materials and tools necessary to advance ECD. **d.** Kwale County has one public library under the Kenya national library to promote reading culture and improve literacy levels. **e.** The gross enrolment of ECD children stands at 83.7% and a net enrolment 81.2%. The county plans to promote early childhood education, ensure inclusive and equitable quality education, and promote lifelong learning opportunities for all as well as provision of teaching and learning materials to the centres. | **b**. The county plans to improve the availability and supply of clean and treated water by constructing large water Dams and Pans, drilling of boreholes, pipe reticulation, water testing and treatment. **e**. There are plans to also improve environmental protection and management including sustainable waste management. **g**. Sensitization of county departments to incorporate gender issues in planning, budgeting, and implementation of their programmes. **i.** Percentage increase in amount of support/ loans Number of disabled persons provided with assistive devices. |
| 20.Laikipia | Not clear | As of 2012, the county had 518 ECD centres with a total enrolment of 23,160 pupils. The number of ECD teachers is 881 with a teacher pupil ratio of 1:26. The gross enrolment rate stands at 59 per cent with a completion rate of 95%. | **a**. The contraceptives acceptance uptake is 50.5%, despite the high acceptance rate, just over one third of the married women use modern methods of family planning. **b**. Immunization rate for children under 5 stood at 66.4% on average. Vaccination against BCG and measles coverage stands at 76.9% and 64.3% respectively. **c**. Laikipia County Alcoholic Drink Control Act has been implemented to control drug and substance abuse. **d**. Elimination of mother to child transmission stood at 99%. **f.** The pattern of growth in the County calls for appropriate planning for the delivery of antenatal and postnatal healthcare services as the percentage of expectant mothers visiting health facilities at least twice is estimated at 93.7% while those attending postnatal care recorded 80% with 39.9% first visit and 32.3% revisit. | **a**. The county plans to improve maternal and child nutrition as nutrition remains a challenge in the county. **d.** Vitamin A supplementation for 6 to 11 months stands at 66.1%, 12 to 59 months at 16.9% and an average of 50.9%. Pregnant women supplemented with iron Folic Acid between 90-180 days stands at 30.2%. **f**. The estimated prevalence of children underweight is 20.1% out of which 5.3% are severely underweight. The prevalence of stunted growth is 25.1 out of 5.9 are classified as severely stunted whereas prevalence of wasted was 4.4%. **h**. The causes of malnutrition among under-fives are poor dietary diversity, suboptimal infant and young child feeding practices, poor hygiene and sanitation and low access to essential nutrition services. | **d.** There are joint campaigns, sensitizations on school feeding program. **f**. Child caregivers need additional training and skills in public relations and speaking skills, bookkeeping, managerial skills, leadership skills group dynamics and paramedic skills to improve the care that they extend to children. These areas are paramount in extending responsive caregiving. | **a**. The County sees education as a critical component in development right from preschool to the highest levels of education, as such it prioritized the universalization of ECD in the county, the engagement of competent staff and availing the materials. **d**. There are two public community libraries, to encourage reading and literacy. **e**. ECDE early learning is prioritized where the priorities highlighted include infrastructure development, quality assurance and standards, and an increase in the number of teachers. | **b**. The Laikipia County has established a water development that is promoting access to safe and reliable water and sanitation, increased clean and safe water in the county, increase of access to the population with sewerage services. **d**. Control of air and noise pollution and outdoor advertising. **h & i.** The main programmes on social safety are cash transfers to persons living with disabilities, orphans and vulnerable children. |
| 21.Lamu | Under 5 | There are 203 ECDE centres, 63 of which were constructed by the County. So far, there are about 13,000 pupils enrolled in all the ECDE centres. There are 478 teachers giving a teacher to pupil ratio of 1:27. There are also 3 special needs pre-schools: 2 for Mentally Handicapped; and 1 for Hearing Impairment | **a**. The number of women using modern family planning methods is 40%, which is very low when compared to the national average of 53%. The use of long-term methods of family planning is also low and efforts to increase acceptance are required. **b**. The national end target for fully immunized children is 90% but the County has observed a declining trend from 92% of infants being fully immunized to 84% and 77% by 2017. This has called for renewed efforts to improve coverage. **d**. Percentage of infants born to HIV+ mothers who are infected. **f**. Access to antenatal care is 96%, which is like the national average. However, only 62% of pregnant women attend the recommended 4 antenatal clinic visits, with a national coverage of 58% reflecting low utilization of the services. Deliveries by skilled birth attendant’s coverage stands at 47% against a national average of 62 %. | **c**. Provide meals and nutrition to ECDE for improvement of health. **f**. As of 2017, 2.4% of children aged below five years are overweight with an estimated stunting rate for children below five years of 29%, wasting rate of 4% and 11% were underweight compared to the national average of 26%, 4% and 11% respectively. | **d**. Provide meals and nutrition to ECDE for improvement of health. **e**. The county has also purchased and distributed chairs, tables, bookshelves, and outdoor play equipment for the ECDE centres. **f.** Provision of learning and teaching resources for ECDE centres. | **a**. Efforts are being made in ensuring that ECDE facilities are well equipped with adequate teachers and learning materials for effective delivery of services. **b** **& c**. The county has also purchased and distributed chairs, tables, bookshelves, and outdoor play equipment for the ECDE centres. **d**. There are plans to construct and equip ECDE centres with a school library **e**. There are 203 ECDE centres with 3 special needs pre-schools for Mentally Handicapped and Hearing Impairment. | **b.** The county plans to increase safe water access to all as well as construct sewerage systems to promote sanitation services in the respective areas. Ensuring access to clean water for domestic use and consumption.  **d.** The county will control noise and air pollution. **h & i.** Ensuring access of County Social Support (Women Empowerment Fund, Fund and Disability Fund) and loans for women, youth, and persons with disabilities. |
| 22.Machakos | Under 5 | Overall, the County has 1,468 ECD centres with a population of 55,121 children. This population is projected at 189,779 in 2018 then to 201,111 and 213,121 in the year 2020 and 2022 respectively. Measures should be put in place to cater for the anticipated increase in number of teachers, teaching and learning facilities for ECD centres. | **a**. Contraceptive prevalence rate for women between 15 – 49 years using modern family planning methods stands at 68%. There is a need for more campaigns to ensure people considers using various methods of family planning. **b**. The  immunization coverage stands at 70.4%, which is below WHO standards of 85%. It is also considerably lower than the baseline which was 77.7%. **c**. There is awareness creation on the adverse effects of alcohol and substance abuse. **d**. The county will monitor the proportion of pregnant mothers tested for HIV and scale up male engagement in prevention of mother-to-child transmission. **f.** Measures would be scaled up to cater for anticipated increase in antenatal care (ANC) and post-natal maternity services as well as Increased utilization of ANC services by monitoring the percentage of women attending 4 ANC visits and deliveries conducted by skilled attendants. | **b & d**. Promotion of exclusive breast feeding for the first six months and increase supply of therapeutic and supplementary commodities. **f**. The predominant forms of malnutrition in the County are stunted children under five years, which accounts for 27% followed by underweight children, wasted children and overweight children. **i**. The county plans to reduce prevalence of all forms of malnutrition among children aged 0 − 59 months by increasing the percentage of facilities carrying out routine growth monitoring services among children aged 0 − 59 months and increase the number of children screening for malnutrition. | **d**. Promotion of exclusive breast feeding for the first six months and increase supply of therapeutic and supplementary commodities including  increasing feeding programs in schools for ECDE pupils  **e**. Support acquisition of school playgrounds and provide social support and safeguarding cultural heritage. | **a**. There are 1,468 ECDE centres with enrolment of 57,581 children and 2,115 ECDE teachers. **d**. The County has two information documentation centres which also act as libraries. Moreover, there is a library and ICT centre located at the Machakos Youth and Children Centre as well. **e.** Pre-school Education is fundamental for child development and literacy improvement in the County. The County will therefore need to employ more pre-primary school teachers. | **b**. Accessibility to clean and safe water for both domestic and industrial use had been provided with overall distance to the nearest water source significantly decreasing from 6 km to 0.5 km. Irrigation and Sanitation are promoting sanitation and supporting water resource management to enhance availability and accessibility to safe and clean water. **c.** There is improvement in sanitation and hygiene conditions. **d.** Prevention of air pollution resulting from emissions from factories and industries which causes airborne related illnesses to the part of population that live in these areas. **e**. The county plans the provision of clean energy that promotes clean environment. **f.** Most of the County's urban centres have inadequate recreational facilities. Machakos People’s Park in Machakos Town which is open to the public for range of recreational activities such as boat ridding, children’s games, events, zip-line, filming. **g**. Prevention and response to gender-based violence with sensitization and awareness creation on gender-based issues. **h & i.** The national government offers social safety net programmes in the County namely Cash Transfer for Orphans and Vulnerable Children. |
| 23.Mandera | Under 5 | The County Government has invested in the ECDE sector, which has 259 public ECDE centres with an enrolment of 34,341 children, of which 19,066 are boys and 15,275 are girls as of 2017. The county has 260 trained and 260 untrained ECDE teachers. This is magnificent achievement since the  County had only 4 ECDE teachers employed by the former Mandera County Council before devolution. | **a**. The contraceptive prevalence rate improved to 2% from 1.9%, which will have a minimal impact on overall population growth rates in the county. Injectable contraceptives are the most preferred family planning methods in the County, followed by Implants. **b**. The immunisation coverage for the county stood at 32%. Most of the functional health facilities provide immunisation services. The county receives vaccines from the Nairobi depot on quarterly basis, which is posing a logistical challenge to the immunization campaign. **c**. The Mandera County Drugs and Substance Abuse Committee research into drugs and substance abuse challenges including training and sensitizing women against violence, drug, and substance abuse. **d**. There is implementation of mobile prevention of mother-to-child transmission services including increasing the percentage of HIV+ pregnant mothers receiving  ARVs. **f**. Many mothers now trust public health facilities, which has increased the skilled birth attendance to 34%, while 4th antenatal care visit stands at 36%. | **f.** In terms of height-for-age, 31.8% of children are chronically undernourished, in terms of weight-for-age, 41.2% of children are underweight, which indicates that nutritional situation in the County, is very critical across all livelihood zones. Growth monitoring programme and health nutrition in ECDE centres including **g**. deworming of all ECD Children. **i**. Reduce malnutrition status of children by formulating and adopting nutrition policies specific to Mandera county and customization of other relevant national policies including provision of feeding programme and nutrition service. | **d**. Improving health nutrition and feeding programme for ECDE including provision of feeding programme and  nutrition service. **e & f** Provisions of adequate, instructional and play/ learning materials for ECDE children to improve the learning environment and access to conducive learning including assessing the number of indoor teaching and learning materials as well as creation of inclusive learner friendly classrooms. | **a & b.** To fully develop psycho-motor skills of the minors, the county plan to gradually equip all the ECDE centres with the  relevant training and playing tools such as see-saws, balls, sandpits, swings etc. This will help improve children’s social skills, integration ability, cognitive and motor development. **d**. Development of library services to promote reading culture. **e**. The county has already established an ECDE college, which has commenced training ECDE teachers. The investment in the ECDE sector has greatly improved both ECDE and pre-primary school enrolment. | **b**. Water is a basic right, and the county’s dream is to achieve improved water sources with access to safe drinking water, which is critical for the health of individuals and households including sanitation facilities. **c**. There are plans to improve hygiene, cleanliness, and reduced disease  transmission. **d**. Control of air pollution, noise pollution and other public nuisances and  outdoor advertising. **e**. Maintaining clean environment free from pollution caused by emission is crucial. **f**. Outdoor play equipment has been provided including construction of playgrounds. **g**. Reducing gender-based violence. **h & i**. The county government have provided grant to 5 orphanage centres and is in the process of developing cash transfers to the vulnerable in the county. |
| 24.Makueni | Under 5 | The county has a total of 1,300 ECDE centres served by 987 teachers with a teacher to pupil ratio of 1:25. The total enrolment is over 48,000 children with a retention rate of 94.4% and transition rate of 83%. | **a**. The county is promoting reproductive maternal and child health by increasing family planning services to increase contraceptive uptake from the current 65% to 72%. **b**. There are plans to increase immunization coverage from the current 85% to 95%. **c**. There is reduction in drug and substance abuse incidences. **f**. There are also plans to increase the percentage of pregnant women attending antenatal care from 40% to 60% as well as the percentage of births attended by skilled birth attendant. | **g.** The county plans to promote medical camps outreaches and deworming campaigns. | **d**. The county plans to intensify school feeding programme to improve children nutrition targeting 250,000 ECDE pupils. **f**. There are also plans to recruit additional ECDE teachers including skilled staff in handling children with disabilities, building capacity of ECDE teachers and provision of modern equipment and learning materials in ECDE centres. | **a**. Adoption and implementation of the national ECDE policy is underway including developing a day care policy to regulate and standardize day care centres. **d**. The county plans to rehabilitate 3 existing community libraries and construct 2 model libraries to enhance access to educational and informational materials by the public. **e**. There are plans for construction or upgrading of one model ECDE in each of the 30 wards including provision of modern equipment and learning materials in ECDE centres, recruitment of additional ECDE teachers including skilled staff in handling children with disabilities. | **b & c**. The county is increasing availability and access to safe water within 2 km by constructing 6 mega dams complete with treatment plants, storage tanks and distribution networks including accelerating performance, improvement, and integration of intervention approaches in water, sanitation and hygiene. **e**. There are plans to create a just, all-inclusive, and cohesive society enjoying equitable social development in a clean and secure environment. **g**. There is monitoring of the proportion of county budget allocated to specific gender empowerment programmes and rate of sexual gender-based violence. **i**. There is enhancement of child welfare and protection of eligible households with OVCs receiving cash transfer |
| 25.Marsabit | Under 5 | The County has 252 public ECDE centres and 64 private ones. The ECDE enrolments are estimated at 19,239 while the total number of ECDE teachers are 413. The teacher pupil ratio in the pre-primary school is 1:29. The total enrolment in the public and private ECDEs is 16,005. The pre-primary retention rate is 99% with a drop-out rate of 0.2% while the transition rate is 99%. | **a**. Contraceptive acceptance is low at 8.3% due to cultural and religious beliefs, and the population that delivers in delivery points amounts to 76.2%. **b**. Immunization coverage in the county is at 66.6%. **c**. There are several public awareness raising meetings on smoking and drug use and substance abuse. **d**. The county plans to increase capacity of healthcare workers on HIV services including prevention of mother-to-child transmission of HIV. **f**. ANC coverage is at 42.8% and skilled deliveries is at 25%, stunted growth is at 26.5%, 30% of children are underweight. | **a.** Campaigns are being conducted to improve Maternal infant and young child feeding. **f**. In the county, 31% of the children below five years are malnourished while 26.5% are stunted. **i**. Efforts to improve the situation by both the government and NGOs include provision of food supplements and promoting income generating activities to vulnerable groups. | **d**. There are provision of ECDE meals to improve nutrient and enhance enrolment in schools. **e.** There is also provision of ECDE learning and teaching materials/outdoor and indoor play materials to improve learning outcomes for ECDE learners and enhance learning through play activities. **f**. Generally, the county is responsible for ECD that form the foundation for education. | **a & b.** There are provision of ECDE learning and teaching materials/ outdoor and indoor play materials to improve learning outcomes for ECDE learners and enhance learning through play activities. **d.** The county is building and equipping modern assembly library with shelves and books and computers. **e.** The ECDE enrolments in the county are estimated at 19,239 while the total number of ECDE teachers are 413. The pre-primary retention rate is 99% with a drop-out rate of 0.2 per cent while the transition rate is 99%. | **b.** The county is providing access to safe water and sanitation services with clean and secure sustainably managed environment and natural resources conducive for county prosperity. **c & e**. The sector has a mission to effectively promote, conserve, protect and sustainably manage the environment and natural resources including hygiene related diseases/illnesses and to enhanced food handling to improved hygiene. **d**. Control of air pollution, noise pollution, other public nuisances and outdoor advertising. **g**. Cross-sectoral efforts and campaign are being made against gender-based violence (GBV) reporting of GBV cases as well as gender mainstreaming in sector activities and legal support to victims of GBV. **h.** The department supported orphan and vulnerable children at Fatima and Marsabit Children homes by buying food and detergents worthy Ksh.500, 000. **i.** The national government is currently supporting 4000 households under cash transfer programme. Specific social protection programmes are supported by the county, including the cash transfer programme with 750 beneficiaries. |
| 26.Meru | 4 to 5 | There are 65,396 children enrolled in public pre-primary schools in the county which constitutes 32,080 male and 33,316 female. This number is enrolled in the 773 ECDE centres. The total number of ECD teachers is 1698 making the teacher child ratio to be 1:50 which is higher than the recommended ratio of 1:25. | **a**. Access to family planning has been increased to all women of reproductive age receiving family planning commodities in every subcounty within Meru. **b**. The county plans to improve immunization coverage to 90% by integrating community outreaches in religious organisations and public barazas encouraging women to take their children for immunization. **c.** There are plans to minimize health risk factors through behaviour change of smoking, drug and substance use. **d.** Prevention of mother-to-child transmission services has been established in all sub counties in Meru and there is a call to support elimination of mother-to-child transmission of HIV.  **f**. There are plans to increase mothers attending 4 ANC visits by 100% and encouraging more pregnant women to go for 4 ANC visits and community messages targeting women aimed at reducing early pregnancies and ensuring 2-year gap between births. | **c & e**. Awareness creation on behaviour and lifestyle change and nutrition screening, food fortification and food supplementary. **d & g**. Provision of Vitamin A and nutritional supplementation as well as deworming. **f.** Providing meals and nutrition to ECDE for improvement of health and monitoring Stunting Rate (Under 5) of children (12-59 months) and dewormed at least once a year. | **d**. Provide meals and nutrition to ECDE for improvement of health and monitoring stunting rate. **e.** Provision of play and rest materials. **f**. Employment of more ECDE teachers and construction of ECDE classrooms as well as procurement of ECDE learning materials to ensure conducive learning environment and enhance retention. | **a & e**. Development Education for improvement of health and employment of more ECDE teachers with construction of ECDE classrooms. There are plans for procurement of ECDE learning materials to ensure conducive learning environment and enhance retention. **d.** The county plans to build and stock a library and resource centre. | **b**. The number of households with access to piped water stands at 25,212 while household with access to potable water is 7,418, which contrasts sharply with the supply of water as only 2% of the population has access to piped water. The major sanitation facilities are pit latrines which are used by over 69% of the population. **c.** Improved sanitation and hygiene facilities in the rural and urban areas have been established. **d**. The county plans to prevent pollution of environment by biomedical wastes including water pollution. **e**. Enforcement of environmental regulations to curb pollution including radiation, toxic and hazardous substances management. **g**. To reduce Gender Based Violence through advocacy and creating an environment that is free from gender-based violence. |
| 27.Migori | 4 to 5 | The County had 816 ECD centres, 670 and 1174 ECDE teachers were employed by the county government  and community respectively as of 2017. Total enrolment stood at 109, 990 with a gender parity of  1:1pupils over the same period. The Teacher –Pupil ratio and literacy levels stood at 1:60 and 87.6% respectively. | **a.** The uptake of family planning in the county is 54.5%, which is mainly attributed to differing perceptions among spouses on contraceptive use, misconception and fears of side effects associated with use of contraceptives, cultural practices like polygamy and low access to contraceptive services. **b**. Immunization coverage is generally lower than the national average. This is mainly due to low literacy levels, lack of knowledge on the importance of immunization, long distances to the nearest health facility and births outside the health facilities. **c**. Reduced cases of drug and substance abuse. **d.** There is availability of HIV services and adequate prevention of mother-to-child transmission services. **f**. There is cumulative increase of pregnant women attending 4 antenatal care visits and increase of deliveries conducted by skilled attendant. | **b & d**. There was an increase in the number of children (0-5 months) on exclusive breast feeding by 20% and a 90% achievement on Vitamin A supplement provision twice a year. There was increase in the percentage of pregnant women receiving iron foliate. **f**. Stunted growth was reduced by 8.6%, stunting, wasting and underweight are key indicators of the nutritional status among children. **g**. 84% of school aged children per year were dewormed. | **d**. The county plans to establish and support feeding program in all ECD centres. **e & f.** Employment of sufficient trained ECDE teachers, provision of sufficient teaching and learning aids, improving the learner to facilitators ratio to the national standard of 1:40, developing educational delivery standards and strengthening teachers’ supervision. | **a & e**. The county is supporting ECDE centres through construction of more classrooms and more teachers, provision of sufficient teaching and learning aids, improving the learner to facilitators  ratio to the national standard of 1:40, developing educational delivery standards and strengthening teachers’ supervision. **d**. During the current plan period, the county in collaboration with development partners shall establish one public library and equip the existing information documentation centres. | **b & c**. To increase access to clean water, 21 water pans and 16 shallow wells were constructed, 4 existing water supply systems and 6 water pans rehabilitated, 268 water tanks procured and supplied to schools and hospitals, 25 water springs protected and 10 boreholes drilled and equipped. There is construction of VIP and modern water -borne toilets to improve sanitation and hygiene. **d.** Supporting efforts to develop and adopt improved cook-stoves to reduce indoor air pollution, and measures to increase sustainable biomass production. **e**. To improve on the living environments, priority will be given to slum upgrading projects and programmes in line with the SDG. **g**. Act, establishment of gender-based violence recovery centres, expanding child protection centres and advocacy for more child friendly justice system **h.** Among the interventions prioritized includes school feeding program, establishment of child support centres, child protection and rehabilitation, alternative family care services and **i.** cash transfer program where over 50,000 OVC are targeted for support during the current plan period. |
| 28.Mombasa | Under 1 & 4 | There are 770 ECDE centres within the county, 85 public and 685 private centres with a total enrolment of 47,867 pupils and 1,714 teachers. The school feeding programme has also contributed to high enrolment in the past and is expected to have a similar impact in the future if sustained. | **a**. Contraceptive prevalence among women in the reproductive age stands at 58% compared to 53% nationally. The unmet need for family planning amongst the urban poor remains a big challenge due to the question of commodity accessibility and a­ffordability. **b**. 94.8% (12–23 months) of children are fully vaccinated. **f**. 98% of pregnant mothers attend Ante-Natal Clinic and 89% of mothers delivered in health facilities while 21.6% delivered at home and 1.4% at other unspecified places. | **c**. 8 ECDE centres have been constructed across the 6 sub-counties and have provided the milk feeding program to the children in ECDE. **f**. 34% of children under five were considered malnourished: stunting, 11.2% wasting and 28.5% underweight respectively and there is need for the county to put in place measures to improve nutrition in women and children. **g**. Monitor the percentage of school age children dewormed. | **d.** Formulation and implementation of school feeding program in all ECDE centres. **e & f**. There is a construction & rehabilitation of ECDE centres including increasing the number of ECDE centres supplied with teaching & learning materials, and arts & play equipment. Provision of child-friendly schools, quality delivery of ECDE curriculum and improved learning environment. | **a**. There is a construction & rehabilitation of ECDE centres including increasing the number of ECDE centres supplied with teaching & learning materials, and arts & play equipment. Provision of child-friendly schools, quality delivery of ECDE curriculum and improved learning environment. **b**. Supplying each ECDE with teaching & learning materials, and arts & play equipment. **d.** Building mobile book facilities with library. **e.** Establishment and operationalization of childcare centres and construction of health facilities, among other interventions, have been planned to improve on accessibility, affordability and availability of services. | **b & c**. Providing clean and safe water to all households and establishing community units manned by community health workers to address sanitation, hygiene and health issues are among the county’s priorities. **d**. Control of air pollution, noise pollution, other public nuisances and outdoor advertising. **e**. Development of a county environment policy; environmental protection, conservation and compliance. **g.** Initiate friendly gender-based violence and rescue centres for victims to get counselling services. **h**. A social protection policy is being developed to ensure equal opportunity for the vulnerable, promotion and protection of children and children rights. **i.** The county plans to upscale cash transfer to the needy and to enhance urban food subsidy programme. |
| 29.Muranga | Unclear | The county has 1,000 ECD centres with total enrolment of 47,960 pupils and 1,503 teachers. The teacher/pupil ratio is 1:32. | **a**. The County has high usage of family planning services especially in urban areas as about 86% of urban population has access to family planning services compared to 33% of the rural population. **b.** The immunization coverage stands at 92%. Efforts are being made to ensure that all children are immunized against preventive diseases. **c.** There is increased awareness on dangers of substance and drug abuse. **d**. Prevent and manage HIV /AIDS through promoting preventing mother to child transmission and safe use of medical instruments. **f**. There are concerted efforts to increase skilled delivery and 4th ANC uptake and reduce maternal mortalities. Antenatal attendance first visit was at 62% while the recommended fourth visit was 27%. | **c**. County will institute a feeding program for ECDE to promote ECD school going children getting a packet of milk per week in addition to all 32,000 ECD pupils getting nutritious lunch in ECD centres provided by the county government. **d & g**. Deworming  children and provision of Vitamin A supplementation. **f**. 5% of children under five years are stunted and wasted. **i.** However, the medical personnel have been intensifying their effort to ensure that these figures come down. | **d.** The county is providing all 32,000 ECD pupils nutritious lunch in ECD centres. **f.** Teacher Refresher courses on new ECD curriculum and provision of ECDE teaching and learning materials have been implemented. | **a & e.** The county government is fully committed to offering quality education by employing more ECDE teachers, construction and renovation of ECDE classes and school feeding program for early childhood development and care including provision of ECDE teaching and learning materials. **d**. Equip libraries and the centre with social cultural and other relevant reading material. | **b & c.** The county shall strive to achieve universal equitable access to affordable safe and clean drinking water to address water scarcity. It shall also ensure people have access to adequate and equitable sanitation and hygiene to end open defecation. **d.** Control of air pollution, noise pollution, other public nuisances and outdoor advertising. **e**. The county aims to promote a clean, secured and sustainably managed environment and natural resource conducive to county prosperity. **f.** There is zoning of parks, open spaces, play fields and urban parks for families. **h & i**. Social protection and improvement of livelihood among the marginalized and increase in the number of people covered under cash transfers are underway. |
| 30.Nairobi | Under 1 & 4 | The County has 211 public ECD centres, of which 21 are stand-alone ECDs while 190  are in main primary schools. The private ECDs are 344 in number. The total number of ECD  teachers are 413 with teacher: pupil ratio in the pre-primary school is 1:29. The total enrolment in the public ECDs is 12,019 with private ECDs being 182,618. The pre-primary retention  rate is 99% with a drop-out rate of 0.2% while the transition rate is 99%. | **a**. Contraceptive prevalence among women in the reproductive age group stands at 58% as compared to 53% nationally. The unmet need for family planning amongst the urban poor remains a big challenge due to the question of commodity accessibility and a­ffordability. **b.** 94.8% (12–23 months) of children are fully vaccinated. **f.** 98% of pregnant mothers attend Ante-Natal Clinic with 89% of mothers delivered in health facilities, while 21.6% delivered at home and 1.4% at other unspecified places. | **b**. Offering training & encouragement on exclusive breastfeeding up to the sixth months & ensuring that the children feed on a balanced diet is a priority for the county. **e**. There are sample food products for fortification. **f**. Stunting levels for children under 5 years is 26% while 5% are stunted. The proportion of children under 5 who are underweight is 11% while overweight stands at 5%. **g.** Deworming programmes have been established for school children. | **d**. Encourage exclusive breastfeeding and school feeding programmes. **e & f.** The county is ensuring that the ECD programmes are well implemented by recruiting more ECD teachers, building more ECD centres and providing enough and appropriate learning and teaching materials. Provision of ECD centres with qualified teachers, desks and learning materials. | **a.** Ensuring that the ECD programme are well implemented by recruiting more ECD teachers, building more ECD centres and providing enough and appropriate learning and teaching materials. **d.** Promotion of library and information services as well as equipping the library with books. **e**. The county plans the provision of ECD centres with qualified teachers, desks and learning materials including construction of a school hall and equipping of a resource centre with reading materials. There are 211 public ECD centres and 344 private ECDs. | **b & c.** Provision of safe water by monitoring households with access to safe water, proper waste disposal, functional sewer systems and timely repair of leakages and enforcement of hygiene laws. **d**. The Environment sector seeks to control pollution through enforcement of air pollution regulations, procurement of air quality sampling kits. **e**. Creation of a healthier and cleaner physical environment. **f**. The County has challenges related to availability of open spaces and parks for recreational activities. **g.** The county seeks to establish a gender-based violence desk at the district level to address issues affecting men, women & children. **h & i**. Provision of social protection facilities for persons living with disabilities and enhancement of cash transfer. |
| 31.Nakuru | 0 to 5 | The County has 2,194 ECD centres of which 1,394 are privately owned while 830 ECD centres are public. The teacher pupil ratio in public ECDs stands at 1:33 whereas the ratio of private ECD centres is 1:20. Preschool enrolment is at 121,735 (boys 59987, girls 61748). The retention rate is at 92% while transition rate to primary school is at 96%. Efforts are in place to increase the coverage of school feeding programme to improve the retention and transition rates to 100%. | **a**. Currently, 57% of married women aged 15-49 use any modern method of contraception with a prevalent rate of any family planning method at 57%. The County will continue to invest in family planning services to improve uptake of family planning commodities. Overall facilities offering family planning services increased from 152 to 301. **b**. Immunization coverage stands at 80%, meaning there is still children under five who don’t get full immunization. The County will address this through strengthening community units and carrying out mobile clinics. **c**. There is prevention and reduction of drug and substance abuse following drug and substance abuse policy development. **d.** The County plans to increase the percentage of HIV positive pregnant Mothers accessing Prevention of Mother to Child Transmission (PMTCT) services as there is slow uptake of PMTCT. **f.** Improve maternal health services by increasing the percentage of pregnant women attending at least four antenatal cares including the percentage of deliveries conducted by skilled health workers which currently stands at 65%. | **b & d**. The county is promoting exclusive breastfeeding among children 0-6 months old and increasing uptake of children 6-59 months receiving Vitamin A twice a year. **f.** 28% of children under five years are stunted, 5% wasted and 10% underweight. **g**. There are school health programmes including nutrition, hygiene, and deworming **i**. In the current CIDP, the County will strive to promote nutrition education and strengthen the Community Units to offer broad based services to eliminate malnutrition cases. | **d**. Efforts are in place to increase the coverage of school feeding programme to improve the retention and transition rates to 100%. **e & f.** The county is promoting ECDE including supplying and fixing play equipment to schools and improving the quality, and transition rate of the ECDE learners. | **a**. The learning environment is being improved through equipping of ECD Centres, monitoring the proportion of ECD teachers with minimum qualifications and participating in co-curricular activities as well as number of schools benefiting through provision of instructional learning materials. **b**. The county is promoting ECDE including supplying and fixing play equipment to schools and improving the quality, and transition rate of the ECDE learners. **d.** There are four libraries that serve the residents of the County. **e.** The county government with support from stakeholders will continue to invest in ECD and health through infrastructural development, employment of ECDE teachers, provision of sanitation facilities and enhance school feeding programme. | **b**. The county plans on achieving SDG 6 by the year 2030 through investment in adequate water infrastructure and provision of sanitation facilities at all levels. **c**. As well as achieve access to adequate and equitable sanitation and hygiene for all **d**. There are plans to prevent use of pesticides and chemicals in farming, which leads to water and air pollution. **e**. The Directorate ensured water, air and land pollution control through establishing an anti-dumping and compliance taskforce unit at the county headquarters. **g.** Prevention and response to gender-based violence by monitoring uptake of services by sexual and gender-based violence survivors. **h.** The public benefits organisation is partnering with the County government to carry out advocacy on social inclusion issues as well implementation of programmes mainly in health, social protection and promotion of peaceful coexistence. **i.** The Cash Transfer for Orphans and Vulnerable Children was launched in 2004 to meet the needs of the country’s increasing number of children made vulnerable by poverty and HIV/AIDS. |
| 32.Nandi | Under 5 | The County has a total of 2,462 ECD centres with 79% of them being publicly owned. The total enrolment in ECD is 42,470, representing a Gross Enrolment Rate of 32%. The ECDE sub-sector employs a total of 2,035 teachers with the teacher: pupil ratio of 1:29. Owing to the high number of pupils ready to join pre-primary, the county Government is currently constructing 60 ECDE Centres. | **a**. The contraceptive prevalence in the county is 64.5% that is the proportion of women of the reproductive age who use contraceptives. **b.** 60.2% of children are fully immunized against the expected target of 80%. There is upscale on immunisation sensitisation to achieve this objective. **c**. There is a fight on drug and substance abuse including carrying out public awareness campaigns on effects of alcohol and drug abuse and building a rehabilitation centre for drug and substance addicts. **f**. 55.7% of expectant mothers attended first antenatal visits and only 24.3% completed forth visit in 2016. In the same reporting period, 42.3% of the expectant mothers were delivered by skilled personnel. **h & i.** The county is reducing the number of neonatal deaths by purchasing incubators and promoting Kangaroo Mother care. | **b**. The county is promoting breastfeeding and the proportion of children under 1 year who are exclusively breastfed is at 54%. **d**. The rate of utilization of Iron and Folic Acid Supplementation is low hence the need to enhance school nutrition for ECDE children including provision of Vitamin A supplement. **f.** The proportion of children under 5 years who are stunting stands at 29.9% as compared to 26% at the National level; those underweight stands at 11% while wasting rate is at 4%. Malnutrition is a challenge across the county with the most affected groups being infants, young children, and mothers. There is a need for programmes on improvement of the health and nutritional status of infants. | **b**. The county is reducing the number of neonatal deaths by purchasing incubators and promoting Kangaroo Mother care. **d.** There is need for programmes to improve nutritional status of infants, promoting breastfeeding and introduction of school feeding program in pre-primary schools. **e.** The county plans to equip all the public ECDE Centres with books and playing materials. **f**. There are construction of classrooms in ECD centres and employment of more pre-school teachers. | **a**. There is employment of ECD teachers, improvement of ECD infrastructure, provision of desks and other furniture, introduction of school nutritional programs and provision of teaching and learning materials. **b &** **e**. Owing to the high number of pupils ready to join pre-primary, the county Government of Nandi is currently constructing 60 ECDE Centres and have supplied them with books and playing materials. **d**. The county plans a construction of state-of-the-art county library to add to the two existing libraries. | **b**. The county is assisting in increasing safe water supply for domestic and farm use including borehole drilling and distribution as well as improving sanitation. **c.** There is creation of hygiene awareness through outreaches and forums as well as hygiene in schools and households. **d**. Collaborate with sector to implement workplace safety to prevent air pollution due to deforestation. **e.** The county has put in place measurers to control pollution from agro processing industries to maintain clean environment. **f.** The current CIDP will create spaces for recreation, open spaces and urban identities. **g**. The county is formulating Social Protection Policy including sensitization on gender-based violence, and people with disability and child rights. **h & i.** Establishing County orphan and vulnerable children fund that will transfer funds to needy households, strengthening children protections structures including Housing for severely vulnerable families. |
| 33.Narok | Under 5 (3-5yrs for pre-primary education) | There are 695 ECDE centres distributed across sub-counties with an enrolment of 69,030 learners. Currently, there are 1,388 ECDE caregivers distributed across county. The ECDE  centres have registered a transition rate of 89% and literacy rate of 67%. The teacher pupil  ratio currently stands at 1:50. | **a**. Approximately 33% of the population are using contraceptives compared to 58.0% of the national estimate. **b**. Immunisation coverage in the county is relatively low with fully immunised children at 48%, a drop from 58%. There is currently immunisation defaulter tracing and provision of services to hard-to-reach areas. **d**. Prevention of mother-to-child transmission (PMTCT) coverage is 77% in addition to 20 new PMTCT sites activated. **f.** Mothers attending first antenatal care (ANC) are at 95% while the mothers attending the fourth ANC clinic are 22%. Deliveries conducted by a skilled birth attendant are at 32%. This is being addressed through offering of incentives to mothers who deliver in the health facilities. **g.** Malnutrition in expectant mothers is on the rise contributing to high incidences of low birth weight, premature birth and congenital abnormalities. | **a**. Adult malnutrition especially that expectant mothers is on the rise contributing to high incidences of low birth weight, premature birth, congenital abnormalities. **f**. The nutritional status of children stands at 11.9% underweight 11.9%, 32.9% stunting and 2.4% wasting. **g**. Establish deworming programmes for schools. **i.** The county has serious health challenges resulting from malnutrition therefore, strategies are being put in place to promote sustainable community-based activities in the areas of agriculture, nutrition, and health education, to minimize malnutrition among children aged below 5 years. | **d.** Improving the quality of health and general development of ECDE learners through feeding programme to reach 20% of ECDE centres. **e**. Provision of teaching and learning materials to ECDE centres to enhance quality of teaching and learning in the centre. **f.** There is need to have quality ECD and therefore  the county government is playing a key role in ensuring that the education at this level is improved. This would mean investment in recruitment of more ECD teachers, building more ECD centres and providing enough learning and teaching material. | **a**. There is provision of teaching and learning materials to ECDE centres to enhance quality teaching and learning in the centre. **d**. To enhance literacy level and promote learning culture in the county, there is need to construct more library facilities across the county. **e.** Currently, there are 1,388 ECDE caregivers distributed across county. The ECDE centres have registered a transition rate of 89% and literacy rate of 67%. The teacher pupil ratio currently stands at 1:50. | **a**. The county plans to decentralize birth certificate toward sub counties for ease of registration. **b**. The priority of the water sub-sector is to increase access and availability of safe water by investing in water supply infrastructure development and rehabilitation, provision of sanitation services and protecting and conservation of existing water sources. **c.** Improving hygiene and reducing sanitation  related illnesses by constructing friendly toilets for ECDE learners, communities, and households. **d.** Control of air pollution, noise pollution, other public nuisances, and outdoor advertising. **e**. The county plans to provide a high quality of life to all citizens in a clean and secure environment free from pollution from agrochemicals. **f.** There is a safe, clean and green public space available to the public for  recreation. **g.** Prevention of sexual and Gender Based Violence cases and assaults. **h.** Social Transfer Programme gives a helping hand to caretakers of the most vulnerable, ultra–poor children promoting their physical and mental welfare. **i.** To cushion the vulnerable from poverty the County government and National government are undertaking cash transfer for Orphans and Vulnerable Children. |
| 34.Nyamira | Under 5 (3-5yrs for pre-primary education) | There are 414 ECDE centres spread across the county 1286 ECDE teachers recruited across the county | **a.** Only 65% of women access family planning services against the expected target of 80%. Family planning uptake for men is very low and undocumented. There is therefore a need to increase the use of contraception among women of reproductive age (15-49 years). **b.** The proportion of children below one year of age who are fully immunized has been maintained at over 84% over the last five years as well as and tetanus toxoid vaccination to all pregnant women. **c**. The county plans to control alcohol consumption, drugs and substance. **d.** The key drivers for curbing HIV and AIDS among children in the county is through prevention of mother to child transmission. **f.** Fourth ANC attendance is about 50% in the county while skilled care attendants in our health facilities gradually rose to 52% in 2017. **h**. Maternity facilities in all sub-counties have been equipped for pregnant mothers to access maternal health  care including antenatal care (ANC) from a skilled provider to monitor pregnancy and reduce the risk of morbidity for mother and baby during pregnancy and delivery. | **a.** The proportion of wasted and underweight children is negatively correlated with the level of education, health and nutrition status of the mother. **b**. Breastfeeding within 1 hour of birth is at 40.5% and exclusive breastfeeding for 6 months is 37.1%, which is low compared to the national level at 61%. **c.** The minimum acceptable diet recommends that breastfed children 6-23 months be fed foods from four or more food groups daily. Children 6-23 months receiving minimum meal frequency is 40.4% while nationally it is at 21%. **d**. Vitamin A coverage amongst children 6-59months is 39.4% against a target of 80%. **f**. 7.2 % of all children borne in the county are of low birth weight. 9.6% of children under five years compared to 12.9% were underweight, 25.5 % of children under five years compared to 25% were stunted. This is a serious national development concern as these children will never reach their full physical and mental potential. | **d**. Ensuring adequate breastfeeding practices and complementary feeding with ECD school feeding programmes. **e**. Provision of indoor and outdoor play materials to ECDE centres to enable children identify talents at an earlier stage for holistic development. **f**. The County therefore seeks to ensure that the ECD Programme focus on strategies like having well equipped ECDs, recruiting more ECD teachers, building more ECD centres and providing enough learning and teaching materials. | **a**. Ensuring inclusive and equitable quality education and promoting lifelong learning opportunities for all including monitoring the proportion of children under 5 years who are developmentally on track in health, learning and psychosocial wellbeing by sex. **d**. The county lacks a major library however, there are plans to put up libraries in each sub-county. Existing libraries are equipped with furniture but lacks adequate and relevant books. **e.** The County therefore seeks to ensure that the Early Childhood Development Prog focus on strategies like having well equipped ECDs, recruiting more ECD teachers, building more ECD centres, ECD school feeding programme and providing enough learning and teaching materials. | **b.** The county aims to increase accessibility to clean and safe water for domestic and industrial use from estimated 35 % to 70 % by year 2022 and to <0.5Km. Including availability and sustainable management of water and sanitation by monitoring the proportion of population using safely managed drinking water services. **c**. Improve hygiene system by ensuring access to adequate and equitable sanitation and hygiene for all and end open **d.** Control of air pollution, noise pollution, other public nuisances, and outdoor advertising. **f.** There are cultural activities, public entertainment, and public amenities, including County parks, beaches and recreation facilities. **g & h.** There is Tujikaze humanitarian program which does campaigns on gender-based violence, however, they are not enough and involved in every sector and therefore we need partners to upscale social protection. The county has implemented nationally appropriate social protection systems and measures for all by 2030 to achieve substantial coverage of the poor and the vulnerable. **i.** To address poverty levels, the national Government has formulated a program of cash transfer to the vulnerable members of society. However, these programmes are limited in coverage due to the limited resources and need enhancement. |
| 35.Nyandarua | Under 5 (3-5yrs for pre-primary education) | The County currently has 929 ECDE centres of which 496 are public and 433 are private. The enrolment is approximately 49.49% of the projected target population aged between 3 and 5  years. The Gross Enrolment Rate is 54.5%. The Net Enrolment Rate is 53% with boys being 54.2% and girls being 51.8%, indicating there are many children who are not enrolled in ECDE. There are 1317 ECDE teachers with a teacher pupil ratio of 1:26. There are 789 teachers in public centres and 528 teachers in private centres. | **a**. The contraceptive acceptance rate stands at 67%. The remaining 33% represent the unmet need for family planning. Counselling about side effects and mass media communication can improve acceptance rate. **b**. The immunisation coverage stands at 85.3% of the children in the entire County. Immunisation can be improved through sensitisation to overcome religious beliefs hampering immunisation. **c.** Reduction of alcohol and drug abuse cases is a priority for the county. **d.** The Prevention of Mother to Child Transmission and availability of antiretroviral drugs has increased the capacity of the County to reduce the spread of HIV/AIDS. **f.** 96.7 % of pregnant mothers seek first in antenatal care (ANC), while 58% seek fourth ANC from a skilled provider while 3.3% do not seek ANC services at all. 86.1% of mothers deliver in health facilities while 12.8% deliver at home and 85.3% of the deliveries are conducted by a skilled provider. | **a & b**. The main causes of malnutrition in the County include poor maternal, infant, and young child feeding  and breastfeeding practices. **c**. Only 37.4% of children aged 6 to 23 months receive adequate  dietary diversity and meal frequency. **d**. Micronutrient deficiencies are widespread among the children.  Maternal anaemia is a major determinant of child growth and only 6% of pregnant women receive the recommended 90 or more doses of iron/folate supplements. **f.** 29% of children less than five years in the County are stunted, 3 percentage points higher than the national average. On the other hand, 7% of the children are either underweight or overweight. The prevalence of wasting among the under-fives is 2%. | **d**. The county established school feeding programme to improve health, growth and development, to enhance retention and completion rate. **e.** Provision of play equipment in ECDE centres to improve holistic growth and development of the pupils is underway. **f**. The institutions need more qualified teachers, suitable classrooms, sanitation facilities, teaching/learning materials, feeding programme and requires a robust curriculum supervision and co-curricular activities programme to enhance learning. | **a**. The county is achieving quality education by ensuring inclusive and equitable quality education and promoting lifelong learning opportunities for all. **b.** The institutions need more suitable classrooms, play equipments and teaching/ learning materials **d**. The county plans to build community library to improve knowledge & literacy levels. **e.** One of the main programmes in the sector under this CIDP targets ECDs with the number of ECDE classrooms being rehabilitated and new ones built, staffing increased, teachers trained and employed on permanent and pensionable terms.  . | **b & c**. The County Government will partner with the National Government to invest in the provision of clean water for domestic use as well as for irrigation. The county aims to improve Sanitation which encompasses maintenance of personal hygiene, safe disposal of liquid and solid waste, control of disease vectors, provision of safe drinking water and provision of hygienic shelter. **d.** Control logging /burning of charcoal to prevent air pollution, degradation of the forest and wildfires, and over-reliance on wood fuel. **e**. Community  and institutional greening projects in environmental conservation to improve personal and environmental hygiene. **f.** There are two recreational trees parks situated within the Ol’Kalou urban centre for the public and tourist to visit. **h & i.** There is a Cash Transfer Programme orphaned and vulnerable children and persons with severe disability to ensure that the vulnerable members of the society can meet their basic needs and supplement the income they get from other sources. |
| 36.Nyeri | Under 5 | The gross enrolment rate in public ECDE centres for the county  stands at 59.2%, indicating that there is a large number of children who are not in school despite  having a total of 758 centres. The County has several childcare facilities distributed per sub-county as follows: Kieni West (3), Kieni East (3), Othaya (3), Mathira East (2), Mathira West (2), Tetu (3), Mukurweini (3) and Nyeri Town (6) | **a.** Access to family planning services/contraceptive uptake is at 73% with 211,878 women in the reproductive age group. **b**. Immunization coverage stands at 88.4% with 127 health facilities providing immunization services.  **c.** There are plans to prevent and control alcohol, drugs and substance abuse. **f.** The county is ensuring pregnant women attend at least 4 antenatal care visits whiles currently skilled deliveries stand at 88% with 36 health facilities providing delivery services in the county. | **f**. Nyeri County nutrition status stands at 15.1% stunting, 2.5 % underweight and 2.4 %wasting. Conduct Rapid Nutritional Assessment & Integrated outreaches by Monitoring of school health/diet and upscaling of High Impact Nutrition Interventions. **g**. Up scaling deworming and promotion of balanced diet programmes in public schools. **i.** To identify and tackle child malnutrition, the county will ensure that everyone can enjoy a safe and nutritious diet all year round. | **d.** Increase school feeding and deworming programmes to improve the health of learners. **e**. Renovation, Construction of Kitchen and toilets, Levelling of Playground and Fencing. Repair and rehabilitation of building and employment of teachers. | **a.** Renovation of ECDE classrooms; Provide conducive learning environment in ECDE centres. **b & d**. Promote reading culture in the county and ease in dissemination of information by constructing library and equipping it. **e.** Enhancing childcare and facilities including upgrading the children’s homes. The gross enrolment rate in public ECDE centres stands at 59.2%, with a total of 758 centres. | **b**. Maintenance of sanitation standard will ensure clean and safe water, clean environment reducing health related risks resulting in increase in households with access to affordable clean and safe water. **d**. Control of air pollution, noise pollution other public nuisance. **e.** There are plans to promote, conserve, and protect the environment. **f.** There are public entertainment and public amenities, including museums, sports and cultural activities facilities, and county parks, beaches and recreation facilities which are safe for family use. **h.** There are 1500 known Orphans and Vulnerable Children in the county and there are four major social net programmes. **i.** The county has 80 and 3,795 persons benefiting from the Cash Transfer Programmes for the persons with severe disability. |
| 37.Siaya | Under 5 | The County has 1083 ECD centres composed of 700 public and 383 private with an enrolment of 80,672 (40,462 boys and 40,210 girls). 657 ECD teachers are employed by the County Government; however, some are engaged by the communities. The teacher: pupil ratio is 1:33 while the transition rate is 94% | **a**. Family planning services are paramount in ensuring that women practice healthy timing and spacing of pregnancies and the county will require more resources to meet the rising demand for family planning. **b**. Currently only 150 health facilities are offering immunization services in the County. Fully immunized child coverage improved from 60% to 80%, which is still below the required national target of 95%. **c**. The county plans to establish a centre for the rehabilitation of drugs and alcohol addicts. **d**. Prevention of mother to child transmission **f.** There was increased 4th antenatal care (ANC) uptake from 40% to 50% and increased skilled delivery from 59% to 65%. Interventions are being put in place to increase skilled deliveries from 65% to 90% and 4th ANC from 50% to 80%. | **a.** Malnutrition is one of the contributing factors to morbidity and mortality especially in infants, children, and their mothers. **b & c.** Poor infant and young child feeding practices continue to prevail with low rates of exclusive breastfeeding (65.5%) against the National target of 80% of children under the age of six months, as well as late introduction of complementary food with poor dietary diversity. **d**. 81% Vitamin A supplementation for children 6-59months and 56.8% pregnant women supplemented with IFAS. **f.** There are 7.8% underweight children under 5years, 24.7% are stunting and 4.7% are wasting and **g.** 26.6% of children 12-59 months are dewormed. | **d**. The county plans to provide modest meal to all ECD learners by initiating the school milk programme and ensuring that ECD children in all the public ECD centres receive milk at least twice a week. **e & f.** Create forums for sensitization and capacity building of parents and guardian/care givers on positive mentorship and leadership skills, using role model parents and guardian for backstopping the ECDE programme and capacity build parents/caregivers with relevant skills, knowledge and attitudes to support children. | **a & e.** The county's opportunity for early learning includes construction of classrooms for the ECD centres to improve access, retention and transition to the next level; recruitment of ECD instructors to improve quality of instruction, increase access as well as equip existing ECDE Centres (learning resource & recreational material) to provide a conducive and quality of the learning environment and rolling out a school feeding programme in all the ECD centres for the pre-primary children. **d.** The county has Nyilima, Rambula and Ukwala community libraries. | **b**. The county is improving access to safe water and sanitation services. **c**. Maintaining hygiene through improving environmental sanitation by conducting clean up days, developing dump sites and procuring waste transport tracks. **d**. Control of air pollution, noise pollution, other public nuisances and outdoor advertising. **e**. The county envisions a clean and secure environment and efficiently using affordable clean energy and conserving the available natural resources. This would be achieved by **h**. Empowering communities to take care of orphans and vulnerable children and County Social Security and Services Sheltered workshops for persons living with disability (PLWD) to empower them with skills for self-reliance Orphans and Vulnerable children support to empower communities to take care of orphans and vulnerable children. |
| 38.Samburu | Under 5 | The total number of ECD centres in the County was 529 with a total enrolment of 42,938, 24180 boys and 18,758 girls with 470 teachers translating to teacher/pupil ratio of 1:91 as of 2017. The number of centres has now increased to 546. The number of ECDE teachers has also increased from 41 to 470. We intend to recruit at least 90 teachers every financial year. | **a**. Women of Reproductive Age receiving Family Planning commodities increased from 17.8% to 21.9%. **b**. Immunization coverage for Children under one year dropped from 72.3 % to 57.8%. The poor performance is attributed to frequent health worker unrests, some facilities not offering immunization services and lack of equipment in the newly constructed health facilities. **c.** The county plans to increase awareness on alcohol and drug abuse, and to **d**. strengthen prevention of mother to child transmission of HIV/AIDS by improving the 48.3% HIV+ pregnant mothers receiving preventive ARV’s. **f.** Deliveries by skilled delivery at the facility increased from 18.6% to 34% and Antenatal Care fourth visit from 47.3% to 49.6%. | **a**. More than three quarters of women (77.8%) in the county consume foods from less than the minimum recommended 5 food groups as per the FAO standards. **b**. Exclusive Breastfeeding currently stands at 69.4%. **d.** Vitamin A supplementation is at 47.6% for children 6-11 months while children 12-59 months (twice) are at 26.8% which is below the national target of 80%. Iron Folate Acid Supplementation for pregnant mothers is at 72% against the national target of 80%. **f**. The County has an overall stunting rate of 34.0% among children 6 -59 months with severe stunting rate of 10.6%. This is above the national stunting levels of 26%. Wasting rate for children 6 -59 months is at 18.3% with severe wasting at 3.8%. Prevalence of underweight in the county is at 34.3% with severe underweight at 7.0%. **g**. Children dewormed (once) is at 63.2% and (twice 32.8%). | **d.** Provision of County feeding to all ECDE centres and to trainee ECDE officers who can ensure proper safety and utilization of County feeding programme as well as strengthen home grown school meals programme and food safety and quality strategy in schools. **e.** Increased capacity of community, parents and other stakeholders to adopt new holistic child development service package models with 375 centres equipped with outdoor fixed play equipment. **f.** Procure and Supply of teaching/ learning materials. Capacity building of ECDE officers and teacher. | **a.** Ensure that quality assurance and standard are adhered to in all ECDE centres through capacity building of officers and teachers, preparation of teaching / learning support materials and smooth transition from ECDE centres to primary. **b**. Procurement of 300 outdoor fixed play equipment for games. **d**. Construction and equipping of county Library to promote reading culture. **e**. The total number of ECD centres in the County was 529 with a total enrolment of 42,938, with 470 teachers translating to teacher/pupil ratio of 1:91. | **a**. The county plans to strengthen formal birth registration including supporting OVCs to access protection services such as birth registration. **b**. To protect, conserve and improve access to adequate and safe water and other natural resources for a sustainable socio-economic development. The water Department also undertakes health education on sanitation, water treatment and safe storage before consumption. **c.** The county promotes hygiene standards in all towns. **d**. Control of air pollution, noise pollution, other public nuisances and outdoor advertising. **g**. Increase awareness on gender base violence by monitoring the proportion of new outpatient cases attributed to gender-based violence. **h**. There are formal social security provisions under the department of Gender and Cultural Development, Children’s Department and Development partners have been implementing safety net programmes targeting 32000 beneficiaries from the poor and vulnerable sections of the population including **i.** Cash Transfers for Orphans and Vulnerable Children and Persons with Severe Disabilities. |
| 39.Taita Taveta | Under 5 | The total enrolment in pre-school is 10,230, of whom 48% (5020) are boys and 52% (5,210) girls. The Net Enrolment Rate (NER) is 50.6% and 51.9% for boys and girls respectively. Transition rate to primary stands at close to 100%. The ECDE sub-sector generally lacks adequate learning and teaching materials, especially books for use at the early age, whilst no standard curriculum is followed. | **a.** The total demand for family planning is 59.7%. Women of the reproductive age receiving family planning commodities increased from 85% to 90%. **b.** Immunization rate for infants who are fully vaccinated between 12-23 months is at 68.1%. Those who have been vaccinated against measles and BCG are 84.8% and 92.4% respectively. **c**. The county is addressing drug and substance abuse issues. **d.** The number of HIV positive mothers receiving ARVs has increased from 376 to 402. Intermittent Preventive Therapy uptake remains low at 36%. Improving neonatal health by monitoring the number of integrated outreaches conducted. **f.** New Antenatal Care visits stands at 80.6% and mothers completing 4 anti-natal care visits stands at 58.1%. Delivery by skilled attendants increased from 58% to 62%. **i &** **k**. There is integrated management of childhood illness and health workers being trained on kangaroo mother care. | **a.** The number of pregnant and lactating women referred for malnutrition management are being monitored. **b.** The county plans to encourage and monitor number of children exclusively breastfed. **d.** Encourage and monitor number of children 6-11 months supplemented with Vitamin A. **f.** The nutritional status for children under 5 includes 34% for stunting, 11.2% for wasting, and 28.5% underweight, which is classified as malnourished. | **d.** The county plans to enhance school-feeding for ECDs including provision of milk and porridge. **e**. There is rehabilitation of ECD paly grounds and provision of play equipment. **f.** The county government has introduced a new curriculum which focused on pre-primary level of early childhood development education to cater for developmental and educational needs of the children between 4-5 years. The county also recruited ECDE teachers on permanent and pensionable basis and increased Enrolment in ECDE by 22,765 children which is a net enrolment of 86.4% in ECDE. | **a**. The county plans enhanced access, retention, completion and transition to primary level from ECD by infrastructure improvement, staffing, community sensitization, feeding program, growth monitoring, and teaching /learning materials. **d.** There is also increased access to reading materials, mobile library, and establishment of institution libraries and community sensitization. **e.** The ECDE sub-sector generally lacks adequate learning and teaching materials, especially books for use at the early age, whilst no standard curriculum is followed. The total enrolment in pre-school is 10,230, of whom 48% (5020) are boys and 52% (5,210) girls. The Net Enrolment Rate is 50.6% and 51.9% for boys and girls respectively. | **b**. The County has water from the forest sources which sustains livelihoods of the local communities and institutions, as well as those of regions beyond the county borders. An estimated 63,981 (about 86%) of the total households in the County have access to toilet facilities while about 14% of households do not have any kind of toilet facility. **c.** Access to hygiene and Sanitation facilities in the community, households and schools. **d.** Control of air pollution, noise pollution, other public nuisances and outdoor advertising. **e.** The county government is reducing environmental risks and ecological scarcities for sustainable development without degrading the environment. **f**. Leisure and recreation services are being provided at the community including provision of play equipment. **g**. Improve responsive care for gender base violence victims. **h & i.** Poverty alleviation through programs such as food for assets, educational improvement through provision of health improvement especially for Orphans and Vulnerable Children by supporting them through medical assistance, feeding programmes, and cash transfers to caregivers of these children. |
| 40.Tana River | Under 5 | The County has 322 public ECD centres, of which 167 are stand-alone ECDs while 155 are integrated with primary schools. There are 52 private ECDs. The total number of EDC teachers is 298. The teacher - pupil ratio in pre-primary school is 1:82. The total enrolment in public ECDs is 24,666 and 446 in private ECDs. The pre-primary retention rate is 87% with a drop-out rate of 13% while transition rate is 87%. | **a.** Uptake of family planning services is 34.3%, which is low due to the religious faith of the communities. Otherwise, all family planning methods are available in the county. **b.** The county has an average immunization coverage of 76% which is below the National average of 90%. The low immunisation coverage is attributed to nomadic lifestyle and frequent depletion of stock of the required antigens. **c.** Control drugs, alcohol and substance abuse. **d.** The county plans to improve access to elimination of mother to child transmission of HIV services. **f.** There are plans to increase proportion of mothers attending 4th antenatal Care (ANC) visit by ensuring all facilities and outreach sites to offer ANC services and increase proportion of mothers accessing skilled delivery services**. h.** The county will improve neonatal care in facilities without incubators as well as all facilities to offer **i.** Kangaroo care training to mothers and aiming to increase child health through Increased proportion of under-fives accessing comprehensive **k.** Integrated management of childhood illness services and to increase the number of units offering Integrated Management of Child Illnesses from 40% to 80%. | **a.** The county plans to improve the nutrition of residents, with particular attention to Children and pregnant women as well as implement nutrition programme. **b.** There are low exclusive breastfeeding rates in the county hence there are plans to train health worker and sensitize the community on breastfeeding. **c & e.** There is low complementary feeding among children 6-23 months hence there are plans to establish an inventory of all the locally available foods, sensitize mothers as well as train staffs. **d & g.** Integrate vitamin A and deworming in ECDEs, conduct coordination meetings and train all the ECDE centres on Vitamin A supplementation, deworming, and increase availability of Iron and folate to mothers at community level, introduce kitchen gardening in at least 3 hospitals, conduct quarterly coordination meetings. **f**. Conduct routine anthropometric assessments at the facility and community level using radio spots, community strategy, schools and religious leaders to create awareness on the importance of micronutrients to the community. | **b**. Kangaroo care training to mothers aiming to increase child health through increased proportion of under-fives accessing comprehensive **d.** There is low complementary feeding among children 6-23 months hence there are plans to establish an inventory of all the locally available foods, sensitize mothers on complementary feeds as well as train staffs on complementary feeding. **e.** The county is increasing learning material supplied to ECD centres as well as play and art materials. **f**. Renovate and establish ECD centres including ECDE teacher college to enhance the skills of teachers. Over 100 ECD centres were constructed across the county allowing early childhood education in areas where long distances from learning centres deterred high enrolment. | **a & e**. The county plan to provide play and art materials with 150 every year for 5 years at a cost of 162 million. Additionally, 50 million for learning materials over 5 years, and 150 ECDEs over 5 years linked to at a cost of 25mill the county has 315 ECDE centres with 462 teachers. The gross enrolment rate is at 56.3 per cent with a teacher pupil ratio of 1:51. **d**. Provision of Library Services to improve reading culture and literacy levels. | **a**. Accelerating /scaling up of birth registration for children under 5 years disaggregated by gender. **b**. To promote safe water utilization at household level with education on water treatment methods and to procure and distribute water treatment chemicals with water storage containers supported by partners. **c.** Conduct health promotion and hygiene sessions to community members with community dialogue days. **d**. Pollution control through procurement of air quality surveillance machinery**. e.** The County has not yet fully addressed challenges facing conservation of natural environment and sensitize residents to maintain cleanness of their environment of work and residence. **f.** Establishment of county public park to provide public recreational and rest areas to appreciate nature. **g.** Establishment of a One-Stop-Shop Sexual Gender Based Violence Recovery Centre and to monitor the percentage reduction in prevalence of violence against women, girls, boys and elderly including people with disability. **h & i**. Provision of social support to people with disability and other vulnerable group like Orphans Vulnerable Children to empower and improve their lives and welfare. The following social safety net programmes are offered in the County: Cash Transfer to Orphans and Vulnerable Children and Cash Transfer to Persons with Severe Disabilities. |
| 41.Tharaka-Nithi | 3 to 6 | The county has 577 ECD centres of which 432 are public and 145 are private centres. The County’s ECDE institutions enrol children from at an average of 3-6 years. The total ECDE enrolment is about 24,000 composed of 10,000 girls and 14,000 boys in public and private centres. Most of the private ECDE Centres are community managed but outside the mother primary schools while others are managed by churches. | **a.** The proportion of women receiving family planning commodities stands at 58.7%. **b**. The proportion of immunization coverage for children under one year who are fully immunized is 57%. **c.** The county shall also strengthen the prevention and treatment of substance abuse, including narcotic drug abuse and harmful use of alcohol. **f.** Proportion of pregnant women attending 4 antenatal care (ANC) visits stands at 39.8%, those who attended at least one ANC visit during pregnancy is 62.2%, as well as delivery by Skilled attendant coverage is 44.6%. | **d & g.** Deworming and Vitamin A supplement for children 6-11 months once stands at 46.9% and Vitamin A for 12-59 at least twice is 34.4% and 72.2% respectively; Deworming at least twice stood at 14.9. | **d**. Provision of feeding programme in all ECDE centres is underway. **e**. Employment of more teaching staff, provision of quality learning materials and up scaling the schools feeding programmes and purchasing of teaching /learning and play materials as well as putting up infrastructure. are necessary interventions to improve the learning situation. **f**. Parents and communities therefore had to pay levies to cater for the management of ECDE centres. There is need for employment of more ECDE teachers and caregivers as well as increased funding to increase access and ensure quality facilities. | **a & e.** The county plans to purchase learning materials and equipment with increased focus on ECDE to ensure that all girls and boys have access to quality early childhood development, care and pre-primary education so that they are ready for primary education. **d**. The county has a library at Ganga ward. All the other wards do not have a library. Efforts are being made to ensure that libraries are started in every ward in the county. | **a**. The government established Huduma Centre at Chuka, which has facilitated easy access to services like issuing of birth certificates. **b.** The county aims to provide clean and safe water and improve sanitation by improved sewerage systems and increase households with piped water from 60% to 80% by 2022. **c.** By 2030, the county aim to achieve access to adequate and equitable sanitation and hygiene for all and end open defecation, paying special attention to the needs of women and girls and those in vulnerable situations. **d**. Control of air pollution, noise pollution, other public nuisances and outdoor advertising.  **e**. There is implementation of specific national policies on natural resources and environmental conservation, including water services to maintain healthy and clean environment. **g**. The county plans to build capacity and sensitize communities on gender violence. **h.** Children protection issues are being undertaken by the County government in partnership with NGOs like Plan International, and others to ensure that issues of children rights like child marriage and female genital mutilation are addressed properly. **i**. The numbers of orphaned children in the county are on the increase and the county provides cash transfer programme for orphan’s children. |
| 42.Trans Nzoia | Under 5 | The public ECDE centres in the county stood at 715, 385 for the public ECDEs and 330 for private ECDEs. The enrolment at the end of 2017, stood at 57,859 with 28,948 boys and 28,950 girls respectively. | **a**. Contraceptive uptake is low at 56.4% of women aged between 15-49 years. This CIDP seeks to inform strategies that will further drive up the coverage of modern contraceptive methods amongst women of childbearing age. **b.** Despite all public facilities offering immunization services, the percentage of fully vaccinated children aged 12-23 months is 55% and the national average is 75%. Hence more resources are being made available to boost the current immunization efforts. **c.** Establish a drug and substance abuse rehabilitation centre to cater for drug dependent persons in the county. **d.** The level of prevention of mother-to-child transmission coverage has increased over the years to 6.7% against the national average of 8.5%. **e**. Construction of Mental Health Unit to assess and improve the overall mental health of the county’s population is a priority for the government. **f.** The percentage of women receiving antenatal care (ANC) from a skilled provider is 92%. This has been enhanced through sustained health education on the importance of ANC attendance. **g.** Implement Neonatal Health Services to reduce number of morbidity and mortality among new-borns and monitor the % of new-borns who have received essential New Born Package. **k**. Integrated Management of Childhood Illness to improve the management of children at primary care level health facilities and reduce mortality, illness, disability among children under five years is a priority for the government. | **a.** Abnormal pre-pregnancy BMI scores are associated with poor birth outcomes and obstetric complications. It is therefore important that the county takes deliberate efforts to promote a healthy lifestyle for its population for nutrition and enrolment. **f.** The prevalence of underweight children is 15.3%. This is above the national average of 11%. Stunting in children is at 29.2%; three percentage points above the national average of 26%. 3.9% of children are wasted. **g**. Promote deworming for children. **i.** The county plans to integrate the management of acute malnutrition in routine health services at all levels of healthcare provision. There is reduced incidence of under-nutrition due to early detection and intervention. | **d**. Feeding program for ECDEs to boost enrolment. **e.** Provision of playing equipment to improve pupil’s physical development and overall balanced growth. **f**. Employment of at least 360 Caregivers in Public ECDE centres, with supervision to ensure compliance to set standards, including provision of furniture for ECDE to create a conducive learning environment. **g**. Construction of Mental Health Unit to assess and improve the overall mental health of the county’s population is a priority for the government. | **a.** The county plans to construct and equip ECDE classrooms in every public ECD Centre; supply teaching and learning /instruction materials to ensure inclusive and equitable quality education and promote lifelong learning opportunities for all. **b.** Provision of playing equipment. **d**. Construction of county public library, equipped and commissioned to improve reading culture and informed society. **e**. The Directorate of ECDE is mandated to undertake supervision of ECDE curriculum, capacity building of ECDE teachers and improving the physical environment of the ECDE children through construction of classes and provision of outdoor play equipment. There is construction and equipping of 3 ECDE classrooms in every public ECDE centre with provision of furniture | **b.** Implement the Kitale Water Supply and Sanitation project to increase access to clean,  safe and portable water, and safe disposal of wastewater. **c**. Improving sanitation and hygiene always has a significant impact on health both in households and across communities. **d**. Control of air pollution, Noise pollution, other public nuisances. **e**. County  prepared to tackle key issues including environmental degradation, encroachment of environmental conservation to ensure a clean environment. **g.** Mitigating against sex and gender-based violence is a priority for the county. **h.** The county has various social safety net programmes aimed at empowering the community members and improve their livelihoods. These include women development fund, County Government Bursary and at the National level, Women Enterprise Fund, National Government Affirmative Action fund, **i**. Cash Transfer fund including Disability Cash Transfer and Orphans and Vulnerable Children Cash Transfer. |
| 43.Turkana | Under 5 | The number of ECD centres in the County is 682 with an enrolment of 101,094 pupils. This is attributed to the increasing number of children who are under five years of age enrolling in these centres. Most of these centres do not have adequate facilities. | **a.** The uptake of contraceptives currently stands at 40%, which has been attributed to intensified advocacy and campaigns. The uptake of family planning services has led to the reduction of sexually transmitted diseases. **b**. Immunization coverage is 30%, which is attributed to inadequate cold chains for storing vaccines at the existing health facilities, including inadequate staffing, and inability for health staff to reach members of the community among others. **c**. There is establishment of recovery and rehabilitation centres for drug and alcohol abusers in the 7 sub counties to promote a healthy and responsible society through controlling the sale, use and abuse of alcohol and illegal drugs. **d**. The county plans to improve participation in comprehensive care of HIV positive women identified through Prevention of Mother to Child Transmission and establish mobile clinics to create community awareness on safe motherhood. **f.** Antenatal care service uptake is at 50% and uptake of skilled delivery is currently at 6%. | **a**. The county plans to reduce malnutrition among pregnant and lactating mothers and the under 5 years and to **b**. Scale up High Impact Nutrition Interventions for of children under six months on exclusive breastfeeding to improve quality of life. **c.** Nutritional statuses of children within the county are extremely low, as there is over-reliance on maize as a source of carbohydrates and beans for proteins thereby ignoring fruits and vegetables, which are vitamin rich nutrients. **f.** Stunted growth is a primary manifestation of malnutrition in early childhood, including malnutrition during foetal development brought on by the malnourished mother. **g.** Promote nutrition and deworming of children. **i.** Emergency measures include providing deficient micronutrients through fortified sachet powders or directly through supplements. | **d**. The county plans to scale up of High Impact Nutrition Interventions for children under six months on exclusive breastfeeding to improve quality of life, as well as **e.** providing infrastructure like classrooms, play equipment and materials, Staffing- Teaching and non-teaching staff (care givers), including **f**. Training of teachers and non-teaching staff for ECDE and conducting annual education campaigns in the 7 sub counties; construction of at least 5 model ECDE centres per ward and recruiting at least 300 ECDE teachers per annum. | **a & e.** There are 682 ECD centres in the County with an enrolment of 101,094 pupils. This is attributed to the increasing number of under-fives enrolling in these centres. It may also be due to continued advocacy on importance of getting basic education. Training of teachers and non-teaching staff for ECDE and provision of play materials. **d.** There are plans to establish and equip modern library to promote literacy of children. | **b & c**. The county is establishing water management systems to desalinate and treat harvested water from Lake Turkana to provide clean and safe water for all, as well as to increase equitable and sustainable access to improved sanitation and hygiene services. **d.** Control of air pollution, other public nuisances and outdoor advertising. **e.** There is the need to control activities to maintain a clean environment. **g**. The county plans to establish Sexual and Gender based violence (SGBV) Programme for effective coordination between GBV service providers as well as functional SGBV clinic providing clinical and psychosocial services. **h & i**. Turkana County Social Protection Programme established with cash transfer and grants for widows and women and other vulnerable groups. Poverty alleviation cash transfer programme is disbursing funds to vulnerable groups (OVCs, people living with disability) among others. |
| 44.Uasin Gishu | Not mentioned | A total of 557 ECDE classrooms were constructed, and 117 feeder schools established which led  to increase in enrolment from 25,162 in 2013 to 32,297, in 2017. A total of 1019 ECDE  teachers were recruited and deployed translating to pupil teacher ratio of 1:32 which is below  the desired plan target of 1:20. | **a.** Family Planning Coverage among women in reproductive age currently stands at 58% with plans to increase those receiving Family Planning Commodities to 80%. **b.** Immunization coverage improved and currently stands at 72.6% of under 1year fully immunized with 84% of health facilities offering immunization services. **c.** There is establishment of Alcoholic drinks, drugs and substance abuse centre to rehabilitate victims. **d.** Mother to Child Transmission of HIV facilities currently stands at 81% with plans to offering PMTCT Services to 90%. **f.** Women attending 4 ANC Visits stands at 42% with plans to increase to 75%. Deliveries by skilled health personnel stands at 62% with plans to increase to 85%. In addition, percentage of pregnant women attending 4 ANC clinics improved from 36% to 49.1%. | **d**. There is plan to upscale school health programs like micronutrient supplementation (Vitamin A) and **g.** deworming. **i.** Establishment of nutrition screening outreaches. | **d**. The county plans to establish School feeding programs in partnership with stakeholders. **e & f**. including recruitment and deployment of ECDE teachers, provision and equipping of ECDE facilities and in-service training for ECDE teachers. The county also targeted to increase ECDE enrolment rate to 76%, construct classrooms and upgrading of stadia and playing fields. Provision of  teaching and learning materials to all public ECDE Centres to improve quality of teaching  and learning. | **a**. The aim of the County is to ensure all eligible children who are not in school at the pre-primary level are enrolled. Currently the total enrolment at the ECD level stands at 32,297. **d**. And to provide library to improve literacy. **e**. Some of the strategies will include investments on ECD educational facilities such as building additional classrooms and equipping, hiring of teachers, and provision of teaching and learning materials to ECDE centres and to equip all ECDE centres in the county. | **b**. The county has prioritised to provide clean, accessible and adequate water within reasonable distance and to construct/extend sewer line to reach more households with rehabilitation and maintenance of sewer lines. **c.** Provision of hygiene facilities and monitoring of schools participating in promoting health through personal hygiene and disease prevention. **d**. Effective pollution control measures in the areas of noise, water, and air pollution. **e.** Maintain clean and sustainable environment through the provision of efficient and effective solid waste management services. **g**. There are plans to establish Sexual & Gender Based Violence facilities to support victims and policies to control cases. **h**. As well as empower women in the county financially and improve the nutritional needs of their  families as a total of 1,474 women groups were supported with 218,000 chicks through the Inua mama na Kuku programme. |
| 45.Vihiga | Unclear | Enrolments in early childhood education improved since 2013 with current enrolment of 41,278.  This can be attributed to improved access to ECD infrastructure and awareness campaigns on ECDE. | **a.** The awareness of family planning services in the county is above 90% with contraceptive usage at 56.6%. A total of 25.8% of married women of ages 15-49 have unmet need for family planning. Opportunities are being provided to improve reproductive health with increasing access to family planning services. **b.** The immunisation coverage for the county for the children under 5 is 80.2%. **c**. The county plans to undertake alcohol and drug abuse survey, using the results to formulate a strategy and implement the same. **d.** HIV positive pregnant mothers receiving preventive ARVs improved from 82.4% to 99.1%. **f.** Free maternity services were implemented recording improvement in skilled deliveries from 38% to 51%. The average attendance of fourth ANC visit improved from 48% to 60 %. | **b**. There was an increase in exclusive breast feeding from 26% to 65%. **c.** Promote cultural foods and indigenous vegetables  production to improve food  security and nutrition **d**. The percentage of children under five years with stunting dropped from 23.5% to 14.6% due to increased nutritional supplements. **f.** In the county, 21% of the total population of children under the age of five are either underweight, stunted or have wasting condition. Of this, 3.8% are underweight, 14.6% stunting and 2.6% wasting. | **d.** The county plans to introduce school feeding in all ECDE Centres, nutrition programme, and promote cultural foods and indigenous vegetables. **e & f.** Establishment of playground and completion, equipping and staffing of all ECD Centres. Establishment of an ECD centre of excellence in  each sub county to improve access to education and set standards of development. | **a & e.** The county plans a construction, completion and equipping of new and existing ECD centres to promote access to ECDE. Establishment of an ECD centre of excellence in each sub county to improve access to education and set standards of development is also a priority including establishment of playground and completion, equipping and staffing of all ECD Centres. **d.** There are also plans for acquisition of land for construction and equipping of communal library. | **b.** The county intends to improve access to clean and safe water and sanitation services by investing in water infrastructure, sanitation and environmental management conservation programmes through the Belgium Government to expand water schemes. **d**. The county plans to control air and other pollution including **f**. Construction and equipping of recreational facilities including parks and playfields. **g**. Establishment of gender-based violence centres to minimise gender-based violence as well as sensitise the citizenry on dangers of gender-based violence. **h.** The county is issuing health insurance cards to community health volunteers and vulnerable households under the department of social services social protection programme. The county poverty level is at 39 % compared to national’s 45%. **i.** To reduce the poverty level the County and National Governments have put in place Poverty Eradication Fund, Uwezo Fund, Cash Transfer, and the Vihiga County Community Empowerment Fund, among others. |
| 46.Wajir | 4 to 6 | ECD centres are 264 with a total enrolment of 15,075. The teacher/pupil ratio is 1:25 and transition rate of 90%. Current enrolment for ECDE for children between 4-6 years category is 18,800 whereby 10,642 are boys and 8,158 are girls. In addition, the provision of quality education to this group hinges on establishment of more ECDE centres and recruitment of more teachers. | **a.** Family planning and contraceptive prevalence is very low with only 2% of married women using modern methods of family planning. This low uptake of family planning is attributed to cultural beliefs and practices in the community. **b**. 38% of the children aged 12-23 months received all recommended vaccinations. There are initiatives towards providing immunization services closer to the people through installation of solar powered cold chains in rural health facilities, mobile clinics and mass immunization campaigns. **d.** Prevention of Mother to Child Transmission of HIV is being monitored including the proportion of HIV positive expectant women on ARV. **f.** The ratio of mothers seeking minimal 4 antenatal care visits stand at 38%. Mothers delivering at health facilities stands at 18% of total deliveries. There is need to improve on the community referral services to help expectant mothers to easily access health services. **k.** Training and capacity building on Integrated management of childhood illness to improve childhood managements is a priority. | **b.** There are sensitization among the communities on nutrition issues including proper breast feeding and balanced diets. **c & e.** Malnutrition is highly pronounced in the rural settlements where access to nutritional food products is a challenge. Moreover, there is promotion of family farming through organic practices thereby enhancing food security at household level. **d.** In addition, the government needs to provide nutrition supplements to school going children and other vulnerable populations.  **f.** 26% of children under age five in the county are stunted and there is need for deliberate efforts to reduce this figure. **g**. Monitor the proportion of school children dewormed every 6 months. | **d**. Establish school feeding programme to improve enrolment and retention as well as distribute value added sorghum to schools under school feeding program. **e.** The county plans to provide adequate staffing for the caregivers, provision of teaching and learning materials and supply of indoor and outdoor play materials. **f.** There is also capacity building for ECD care givers and adoption a scheme of service for ECD care givers to improve quality of education. | **a.** The county plans to ensure inclusive and equitable quality education and promote lifelong learning opportunities for all including adequate staffing for the caregivers, provision of teaching and learning materials and supply of indoor and outdoor play materials. **b.** Promotion of cultural knowledge through provision of reading materials in identified resource centres. **d**. There are 4 community libraries which needs to be fully equipped as well as operationalize them. **e**. There is increase in the enrolment and retention in early childhood development and education, increase retention of children in ECDE centres. The teacher/pupil ratio is 1:25 and transition rate of 90%. | **a**. The county facilitates registration of births for all children delivered at health facilities. **b**. There is increased access to clean and safe water through water resource assessments and mapping, investment in underground & rainwater harvesting and adoption of proper technologies for water retention including improved level of sanitation. **c**. Improve provision and access to sanitation systems and promote hygienic practices throughout the county. **d**. Control pollution by developing county specific legislations on occupational health and safety and health care waste management. **e**. There is need to improve on the negative effects on the environment given that most parts have poor drainage and experience floods during rainy seasons. **f.** Setting up of recreation center for public use and **g.** Develop and operationalize County Gender Based Violence policy and establish a gender-based violence recovery centres as well as gender desk to support victims. **h & i.** The county continues to improve social protection and livelihoods of vulnerable groups including eligible households with OVCs and PWD receiving cash transfers. |
| 47.West Pokot | 0 to 5 | There are 1,032 pre-primary school centres in the county with 838 ECD teachers. ECDE enrolment currently stands at 77,679 giving teacher pupil ratio of 1:92. This enrolment represents approximately 51% of the total eligible number of ECD school age children under this age bracket. Low enrolment is contributed by nomadic lifestyle, long distances to schools and lack of enough feeding program. | **a**. The County’s contraceptive prevalence rate dropped from 14.2% to 12.2%. This remains a major challenge due to cultural beliefs, religion and low levels of education. **b**. The immunization coverage for the fully immunised child under one year dropped from 58.1% to 43%, which is below the national target of 85%. These were attributable to the protracted industrial action by nurses and inadequate cold chain facilities. **d.** There are plans to carry out partner mapping for the formation of Technical Working Group in prevention of mother to child transmission. **f**. The percentage of women who attended four ANC visits dropped from 21.3% in to 13.0%. This was also associated with a drop in delivery by skilled birth attendant from 45.3% to 32.0%. | **c.** Provision of feeding programs through the ECDE centres with milk supply and ECDE centres benefit from nutritional supplements. **d**. Pregnant women are receiving iron supplement with children 12-59 months receiving Vitamin A supplement. **f**. There are children under 5 years attending Child Welfare Clinics for growth monitoring. nutrient supplementation and **g.** deworming programs for children | **d**. Provision of feeding programs through the ECDE centres with milk supply and ECDE centres benefit from nutritional supplements. **e.** There is provision of instructional material, furniture and play materials in the ECDE centres, including **f**. Construction of model ECDE classrooms, employment of additional qualified ECDE teachers and supervisors, equipping (furniture and outdoor play items) of ECDE centres, feeding of needy ECDE learners and training and development of ECDE teachers. | **a.** Low enrolment in ECD is contributed by nomadic lifestyle, long distances to schools and lack of enough feeding program. **b**. Equipping of ECDE centres with furniture and outdoor play items. **d.** Construction and equipment of 20 Pokot Cultural Libraries to improve learning and reading. **e.** The priority areas under ECD include construction of additional classrooms, recruitment of more ECD teachers, provision of school feeding programme and installation of water tanks to ECDE schools. | **a**. The county provides legal identity for all including birth registration. **b & c.** It also plans to achieve universal and equitable access to safe and affordable drinking water for all, including access to adequate and equitable sanitation and hygiene for all, improve water quality, increase water use efficiency across all sectors and timely repair of leakages and enforcement of hygienic laws. **d.** Controlling air pollution, outdoor advertisement, and other public nuisance. **e**. There will be continuous sensitization of the community on the benefits of forests and clean environment to raise awareness and promote clean environment. **f**. There are recreational facilities in major centres per sub counties for public access. **g.** including promotion of Sexual Gender Based Violence (SGBV) through gender empowerment, creation of awareness on SGBV and linkage of SGBV survivors to health and legal services. **h & i.** There is an expanded and scalable cash transfer infrastructure established in 13 Arid Counties including Hunger Safety Net Programme to protect and support chronically food insecure households, households safeguarded from drought and hunger, including Bursary and cash transfer program for the orphans and the vulnerable including people with disability and widows. |
