# Supplementary material for: Improving early childhood development in the context of the nurturing care framework in Kenya: A policy review and qualitative exploration of emerging issues with policy makers
Source: Front Public Health. 2022 Sep 27;10:1016156. doi: 10.3389/fpubh.2022.1016156 (PMC9551223; doi:10.3389/fpubh.2022.1016156)
Supplement: Supplementary file 2 [file Table_2.DOCX]

**Supplementary Table 2: Data extracted from Kenyan national policies**

| **National policy documents dating from 2010** | **Age (0 to 5)** | **Centre-based care in relation to under-fives (ECD/ECDE)** | **Nurturing Care Framework** | | | | |
| --- | --- | --- | --- | --- | --- | --- | --- |
|  |  |  | **Good health** | **Adequate nutrition** | **Responsive caregiving** | **Opportunities for early learning (ECD/ECDE)** | **Security and safety** |
| 1. The Framework for the National Child Protection System for Kenya November 2011 | Unclear | N/A | **b**. Protect children from disease through better sanitation and immunization.  **c.** Ensure through NACADA1 that drugs/harmful substances are kept away from children.  **j.** The Medical services ministry provides health services, creates an enabling environment, regulates, and sets standards and policy for health service delivery to children. | N/A | **i.** In this framework, Nongovernmental Organizations (NGOs), Community-based Organizations (CBOs), and Faith-based Organizations (FBOs) pursue activities to relieve suffering, promote the  interests of children, provide basic social services, or undertake community development. While CBOs operate within a small geographical area, NGOs can be national and FBO often, besides spiritual matters do work in similar to that of CBOs and NGOs. | **e**. There are plans to register learning institutions and monitor the quality of programmes, as well as establish and maintain institutions such as day nurseries and clinics for  Children. | **b**. There are plans to meet children’s basic rights including food and nutrition, health, shelter, education, clean and safe drinking water among other related rights. **g**. Ensuring human rights and social justice are realised by instituting criminal proceedings against persons who commit crimes against children and to report violence and exploitation against children. **h**. Provision of legal aid and a guardian for any child involved in a court case and who needs this support. |
| 2. The National Plan of Action Against Sexual Exploitation of Children in Kenya  2013 - 2017 | Unclear | N/A | N/A | N/A | **h**. Awareness raising and sensitization of parents and religious leaders on sexual  abuse and exploitation of children. **i**. A Call for Action to Prevent and Stop Sexual Exploitation of Children and Adolescents (2008) calls upon states, with the support of international organizations, NGOs, FBOs, CBOs, Civil Society and the private sector to establish and implement robust frameworks for the protection of children and adolescents from new forms of sexual exploitation. | N/A | **g**. There are plans to decentralize the Child Helpline (116) to the community level and to sensitize the local community and tourists on the existence of the Child Helpline to which visitors can call to provide information to authorities on local and international sex tourists. |
| 3. Policy Guidelines for Management of Diarrhoea in Children Below Five Years in Kenya August 2014 | Unclear | N/A | N/A | **b.** There is promotion of early and exclusive breastfeeding **d**. The policy recommends the following fluids: Cereal gruel (UJI), Fresh and fermented milk, Soups prepared from meat, fish and chicken, Oral Rehydration Salts and Breast milk. **h.** Parents and other caretakers of children under five will be empowered to give early treatment at home to children with diarrhoea following the four main rules of home therapy: To increase fluid intake to prevent dehydration when diarrhoea starts, breastfeed more frequently and for a longer time at each feed, at least eight times a day and night and give ORS  **i.** All children with diarrhoea shall be given an appropriate dose of Vitamin A at the facility if they have not received a dose. | **i.** Appropriate financial mobilization by all partners in the health care provision, medical training institutions, faith-based organizations, the pharmaceutical industry, development partners and the community to support children. | N/A | **b**. There is improved water supply quantity and quality, including treatment and safe storage of household water.  **c**. Promotion of handwashing using running water and soap particularly the four critical times (Before Preparing the food, before feeding the baby, after visiting the toilet, and after changing baby's napkins). |
| 4. A National Framework and Plan of Action for Implementation of Integrated Community Case  Management (ICCM) in Kenya 2013-2018. A Strategy for Management of Childhood Illnesses  in Under Five Years  August 2013 Edition | Unclear | N/A | b. Immunization is important, it saves lives, and every child needs  to complete immunization in the first year of life. Take your baby to the health facility for the first immunizations (BCG and oral polio) within 2 weeks after birth. | **b**. Exclusively breast feed a baby for 6 months without giving any  food or fluids as breast milk protects the child from diseases like diarrhoea, pneumonia  **c.** Introduce nutritious foods at 6 months such as thick uji made  from any type of cereals such as maize, sorghum, millet enriched with sugar, milk, groundnuts,  margarine and fat  or oils; and continue breast  feeding up to at least 24months. | **a.** Keeping the baby in skin to skin contact with the mother helps baby to get heat from the mother’s body (kangaroo mother care) | N/A | **a.** Ensure that birth of your child is registered and collect the birth certificate as this is a child’s right and helps government in planning for services such as: schools,  medicines, food. **b.** Treat water to make it safe for drinking by methods such as boiling, chemical treatment, filtration and 3 pot  system, and store drinking water in clean covered containers. **c.** Wash hands with soap and running water at the 4 critical times: after visiting toilet, before eating, before cooking/preparing food, after wiping a child faeces (after defecating/changing baby  nappy) and safely dispose faeces (including those of children) into a pit. |
| 5. Laws of Kenya, Children Act, Chapter 141 Revised Edition 2012 | Unclear | N/A | **b**. Among the parental responsibility is the duty to maintain the child and in particular to provide medical care, including immunization. | **c.** Among the parental responsibility is the duty to maintain the child and to provide adequate diet. | N/A | **b.** A child shall be entitled to leisure, play and participation in cultural and artistic activities.  **e.** Every child shall be entitled to education the provision of which shall be the responsibility of the Government and the parents. Every child shall be entitled to free basic education which shall be compulsory in accordance with Article 28 of the United Nations Convention on the Rights of the Child. | **a**. Proof and identity of motherhood shall be required in the form of birth Certificate, immunization card, baptismal card, etc. **g**. The Director of Children’s Services shall give attention and help the acute situations  of children in hardship, including children who are sexually abused and children who are affected by domestic violence, and formulate programmes for the consideration by the Council, for the alleviation of the plight of such children. **j.** Where a child has, by virtue of a care order, been committed to a rehabilitation school or to a charitable children’s institution, the Director in conjunction with the manager of the institution may place the child with a foster parent, for such period as the Director may from time to time authorise. |
| 6. National Nutrition Action Plan 2013 – 2017  Ministry of Public Health and Sanitation | Unclear | N/A | N/A | **a.** Optimal maternal nutrition is crucial for the health and development of both the foetus and the mother. It has further been shown to have an impact on birth outcomes, with better nourished mothers having increased chances of delivering healthier infants. **b**. The Lancet series 2016 show that improving breastfeeding practices could save 823,000 deaths and would prevent 20,000 cases of cancer among mothers annually. **d & e.** Enhance systems for delivery of micronutrient supplementation, uptake of diversified, and bio-fortified foods and promote compliance, production and consumption of fortified foods. **f.** Develop capacity for improved screening and referral of acute malnutrition at community and health facilities **i.** Integrate micronutrient deficiency prevention and control measures within public health systems  through provision of supportive policy environment for micronutrient supplementation and advocate for joint planning with nutrition-sensitive sectors to  contribute to strengthening of agri-nutrition capacities and coordination at national and county levels. | N/A | N/A | **b.** Advocate with WASH sector to promote establishment of WASH facilities and provision of safe drinking water and promote adequate WASH in households and institutions. |
| 7. National Plan of Action for Children in Kenya  2015 - 2022 | Unclear | N/A | N/A | **a**. Optimal maternal nutrition is crucial for the health and development of both the foetus and the mother. It has further been shown to have an impact on birth outcomes, with better nourished mothers having increased chances of delivering healthier infants. **b.** Exclusive breastfeeding combined with optimal complementary feeding has the potential to avert up to 19% deaths for under-five thus improved child survival. **c.** Optimal complementary feeding has the potential to avert up to 19% deaths for under-five thus improved child survival. **d**. Enhance systems for delivery of micronutrient supplementation and enhance uptake of routine micronutrient supplementation (vitamin A, iron and folate and micronutrient powders) for targeted groups. **e**. Promote compliance, production and consumption of fortified foods**.**  **i.** Most cases of acute malnutrition are managed in the supplementary feeding programme and outpatient therapeutic programme while about 7% of cases with severe acute malnutrition receive inpatient care before being discharged for outpatient care programmes. | N/A | N/A | **b & c.** Kenya is committed to an upscale of WASH under SDG 6 on achieving universal and equitable access to safe and affordable drinking water, access to adequate and equitable sanitation and hygiene and an end to open defecation for all. **h**. Social protection policies and programmes hold immense potential for improving the nutrition situation of vulnerable populations. To ensure that these policies holistically combat malnutrition, a nutrition-sensitive approach needs to be employed in their design and implementation. |
| 8. National Standards for Best Practices in Charitable Children’s Institutions  April 2013 | Unclear | N/A | **b.** All children below 5 years should receive mandatory vaccinations and growth monitoring as required by the Ministry of Health. **l.** As part of the assessment, a child should receive a full medical screening/ examination by a qualified medical practitioner on admission to a Charitable Children Institutions (CCI). The examination must include audio (hearing), sight and emotional needs assessment. **m.** If CCI admits children with disabilities, there must be a provision for appropriate appliances, assistive devices and services for the care of the children  There is a training programme for parents and caregivers and rehabilitation of children with disabilities or any other child with a special health need, such as terminal illness or chronic health conditions. | **f.** All children below 5 years should receive mandatory vaccinations and growth monitoring as required by the Ministry of Health | **h**. Empower the child’s family to fulfil their role in meeting the social and emotional needs of their child. This would involve the CCIs facilitating the involvement of parents in caregiver-led support groups and training them on knowledge, attitudes and skills that promote these. | **a.** CCI must ensure that Children have access to information on their rights and responsibilities as well as guided access to computers and telephones with restrictions to harmful internet sites. A children’s club or council is established, managed and chaired by the children and a member of staff designated to oversee the activities. **b**. All children have a right to leisure and recreation. A monthly leisure plan for all the children in the CCI should be drawn by staff and agreed on by CCI management. Experienced and qualified supervisors should always be present during leisure activities that may be risky such as swimming. **e**. Every child is entitled to free primary education. Although education at the ECD level is not free in Kenya, CCIs have the responsibility of ensuring that children under their care access ECD education. Children should attend community schools near the CCI to promote interaction with the community. | **b.** Providing access to clean water, sanitation and promotion of hygiene will ensure that children grow in a healthy environment and that infections, disease outbreaks and spread are controlled. There is clean and safe drinking water that is sufficient to meet the needs of both the staff and children, hand washing water and soap are provided in/near all the toilets and eating place. **c**. Children are taught and acquire good general and personal hygiene habits. |
| 9. Kenya National Social Protection Policy  June 2011  Ministry of Gender, Children and Social Development | Unclear | N/A | N/A | N/A | N/A | N/A | **h.** Social protection has been implemented in Kenya in many different forms for many decades, including various programmes created in response to emergencies. The establishment of the National Social Security Fund and the National Hospital Insurance Fund in 1965 and 1966 respectively, was part of the Government’s efforts to cushion workers against future vulnerability. **i.** The Government and its development partners are currently implementing several social assistance interventions targeted to specific categories of beneficiaries. **j**. Children of poor and vulnerable families will enjoy income security at least at the poverty level through family/child transfers aimed at helping them to access nutrition, education, and healthcare. |
| 10. National School Health Strategy Implementation Plan  2011 - 2015 | Unclear | N/A | **c.** Provision of specialized staff in children’s institutions to detect and deal with drugs and substance abuse**. m.** Disability and special needs are major impediments to effective learning, social inclusion and integration. Subsequently children with disabilities and those with special needs may always tend to remain in the lower social stratum of communities. | N/A | N/A | N/A | **b & c.** Healthy and hygienic school environment is actualized by safe, adequate water supply, adequate sanitation and appropriate hygiene promotion. The health benefits of safe and adequate water, improved sanitation and hygiene are broad in scope, ranging from reductions in diarrhoea, intestinal worms, ecto-parasites, infections and trachoma, to enhanced psycho-social well-being afforded via such factors as the dignity that goes with using a clean toilet/latrine. |
| 11. Guidelines for The Alternative Family Care of Children in Kenya  October 2014 | Unclear | N/A | **m.** The DCS should ensure that the foster families are linked with therapeutic, respite, health and psychological services, and that the grants given to them are sufficient to provide care needed and support to child with special needs. | N/A | N/A | N/A | **i.** There is a Cash Transfer Programme that can serve as a monitoring mechanism for families caring for non-biological children and OVC committee and the Cash Transfer Beneficary Welfare Committee members can monitor and supervise the children to ensure that they are receiving appropriate care and protection. **j.** Evidence have shown that institutional care has a negative impact on a child’s social, emotional, cognitive and intellectual development. The experience is particularly damaging for children under three as a family is a more nurturing, caring environment for a child to be raised in. Growing up in a family helps a child to develop a sense of self-esteem and belonging, family values, religious and cultural identity. |
| 12. Kenya Reproductive, Maternal, Newborn, Child and Adolescent Health (RMNCAH) Investment Framework  January 31, 2016  Ministry of Health | Unclear | N/A | **a.** Address supply side barriers for contraceptives method mix, efficient distribution systems, and competency-based training and update using WHO medical eligibility for contraceptive use for nurses, clinical officers and doctors. Ensure contraceptive commodity security and adequate financing for contraceptives  **k.** Scale-up community supported Integrated Management of Childhood Illnesses (cIMCI) such as pneumonia, malaria, and diarrhoea Implement competency-based training and skills retention for nurses, midwives and clinical officers in the management of facility based IMCI | **b & c.** Scale-up sustained behavior change communication for promotion of breastfeeding and appropriate and timely complementary feeding is being promoted. **d.** Promoting the delivery of essential nutrition services including micro-nutrients (iron and folic acid, vitamin A, multiple micronutrients and zinc) to pregnant/lactating women and children**. g.** Continue school deworming program for children (2-5 years) with 2 doses each year. | N/A | N/A | **a.** There are plans to link birth registration to integrated national identity cards. |
| 13. Footprints Children’s Home Child Protection Policy  2017-2018 | Unclear | N/A | **c.** Use of alcohol, cigarette or any drug substance within Footprints Children’s Home (FCH) premises or when accompanying children outside the children’s home is unacceptable. **f**. Any child who gets pregnant within FCH attends all antenatal and post-natal services. | N/A | N/A | N/A | **a.** No child shall be given a name similar (especially surname) to that of FCH personnel or board of trustee’s members and their birth certificates should not bear the name of any employees, member of board of trustees or any other person who is not the biological parent of the child. The FCH should be aware that in cases of adoption, a birth certificate, issued by registrar of Birth and Deaths**. b**. The FCH will ensure the welfare of children in relation to medical care, shelter, food, clothing, safe drinking water, education and reintegration of a child back to the family is provided as per individual care plan of the child. **g**. FCH shall protect all children in their care against harmful cultural practices. In Kenya, such activities include child marriages, female genital mutilation and cutting, traditional circumcision rites for boys that expose them to unhygienic and health risks including death and sexual relationship with adults or other children**. j.** Any form of foster care of child in FCH should be done in collaboration with the area District Children Officer. |
| 14. The National Children Policy  2010  The National Council for Children Services | Unclear | N/A | **a & b.** Provision of services and information on the importance of quality family planning, Ante-Natal Care, safe child delivery, breastfeeding, immunization and Post- Natal Care. **c.** Children in Kenya deserve to be protected from drugs and substance abuse. This can be achieved through accessible and affordable rehabilitation and rescue centres, equipped with  qualified personnel and equitably distributed countrywide for children who abuse drugs and other substances. d. In addition, special measures ought to be put in place by the government and all duty bearers to mitigate the impact of HIV/ AIDs and other diseases on children and embrace reproductive health concerns. **f.** It shall be the obligation of the Kenya government to provide adequate and quality health services to all children during ante-natal and postnatal periods and throughout the child’s lifetime.  **k.** Measures to prevent and manage childhood illnesses, disabilities, injuries and domestic accidents should be put in place. **m.** All children, especially children with disabilities and those with special needs have a right to be protected from any harm that may interfere with their growth and development. | **b.** Provision of services and information on the importance of quality family planning, safe child delivery, breastfeeding and good nutrition, with **d.**  Measures to reduce micronutrient deficiencies. | **i.** Although community care, adoption, foster care and charitable children’s institutions have been found to be viable alternatives for childcare, all children living under these arrangements shall be protected against any possible abuse and exploitation. This shall be achieved by provision for CCIs to operate as the last resort and temporary measure for children as they await appropriate placement and alternative family care within the community. | N/A | **a**. Every child particularly the child with disabilities and that with special needs has a  right to identity and registration at birth. This is guaranteed through naming, right to  nationality and the continued preservation of identity. **b.** There is provision of equitable access to quality and adequate educational  facilities with safe drinking water and separate sanitation facilities for boys and girls. **c.** Provision of services and Information, Education and Communication on environmental hygiene and sanitation. **j**. All children have a right to be protected and receive support within the family, community and the wider society. Therefore, there is the need to strengthen and support structures and community system take care of the orphans & vulnerable children as well as provision of treatment, care and support to children including their parents and caregivers. |
| 15. Sector Policy for Learners and Trainees with Disabilities  May 2018  Ministry of Education | Unclear | N/A | **l.** National Committee on Educational Objectives delved into the issue of special needs education and provided significant recommendations on early identification and placement, integration of learners into regular schools and provision of regular curriculum. **m.** The attainment of quality education for learners and trainees with disabilities is largely dependent on the provision of specialized human, institutional and community capacity development for experts at all levels of education for learners and trainees with disability. | N/A | N/A | N/A | **b**. The government will provide adequate, clean, and safe water and sanitation. |
| 16. Kenya Health Policy 2012 – 2030  Ministry of Medical Services  &  Ministry of Public Health and Sanitation | Unclear | N/A | **c.** Cases of alcohol poisoning were reported during the previous policy period, and more than 2% of all deaths in the country were attributed to alcohol use. | **b.** Breastfeeding practices have also improved, with exclusive breastfeeding for up to six months showing significant improvement. **d.** Promote control of micronutrient deficiency diseases and disorders through intersectoral collaboration.  **i.** Nutrition policy will be developed and implemented to reduce the burden of malnutrition, control nutrition-related diseases in the population, and promote the growth and survival of children. | N/A | N/A | **c.** Promoting good hygiene and sanitation to control food and water-and food-borne diseases. **h**. The State shall provide appropriate social security to persons who are unable to support themselves and their dependants. |
| 17. National Maternal, Infant and Young Child Nutrition  Policy Guidelines 2013 | Unclear | N/A | **a.** Counsel mothers to seek family-planning services. **b**. Mothers should ask about baby’s immunization schedule. Immunizations protect babies against several disease. **d**. Mothers who test HIV positive need to be counselled and provided with appropriate information to make informed choices on appropriate infant feeding. The HIV pandemic and the attendant risk of mother-to-child transmission of HIV through breastfeeding continue to pose unique challenges to the promotion of breastfeeding. **f**. Pre-pregnancy nutrition influences a woman’s ability to conceive, determines the foetal growth and development and the size of the foetus and its overall health as well as the health of the mother. **g**. Underweight and overweight women experience more complications during pregnancy and delivery than normal women. Anaemic women are more likely to deliver low birth weight infants and low folic acid levels are associated with an increased risk of low birth weight and birth defects.  **h.** Strategies to encourage children to eat more during an illness includes encouraging the child to drink and to eat with lots of patience, feeding small amounts frequently, giving foods that the child likes most and give a variety of nutrient-rich foods which are well prepared and attractively served and continue to breastfeed.  **i.** Kangaroo mother care (KMC) is care of a small baby who is continuously carried in skin-to-skin contact by the mother and exclusively breastfed (ideally). Encourage the mother to breastfeed more frequently when the child is ill. The nutrients and immunological protection of breast milk are important to the infant when mother or infant is ill.  **l.** The child’s ability to sit in a good, safe position for feeding, and their ability to use their jaw and mouth to get food into their mouth, move it around, and swallow it safely are some of the things to look out for in children to identify any disabling conditions**. m.** Children with disabilities are at increased risk for under-nutrition due to a myriad of emotional, physical, and social stresses. For many children with special needs, especially those with particular medical problems, one of the primary goals of eating is to get proper nutrition. Because these children may need special diets, it is important to make sure that nutritional needs are being met, regardless of what other strategies are used to help with mealtimes and feeding. | **a.** Good maternal nutrition is important for a successful pregnancy, child delivery and lactation. Pre-pregnancy nutrition influences a woman’s ability to conceive, determines the foetal growth and development and the size of the foetus and its overall health as well as the health of the mother. **b.** Give new-born infants no food or drink-- no water, no infant formula other than breast milk unless medically indicated. Support the mother to attach and position the baby to initiate breastfeeding immediately within 1 hour after delivery. **c.** Introduce complementary feeds at six months and continue breastfeeding for 2 years or longer, starting other foods in addition to breast milk at six completed months helps a child grow strong and healthy. Complementary foods should be introduced gradually in addition to breast- milk or other forms of milk. **d.** The micronutrient status shows that 42% of the women have Vitamin A deficiency. Iron deficiency among women is at 43% out of whom 70% are pregnant women. Moderate to severe anaemia is high among pregnant women. According to a USAID report 2007, 46% of mothers took iron supplements but only 2.5% used the supplement for 90 days as recommended. **f**. The most used method for monitoring and assessing growth is weight gain. Until breastfeeding is established, or if the baby is sick or small. Babies weighing 1.5 to 2.5 kg may lose up to 10% of their birth weight in the first four to five days after birth, and babies weighing less than 1.5 kg may lose up to 15% of their birth weight during the first 7 to 10 days after birth. **h & i.** Malnutrition is a direct or indirect cause of 54% of all childhood deaths. Many severely malnourished children are sent for inpatient care only after they become seriously ill. By then, they have severe wasting and/or oedema and very often have complications. Malnutrition affects the functioning of body organs hence special care is needed. Case fatality rate for severe malnutrition is high, going up to 50% or more. Prompt and proper management of severe malnutrition will not only reduce mortality but also the impairment of growth and psychosocial development of children. | **a.** Skin-to-skin with mother keeps the newborn warm and helps stimulate bonding or closeness, and brain development. This is done by assisting the mother to place the baby on her tummy immediately after delivery.  **b.** Kangaroo mother care (KMC) is care of a small baby who is continuously carried in skin-to-skin contact by the mother and exclusively breastfed (ideally).  **c.** Ensure there is rooming in, thus keep the baby with the mother in the same bed for unlimited breastfeeding, give new-born infants no food or drink, no water, no infant formula other than breast milk unless medically indicated.  **d**. Ensure active/responsive feeding for Young Children by encouraging the child to learn to eat with lots of patience, introducing one food at a time to reduce confusion and to identify the foods the baby is allergic to and let the child eat from his/her own plate (caregiver then knows how much the child is eating). Seat down with the child, be patient and actively encourage him/her to eat. **h.** Ensure family involvement, especially male partners in antenatal and postpartum breastfeeding and complementary feeding education**. i**. Foster the establishment of breastfeeding support groups and other support groups and refer mothers to them on discharge from hospital or clinic | N/A | **b.** Provide safe, clean drinking water and ensure that it is always available. |
| 18. National Pre-Primary Education Policy Standard Guidelines  2018  Ministry of Education | Unclear | N/A | **b**. Pre-primary Teachers usually conduct basic health checks and keep records such as growth monitoring, and immunization and other illnesses. In cases where children have missed some immunizations, they ask the parents to take them to the health Centre for necessary advice or action. **k.** Integrated Management of Childhood Illnesses **l.** Integrate early identification of children with special needs in the teacher  training curriculum. **m**. The Pre-primary education teachers should be well trained on the developmental needs and milestones in order to carry out early identification of children with special needs and disabilities as soon as possible and provide a supportive environment so that every child can achieve optimum physical, cognitive, emotional and  social development. | **d**. ECDE programs provide opportunities for the provision of specific health and nutrition interventions such as vitamin supplementation, referrals for treatment, screening and better health seeking behaviours among others.  **f.** Pre-primary Teachers usually conduct basic health checks and keep records such as growth monitoring, and other illnesses.  **g**. Nutritional education at the school, deworming and growth monitoring. | **h.** Parental education on parenting skill and positive discipline is crucial**. i.** There are various stakeholders participating in the provision of pre-primary education services including Parents, Communities, National and County governments, the Private Sector and Faith Based Organizations among others. The role of stakeholders is to participate in the development of policies, promote and support pre primary  education, supply goods and services and identify and promote peaceful  coexistence initiatives. | **a.** Teachers and other stakeholders should ensure safe learning environments that will provide each child with adequate opportunities to experiment, explore dialogue, discover, reflect and apply new knowledge, skills and attitudes. **b.** Actively encourage the child to play with caregivers, toys and other children for cognitive development. **e.** Empirical studies attest to the importance of investing in a child's early years for optimal realization of their potential in learning and development. Children who participate in quality and relevant pre-primary education programmes are better prepared for primary education. Moreover, quality pre-primary education programmes not only improve school readiness but also result in reduced repetition, dropout rates and increased learning achievements. | **b & c.** Availability of clean water, promotion of hand washing and proper sanitation at the pre-primary schools positively impact on the health of a child deterring waterborne diseases and infections. **f**. Create a conducive environment that encourage child play. **g.** Children exposed to violence, abuse and neglect are more likely to suffer from psychological problems including stress as well as internalizing and externalizing behaviours. This has been shown to affect the learners' self-regulatory and executive functioning which are key school readiness skills. In this regard, all teachers,  caregivers and child protection officers are obliged to ensure that every child enjoys their  prescribed rights in all settings. |
| 19. Kenya Community Health Policy  2020 - 2030 | Unclear | N/A | **a.** Counsel mothers to seek family-planning services. **b.** Promote immunization coverage to minimize communicable diseases. **c & e.** Referral for rehabilitative and counselling services for drug and substance abuse**.** Increase awareness on how to prevent mental illness and avoid using harmful substances such as alcohol, drugs and tobacco. **f**. Conduct ante-natal visits to advise mothers on early initiation & exclusive breastfeeding and refer for post-natal care. Conduct post-natal visits as per the national guidelines and screen for postpartum danger signs. **h.** Assess, identify and refer new-borns with danger signs. **j**. Raise awareness and counsel on danger signs of a sick child and when to seek care. **l.** Active case finding and referral for children with delayed milestones and/or disabilities. **m.** In particular community health personnel shall advocate or promote special devices that allow for people living with disability to live a dignified and productive life and to support efforts to eliminate stigma and discrimination. | **a.** Counsel on maternal nutrition  **b.** Promote, protect and support exclusive breastfeeding for the first six months of life and sustained breastfeeding for the first two years and beyond within the community. **d.** Referrals for micronutrient supplementation.  **e.** Promote use of improved home-based recipes and preparation methods for locally available foods, including home fortification. **f.** Conduct growth monitoring for under-fives**. i.** Screen, identify and make referrals for malnutrition**.** | **d & e.** Encourage responsive and nurturing care for the child including play, stimulation and communication, delayed milestones, child neglect and abuse. **i.** Motivation of community members, family support to continue providing support and enhancing social community safety nets. | a. Encourage responsive and nurturing care for the child including play, stimulation and communication, delayed milestones, child neglect and abuse. | **b & c.** Promote health education at household and community level by ensuring households and communities have access to clean safe drinking water and good hygiene practices such as hand-washing among others.  **e.** Promote community led total sanitation by mobilising the community, linking with environmental health personnel**. h.** Link vulnerable children to social and child protection programs. **j.** Motivation of community members, family support to continue providing support and enhancing social community safety nets. |
